# Supplementary material for: An integrated self-optimizing programmable chemical synthesis and reaction engine
Source: Nat Commun. 2024 Feb 9;15:1240. doi: 10.1038/s41467-024-45444-3 (PMC10858227; doi:10.1038/s41467-024-45444-3)
Supplement: Supplementary file 1 — Supplementary Information [file 41467_2024_45444_MOESM1_ESM.pdf]

Supplementary material for

An Integrated Self-Optimizing Programmable Chemical Synthesis and Reaction Engine

Artem I. Leonov[1†], Alexander J. S. Hammer[1†], Slawomir Lach[1], S. Hessam M. Mehr[1], Dario Caramelli[1], Steven O'Sullivan[1], Davide Angelone[1], Aamir Khan[1], Matthew Craven[1], Liam Wilbraham[1] and Leroy Cronin[1]*

[1]*School of Chemistry, the University of Glasgow, University Avenue, Glasgow G12 8QQ, UK.*

1 The Chemputer Optimizer Framework. 2
1.1 Architecture 2
1.2 Special XDL steps 2
1.3 Workflow 6
1.4 Optimization configuration 8
1.5 Interactive mode 9
1.6 Compilation 10
1.7 Spectra analysis 14
1.8 Novelty analysis 15
1.9 Algorithm interface 18
1.10 Summit Server 20
1.11 Data management 20
2 Analytical Labware 23
2.1 Magritek Spinsolve NMR 24
2.2 Agilent 1260 Infinity II LC 24
2.3 Ocean Insight QE Pro Raman spectrometer 25
3 ChemPU process monitoring 25
3.1 SensorHub 25
3.2 Process sensors 26
3.3 Software architecture 29
3.4 Computer vision 30
3.5 Dashboard 32
4 XDL Dynamic Step 33
4.1 Dynamic Addition 33
4.2 Dynamic Transfer 34
4.3 Dynamic Execution 35
5 Experimental section 37
5.1 General experimental remarks 37
5.2 Python script to load and run experiments 38
5.3 Sensor experiments 39
5.4 Dynamic procedure execution 46
5.5 Closed-loop reaction optimization 50
6 Appendix A 66
7 Appendix B 67
8 Appendix C 92
9 Appendix D 98
10 Appendix E 112
References 117

1 The Chemputer Optimizer Framework.

1.1 Architecture

The `ChemputerOptimizer` software package is based on the existing XDL framework, taking the advantage of its routines for procedure preparation (i.e., inserting implied steps and attributes) and execution. The core class, `OptimizeDynamicStep`, is a controlling the dynamic iterative execution of the given procedure and updating its parameters with the new suggestion from the optimization algorithm. Several classes are developed to support the closed-loop experiment execution, mainly: `AlgorithmAPI` – general class to provide interface for existing algorithms for optimization, manages reaction parameters and results, and translates them in an appropriate format; `SpectraAnalyzer` – class to analyze data from analytical instruments (spectra) and calculate the target reaction descriptor (e.g., yield); `OptimizerClient` – client part of the server-client based interaction with Summit and Olympus optimization frameworks.

1.2 Special XDL steps

On top of common steps to describe synthetic operations, `ChemputerOptimizer` features several special XDL steps for running closed-loop optimization.

1.2.1 OptimizeStep

The `OptimizeStep` step indicates optimization parameters in the XDL procedure (Fig. S1). These parameters are then processed during the procedure compilation with the hardware graph. They can be set by the user or are naturally extracted from the procedure, using a predefined set of common optimization steps, e.g., reagent addition or reaction temperature. Should wrap the step which parameters are subject to the optimization (child step).

```
<OptimizeStep
  id="1"
  optimize_properties="
    {'volume':
      {'max_value': 0.3, 'min_value': 0.0}}">
  <Add
    vessel="reactor"
    reagent="cyclohexenone"
    volume="0.2 mL"
    aspiration_speed="3.0"
    port="0"
    stir="True"
    priming_volume="0.2 mL" />
```

Fig. S1. Example of the `OptimizeStep` wrapper. The `optimize_properties` attribute indicates the parameter of the child step subjected to the optimization, with its `max_value` and `min_value` limits.

1.2.2 Analyze

The `Analyze` or `FinalAnalysis` step performs the reaction analysis when executed (Fig. S2). It contains all necessary parameters to run the analysis via Raman, HPLC or NMR instruments, including reaction sampling, sample treatment (e.g., dilution) and instrument control. During the compilation the step's attributes are additionally checked for consistency (e.g., "dilution volume" if dilution is required) and necessary callback functions (i.e., to update the optimization state upon iteration completion) are assigned. Should be inserted in the procedure after the reaction is complete.

```
<Analyze
  vessel="reactor"
  method="NMR"
  sample_volume="2.5"
  method_props="{
    'protocol': '1D FLUORINE HDEC',
    'protocol_options': {
        'centerFrequency': -85,
        'PulselengthScale': 1,
        'decouplePower': 0,
        'AcquisitionTime': 1.64,
        'RepetitionTime': 15,
        'PulseAngle': 90,
        'Number': 64
        }
      }"
  force_shimming="True"
  cleaning_solvent="THF"/>
```

Fig. S2. An example of the `Analyze` step to perform the NMR analysis of the reaction mixture upon reaction completion. The step contains attributes to indicate the vessel where the analysis should be performed, desired method for the analysis and sample volume (if sample is required). Additional attributes are added for the corresponding analytical method (e.g., shimming for the NMR) and the `method_props` attribute represents the parameters (e.g., nuclei for the NMR) to run the analysis.

When the reaction time is used as a parameter for optimization, it is important to add quenching steps to avoid inconsistency between the actual reaction time and the recorded reaction time. Reactions were typically quenched either by rapid dilution or addition of reagents during work-up steps during the analysis. These are part of the high-level `Analyze` XDL steps. For Raman, no quenching is needed as the measurement is instant. Therefore, time inconsistencies are minimal systematic errors. Executing the optimized, versioned XDL codes on our platform leads to reproducible optimal results.

1.2.3 Background reaction monitoring

The `StartMonitoring` asynchronous step starts the execution of the analysis with the chosen method in a background thread. Every recorded spectrum is saved in the `SpectraAnalyzer` class and is subjected for the analysis to categorize the found peaks into:

- Product – peaks that appeared during the reaction and remained at the end of the it.
- Starting material – peaks that were present at the start of the reaction and their integration area decreased during its course.

- Intermediate material – peaks that appeared during the reaction but were absent in the end of it.
- Noise/artifacts – peaks that were persistent during the reaction.

In the current implementation only Raman analysis is supported.

1.2.4 Constrained Step

In some cases, additional constraints beyond upper and lower bounds are imposed on the optimization variables. For example, all factors in mixture experiments must sum up to 100%. The `ConstrainedStep` wraps a child step and adjusts the value of a given parameter as a function of the values of selected `OptimizeStep` steps and the desired target value (Fig. S3). In the given example, the volume of the `Add` step is calculated as in:

$$\mathrm{V(target)} = \mathrm{V(id1)} + \mathrm{V(id2)} + \mathrm{V(Add)}$$

```
<OptimizeStep
  id="1"
  optimize_properties="
    {'volume': {'max_value': 20.0, 'min_value': 10.0}}">
  <Add
    vessel="reactor"
    reagent="cyclohexenone"
    volume="10 mL" />
</OptimizeStep>
<ConstrainedStep
  ids="[1, 2]"
  parameter="volume"
  target="50"
  <Add
    reagent="toluene"
    vessel="reactor"
    volume="99999 mL"
  <Add/>
<ConstrainedStep/>
<OptimizeStep
  id="2"
  optimize_properties="
    {'volume': {'max_value': 20.0, 'min_value': 5.0}}">
  <Add
    vessel="reactor"
    reagent="pyrrolidine"
    volume="10 mL" />
</OptimizeStep>
```

Fig. S3. An example of the `ConstrainedStep` step that adjusts the volume of a constrained `Add` step based on two `OptimizeStep` steps. In this example, the toluene volume would be adjusted to 30 mL.

1.3 Workflow

The overall process to run the iterative reaction optimization on a XDL compatible hardware platform (e.g., ChemPU) is as follows:

1. Prepare the chemical procedure as XDL file.
2. Wrap steps that are subject for the optimization with `OptimizeStep` step (as described in section 1.2) to mark the corresponding attribute.
3. Prepare the graph containing mapping to the hardware.
4. Prepare the configuration file, containing all necessary parameters to run the optimization (e.g., number of iterations) and load the algorithm (e.g., its name and related parameters),

and specifying parameters to perform reaction analysis and calculate the product descriptor (e.g., product peak at the final spectrum).

5. [Optional]. Prepare table with previous data (e.g., experiments run manually) as csv file, containing header with labels, that correspond to the parameters for the optimization.

6. Execute the optimization procedure on the physical platform.

Example code to run the optimization on the ChemPU is shown in Fig. S4.

```
from pathlib import Path

from AnalyticalLabware import devices

from chemputeroptimizer import ChemputerOptimizer

# ChemPU specific
from chempiler import Chempiler  # Control package
from AnalyticalLabware.devices import chemputer_devices  # Analytica
l instruments
import ChemputerAPI  # Low level API

# Path to the corresponding experiment folder
# Should contain the following files:
# procedure.xdl - XDL procedure
# graph.json - hardware graph
# optimization_conf.json - configuration file for the optimization
# (optionally) previous_results.csv - table with previous results
path = Path('folder')

# Instantiate main optimizer object
co = ChemputerOptimizer(
    procedure=path.joinpath('procedure.xdl'),
    graph_file=path.joinpath('graph.json')
)

# Run necessary preparations
co.prepare_for_optimization(
    opt_params=path.joinpath(
        'optimization_conf.json').as_posix()
)

# Upload previous results if given
co.load_previous_results(
    results=path.joinpath('previous_results.csv')
)
```

```python
# Create chempiler object to control the hardware
c = Chempiler(
    experiment_code='test',
    graph_file=path.joinpath('graph.json').as_posix(),
    output_dir='test',
    simulation=True,  # False if running on physical hardware
    device_modules=[ChemputerAPI, chemputer_devices]
)

# Run the optimization
co.optimize(c)
```

Fig. S4. An example code to run the optimization on the ChemPU using *ChemputerOptimizer* and *Chempiler* packages.

1.4 Optimization configuration

The configuration file contains all necessary information to run the optimization and calculate the reaction outcome in a json format.[1] The description of each parameter is given below, and an example of the configuration file is shown in Fig. S5.

- `max_iterations` – maximum number of experiments to perform within optimization run.
- `target` – indicates the calculation for the reaction outcome. Detailed description is given in the section 1.6.3. The value for each target attribute dictates when the optimization is terminated.[2]
- `algorithm` – indicates the algorithm to be used and its respective attributes. Details of the available algorithms and their parameters are given in the section 1.9.1.
- `reference` – indicates the peak position of the reference compound (i.e., internal reference for the analysis). Is used to reference the spectrum and calculate the reaction outcome.
- `constraints` – lists regions on the reaction spectrum to consider when calculating the final reaction outcome. In the current implementation, the intermediate result (e.g., area under the curve for the target peak) is divided by the sum of integration areas for all listed regions.
- `batch_size` – number of batches to perform the optimization in parallel.

```json
{
    "max_iterations": 5,
```

```
    "target": {
        "spectrum_integration-area_-80.809..-82.049": Infinity
    },
    "algorithm": {
        "name": "random",
        "random_state": 42
    },
    "reference": -113.15,
    "constraints": [
        "-73.214..-73.751",
        "-73.889..-74.653",
        "-75.178..-76.334"
    ],
    "batch_size": 1
}
```

Fig. S5. An example of the configuration file for the optimization.

1.5 Interactive mode

In the lack of an analytical instrument, or during the simulation it is possible to run the optimizer in interactive mode. In this case user will be asked to insert the necessary values for the target parameter when the corresponding analysis is executed (e.g., at the end of the reaction or work-up). This mode is executed automatically, if the `Analyze` step is absent in the XDL procedure, or if the corresponding "interactive" attribute for the `ChemputerOptimizer` is set to true. It is also possible to give the optimization parameters in the interactive mode, instead of wrapping necessary steps with `OptimizeStep`, where these steps are added internally. An example of user questions is given in Fig. S6.

```
Found step "Add" at position <0>,
with following properties:
-----
{'vessel': 'reactor', 'reagent': 'water', 'volume': 30.0, 'viscous':
False, 'dropwise': False, 'time': None, 'stir': False, 'stir_speed':
250.0, 'purpose': None, 'speed': 40.0, 'port': None, 'through': None,
'move_speed': 40.0, 'aspiration_speed': 40.0, 'dispense_speed': 40.0,
'anticlogging': False, 'anticlogging_solvent': None,
'anticlogging_solvent_volume': 2.0, 'anticlogging_reagent_volume': 10.0,
'through_cartridge': None, 'reagent_vessel': None, 'waste_vessel': None,
'vessel_type': None, 'anticlogging_solvent_vessel': None,
'prime_n_times': 1, 'priming_volume': 3.0, 'cleaning': False,
```

```
'cleaning_solvent': None, 'cleaning_repeats': 1, 'comment': ''}
-----
Would you like to pick it for optimization? [n], y
y

Add step has the following parameters for the optimization:
>>> volume

Please type one of them
volume
Current value for volume is 30.0
Please type maximum value for "volume": 50
Please type minimum value for "volume": 10
Any other parameters? ([n], y)


You are running FinalAnalysis step interactively.
Current batch is "batch 1"
Current procedure is running towards >{'final_parameter': inf}< parameters.
Please type the result of the analysis below
***as <target_parameter>: <current_value>***
final_parameter: 1
```

Fig. S6. Top: an example of the user question to create a step subjected for optimization (volume for the water addition step) and set the limits for its parameter. Bottom: an example of interactive question to input reaction result (as illustrated with `final_parameter`).

1.6 Compilation

In addition to the compilation process of the XDL procedure into the ChemPU instructions (described in [3], ESI) `ChemputerOptimizer` features several operations for internal preparations. Core operations include: identifying or assigning steps for the optimization and analysis, parsing configuration and assigning callback methods to calculate reaction outcome, scheduling the procedure for parallel execution, breakdown of the XDL steps into corresponding low-level substeps and mapping those to the hardware graph.

1.6.1 `OptimizeStep` assignment

If no `OptimizeStep` is found in the procedure and the optimization is not running in the interactive mode, all steps that may be subject for the optimization will be automatically wrapped with `OptimizeStep` and the corresponding parameter will be set within the range of ±20% from the initial value. The full list of the available steps is given in the Appendix A.

1.6.2 `Analyze` step assignment

If no `Analyze` step is found in the procedure and the optimization is not running in the interactive mode, an `Analyze` step with `method=”interactive”` will be inserted in the procedure after the step that presumably indicate the reaction completion. Such steps are: `Stir`, `Wait`, `HeatChill`, `HeatChillToTemp`. To avoid any confusion and incorrect reaction analysis, it is recommended to insert the `Analyze` step manually in the procedure. If no `Analyze` step is found and the optimization is not running in interactive mode – an exception will be raised.

1.6.3 Callback assignment

Depending on the given `Analyze` step’s method, the callback function will be assigned to correctly calculate and update the result of the reaction. Examples of such functions are listed below, based on the optimization target:

- `final_parameter` – a generic target for running optimization in interactive mode, no calculations will be performed on the user input.
- `spectrum_peak-area_XXX` – reaction outcome will be calculated as area under the curve for the given peak (where *XXX* is the *x* axis coordinate) on the spectrum; if no peak at this position was found, the output will be 0. If `reference` is given in the optimization configuration, the resulting area will be divided by the area for the reference peak.
- `spectrum_integration-area_LLL..RRR` – reaction outcome will be calculated as area under the curve within the given *x* axis limits (where *LLL* is the left and *RRR* is the right borders respectively). This method is insensitive to the peaks found. If `reference` is given in the optimization configuration, the resulting area will be divided by the area for the reference peak.
- `novelty` – reaction outcome will be calculated according to the novelty equation (see section 1.8).

1.6.4 Scheduling

If the procedure is executed in a single batch mode, no scheduling is performed. For batched optimization, scheduling the simultaneous execution of several XDL threads in a single run involves establishing an execution plan – i.e., a rough plan of when each step required by the

procedure will be executed – ensuring no resource use overlap between XDL threads. We chose to establish an execution plan rather than, for example, just use resources immediately as they become available to preserve as much as possible any necessary structure and minimize wait times within a given procedure. To establish a schedule, first all XDL scripts and the hardware graph are parsed, and all mappings between each of the steps within a given XDL script and the hardware graph are obtained. In other words, for a `HeatChill` step and a graph containing multiple reactor vessels, a separate XDL object is created where the `HeatChill` step is attributed to each of these reactor vessels. As a result, we obtain – for each XDL script – a set of possible XDL script-to-hardware maps. Next, each script-to-hardware map is used to establish a set of resource allocations (corresponding to a list of times that each graph node is in use by a given XDL thread). With all these resource allocations in hand, this constitutes an optimization problem, where the objective is to find the XDL script-to-hardware map for each XDL script that minimizes the overall execution time, where no resource is allocated to 2 XDL threads simultaneously at any time. In our case, we implemented several optimization algorithms (genetic algorithm, random search), but found that all combinations could be exhaustively searched given the size of our hardware graph.

G: hardware graph.
$\{XDL_i\}$: set of XDL scripts to be executed.

Mapper: Get all XDL – hardware maps.
Allocate: Get graph resource allocation for a given XDL – hardware map.
Product: Generate all combinations of XDL – hardware maps, taking one possible map per XDL file.
Schedule: Returns the minimum time execution schedule for a given set of XDL resource allocations.
Evaluate: Calculates the execution time of a schedule.
S: Schedule of XDL execution.
t: Execution time.

$\{\{M_i[XDL_i]\}\} \leftarrow$ **Mapper**(XDL_i, G)
$\{\{R_i[M_i]\}\} \leftarrow$ **Allocate**($\{\{M_i[XDL_i]\}\}$, G)

for *combination* in **Product**($\{\{R_i[M_i]\}\}$) **do**:
    $S, t \leftarrow$ **Schedule**(*combination*)
    if $t < t_{\min}$ then:
        $t_{\min} \leftarrow t$
        $S_{\min} \leftarrow S$
return $S_{\min}$

Fig. S7. Scheduling algorithm pseudocode. In this case, all schedules are generated and the one with the minimum execution time is selected.

Typically, the expected acceleration when using 2 reactors is less than 2x due to multiple processes relying on the same hardware resources. For example, the 2-fold parallel system had an estimated 1.6X acceleration and a 4-fold parallel system exhibited a 2.7x acceleration in simulations. It is important to note that these factors can vary based on the experiments selected by the algorithm (e.g. different combinations of short and long reactions times allowing for different degrees of concurrent execution). In practice, the acceleration depends on the accuracy of the expected durations for the different XDL steps that are used by the scheduling routine. In our hands, this led to factors of < 1.6X, however this is expected to improve as we collect more data on actual durations.

1.6.5 Hardware mapping and implied steps addition

In addition to the XDL compilation process, further preparations are needed for the `Analyze` step. Some of the implied steps will be added according to the analytical method used, e.g., shimming for the NMR, however others are general depending on the analytical steps parameters:

- **Sampling**. If sampling has to be performed additional steps to transfer the aliquot to the analytical instrument are added to the list of `Analyze` step substeps: transfer an aliquot with an excess volume from the reaction vessel to the injection syringe pump (i.e., the closest pump to the analytical instrument); transfer the aliquot from the injection pump to the analytical instrument; transfer the excess volume from the injection syringe back to the reaction vessel.
- **Dilution**. If reaction sample has to be diluted prior to the analysis the following steps are added to the list of `Analyze` step substeps: the liquid path from the reaction vessel to the dilution vessel is primed with the reaction sample; an aliquot is transferred from the reaction vessel to the dilution vessel; the dilution solvent is then added to the dilution vessel; dilution vessel is stirred for a pre-set time (10 minutes).
- **Cleaning**. If analytical instrument is required to be cleaned after the analysis, the necessary steps to transfer the cleaning solvent to the instrument and flushing the liquid path with gas are appended to the end of the `Analyze` step substeps.
- **Shimming** (only for the NMR analysis). Shimming is triggered when shimming results are not found on the PC or last shimming was performed more than 24 hours before or `force_shimming` attribute was set. If shimming is required, the solvent for shimming is chosen according to predefined rules, transferred to the instrument and used as a reference for shimming routine.

1.7 Spectra analysis

All spectra acquired during the optimization procedure are managed by the `SpectraAnalyzer` class, which features several methods to calculate reaction outcome (examples are listed in section 1.6.3) and analyze several spectra on a time scale. The analysis of the NMR spectra is based on the `SpinsolveNMRSpectrum` class from `AnalyticalLabware` module and `nmrglue` library methods for the analysis. The analysis of the HPLC spectra is based on the `AgilentHPLCChromatogram` class from `AnalyticalLabware` module. The analysis of the Raman spectra is based on the `RamanSpectrum` class from `AnalyticalLabware` module. Calculations for the novelty analysis are described below in the section 1.8.

1.8 Novelty analysis

The novelty analysis is a reaction descriptor used to drive the optimization towards unexplored product space. It is based on the information gain from the reaction spectrum and the spectrum's novelty with respect to previously measured spectra of the same reaction. The analysis begins with search for regions of interest (i.e., containing meaningful information – peaks)[4], calculation of their individual scores, total information score for the current spectrum and additional coefficient for the current spectrum novelty.

1.8.1 Individual regions score

Score for individual region is calculated as the size of the region (as number of data points) multiplied by the inverse logarithm of the difference between area of the current region and harmonic mean of all identified regions (Eq. 1).

$$Rs_i = Size_i \times \frac{1}{\log_{10}(|area_i - \overline{area}|)} \quad (1)$$

If one of the region's area is equal to overall harmonic mean, that this region's score is equals to its size. Harmonic mean is used to favour smaller peaks and reduce the weight of large spikes and potential false positives (i.e., noise identified as material peaks). An example of the individual regions scoring is shown in Fig. S8.

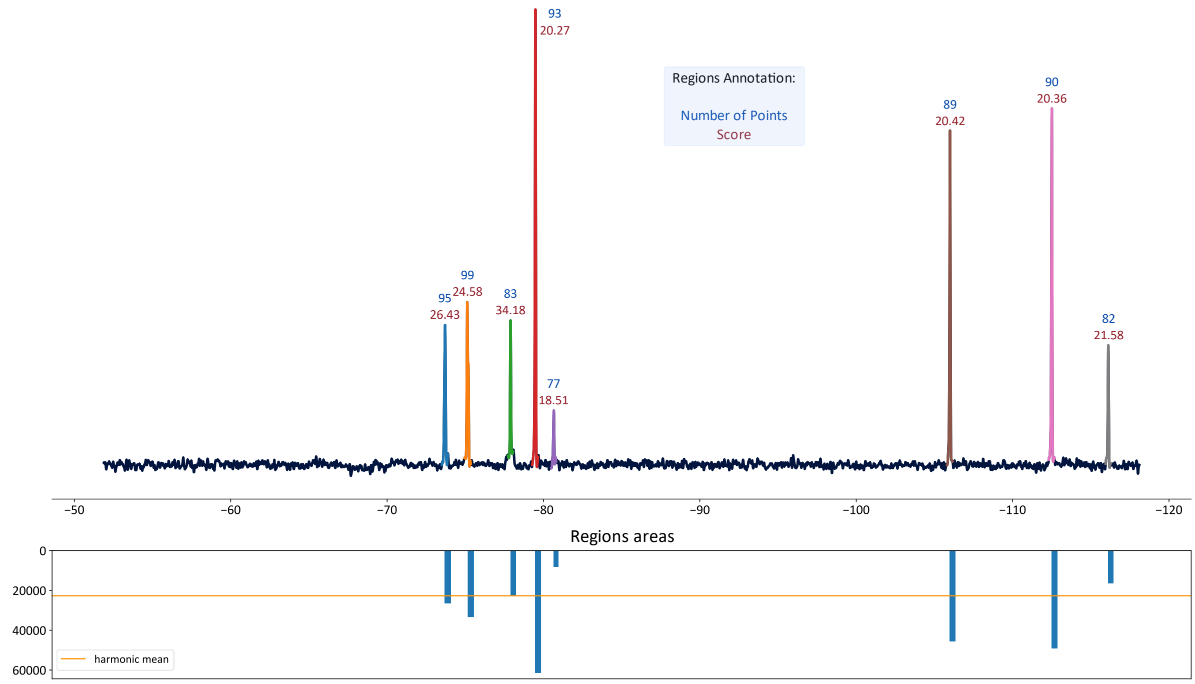

Fig. S8. An example of the individual regions scoring. Top: ^{19}F NMR spectrum with each identified region is highlighted with colour with top annotations indicating peak's size (in number of data points) and score (see Eq. 1). Bottom: bar chart with areas for individual peaks (aligned on the *x* axis) with their harmonic mean indicated as orange horizontal line.

1.8.2 Information score

Total information on a single spectrum is calculated by multiplying the sum of individual regions by the total number of identified regions (Eq. 2).

$$\sum_{i=1}^{r}(Rs_i) \times r \tag{2}$$

The score is insensitive to the spectrum phasing (e.g., for NMR spectrum) and will favour spectra with large amount of equally sized regions (see Fig. S9 for an example).

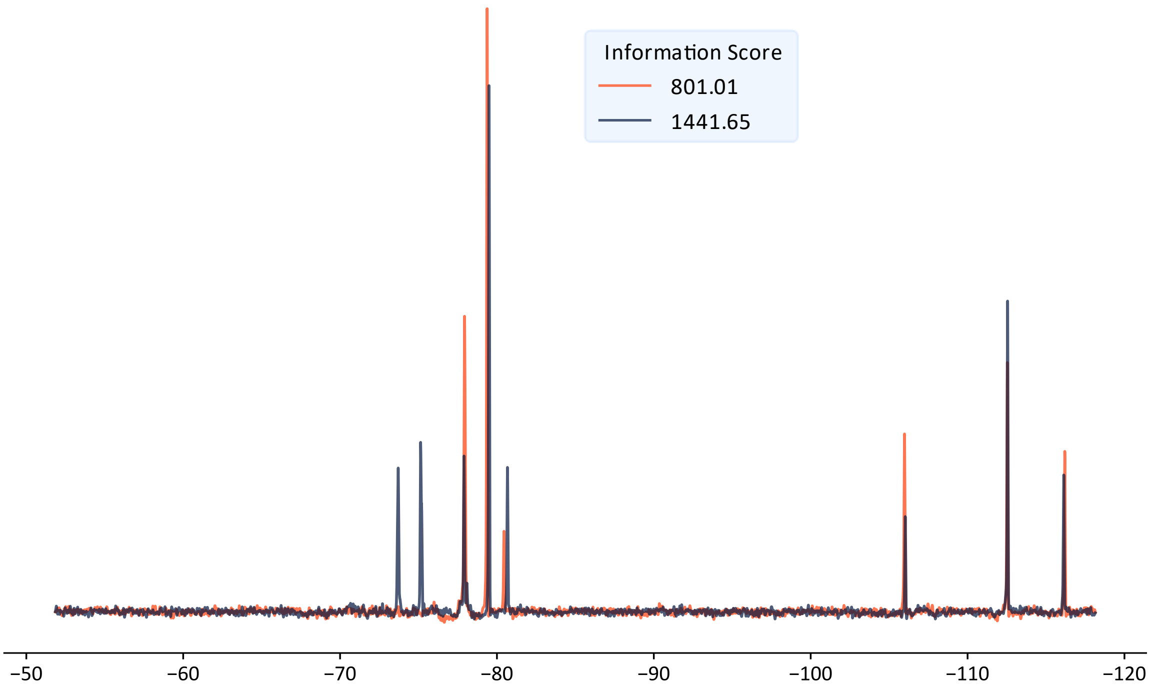

Fig. S9. An example of the information score difference for two ^{19}F NMR spectra.

1.8.3 Novelty coefficient

The novelty coefficient is calculated with respect to all previously measured spectra, for which identified regions are stored as arrays with their x axis coordinates. All identified regions for the spectrum of interest are checked versus previously identified regions (spectrum-wise) and the spectrum with regions not found previously will have a higher coefficient (Eq. 3).

$$Nc_i = \frac{|\mathrm{F}_i \backslash \mathrm{P}|}{|\mathrm{F}_i|} + \frac{1}{|\mathrm{P}|},\ where\ \mathrm{P} = \bigcup_{j=1}^{k \neq i} \mathrm{F}_j \tag{3}$$

$$\mathrm{F}_i - \text{set of identified regions for } i^{\text{th}} \text{ spectrum}$$

The novelty coefficient is insensitive to the regions height and is reduced with the number of obtained data. An example of the novelty coefficient is illustrated on Fig. S10.

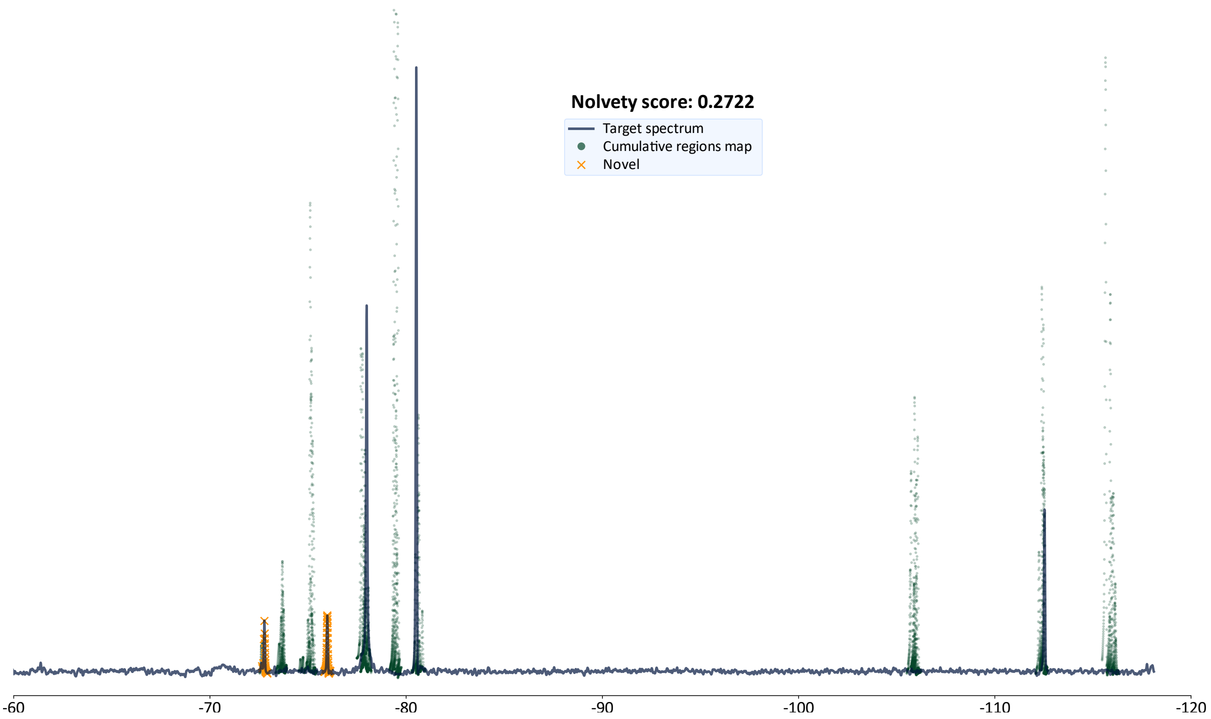

Fig. S10. An illustration of the novelty coefficient calculation. The spectrum of interested is plotted navy grey, set of individual regions for all previously measured spectrum is plotted as green circles (labelled as "Cumulative regions map") and novel regions are highlighted with orange crosses.

1.9 Algorithm interface

The `AlgorithmAPI` class is designed to provide a unified interface to the algorithmic classes and dynamic XDL execution. The class contains methods to parse the reaction parameters into parameter-agnostic data arrays suitable for the numeric optimization algorithms, initialize algorithm classes, query data for the next setup and save the intermediate results.

1.9.1 Available algorithms

1.9.1.1 Random search

Random search algorithm relies on the `random` submodule of the NumPy library.[5] The suggestion of this algorithm is a random number array (sampled from a uniform distribution) within the parameters constraints. The following parameters are available to construct the algorithm's class:

- `random_state` – a seed to initialize low-level random number generator, used to preserve reproducibility across multiple optimization runs.

1.9.1.2 Genetic algorithm

A basic genetic algorithm was written and adopted for the sequential optimization of chemical reactions. It uses truncation selection, single point crossover, and random reset mutation while preserving the best solution (elitism). In case of premature convergence, the population is reinitialized. The key hyperparameters to tune are:

- `pop_size` – Number of individuals in the population.
- `mutation_rate` – Probability (0 to 1) of mutating a gene.

1.9.1.3 Sequential model-based optimization (SMBO)

The algorithm implementation is based on the `scikit-optimize` python library and provides a simple and efficient library to minimize expensive and noisy black-box function.[6] The parameters for the underlying algorithm are listed in the official documentation.[7]

1.9.1.4 Design of Experiments

Several experimental designs are available via a wrapper for `pyDOE2`[8]. Available designs include full and fractional factorial designs, Response-surface designs, and randomized designs. The wrapper calls the appropriate `pyDOE2` functions to generate the design matrix and maps the levels onto the appropriate parameter values based on the search space bounds.

1.9.1.5 Dummy methods

The following "dummy" methods are given to the user to run the given reaction iteratively using the respective setup on each iteration:

- `fromcsv` – the next setup is read from the given csv file.
- `reproduce` – the next setup is equal to the initial setup from the procedure. Used to check the experimental outcome for reproducibility.

1.9.2 Optimizer Client

The `OptimizerClient` represents the class for communication with the `SummitServer` (see section 1.10). The communication is based on the TCP-IP sockets and managed via `selectors` and `socket` modules of the python standard library.[9] The communication messages are encoded json-like dictionaries containing information about the current procedure, steps and parameters subjected to the optimization and optimization configuration.

The class is initialized if the requested algorithm is the one available in the Summit framework and managed via `AlgorithmAPI`.

1.10 Summit Server

The `SummitServer` is the python module that provide access to the Summit framework via TCP-IP communication. The module can be set on a remote server and supports connection for multiple clients with python `selectors` library. The format of the communication messages is JSON with all reaction parameters encoded as "<step name>_<step ID>-<parameter name>", all parameters for the algorithm and a "hash" field, that allows matching same optimization procedure running on various clients.

1.10.1 OlympusWrapper

`OlympusWrapper` is a class that serves as an optimization handler within the `ChemputerOptimizer` server framework, providing access to the state-of-the-art algorithms implemented in the Olympus[10] benchmarking framework. It parses the JSON requests from the client and constructs the appropriate parameter space, observation and planner objects from Olympus. Queried points are returned in JSON format for immediate execution on the ChemPU platform. To use specific algorithms, the user must install the Olympus package and all required dependencies by the algorithm.

1.11 Data management

At the end of every iteration, the following data is saved:

- Executed procedure (as `.xdl` file) – contains the procedure executed at the current iteration with corresponding parameters; could be for the future reproduction.
- Parameters used (as `.json` file) – stand-alone file stating all the parameters optimized for the current procedure, their values at the current iteration, and the description and the value for the target for the current iteration.
- Results list (as `.csv` file) – stand-alone file, stating all reaction parameters and results used so far for the current procedure. This file is used to set up the algorithm to continue the optimization.
- Parallel schedule (as `.json` file) – stand-alone file, stating the schedule of the procedure steps if the optimization is running in parallel using several reaction modules.
- Spectrum (as `.pickle` file, see section 2) – the spectrum obtained during the FinalAnalysis or Analysis step executed at the current batch/iteration (if applicable).

The data is managed using the NoSQL document database. The entry files are generated using the stand-alone script from the files listed above.

1.11.1 Database schema

An example XDL Database entry schema is shown in Scheme S2 and S3. The base table 'Experiments' gathers the key information and meta data of the experiment. The meaning of the individual entries in the base table (**Scheme S1**) is defined in **Table S1**.

```
Experiment Table
Experiments(
    ID varchar(255) UNIQUE NOT NULL PRIMARY KEY,
    Username varchar(255) NOT NULL,
    ExperimentCode varchar(255),
    Public boolean,
    Description text,
    SMILE text,
    INCHI text,
    Validated boolean NOT NULL,
    Success boolean,
    Keywords text,
    ExperimentSeries varchar(255),
    Iteration integer,
    ExactRepeat Boolean,
    ExactRepeatOfExperiment varchar(255),
    EstimatedDuration real,
    ExperimentDuration real,
    ReactionClass varchar(255) NOT NULL,
    Scale real,
    Datetime timestamp,
    Location varchar(255)
)
```

Scheme S1. Basic schema of XDL top-level database entry defining the entry ID and capturing all relevant meta data

Table S1. Explanation of the entries of the basic table.

Entry	Comment
ID	Unique identifier
Username	Owner of the entry
Experiment code	Laboratory code (reference to lab notebook) of the experiment
Public	Defines whether the entry is public or private

Description	A short description of what is important about this reaction
SMILE	SMILE of the reaction
INCHI	INCHI of the target product
Success	Defines whether the outcome of the reaction was successful or not
Keywords	Keywords that are characteristic for the reaction (e.g. 'metathesis')
Experiment series	An identifier for all runs that have been performed for this reaction
Iteration	The iteration of this experiment in the experiment series (e.g. the third attempt on a reaction with modified conditions would be iteration 3)
Exact repeat	Number of the exact repeat of an experiment
Exact repeat of experiment	ID of the experiment entry that has been repeated exactly
Estimated duration	The estimated run time of the experiment
Reaction class	The reaction class the experiment belongs to. The available reaction classes are listed in **Table S2**
Scale	The amount of the limiting reagent in mol or mmol
Datetime	Starting date of the experiment
Location	Address of the lab in which the experiment was performed

Table S2. List of used reaction classes.

**Entry**	**Reaction class**
1	C-C bond formation (TM catalysed)
2	C-C bond formation (TM non-catalysed)
3	Heteroatom alkylation and arylation
4	Functional group manipulation
5	Protecting group manipulation
6	Ring and heterocycle formation
7	Reductions and oxidation
8	Multicomponent reactions
9	Other

The 'XDLEntry' table gathers the information associated with the experimental procedure and execution. The meaning of the entries in this table (Scheme S2) is defined in Table S3

```
XDLEntry (
    ExperimentID varchar(255) REFERENCES Experiments (ID),
    XDLVersion real NOT NULL,
    XDL text NOT NULL,
    XDLEXE text NOT NULL,
    Graph text NOT NULL,
    SimulationLog text,
    ExecutionLog text,
    ExecutionScript text,
    LiteratureProcedure text
)
```

Scheme S2. The XDLEntry schema captures all entries that are directly or indirectly relevant for the actual procedure and links it to the experiment ID defined in the top-level schema.

Table S3. Explanation of the entries of the XDLEntry table.

Entry	Comment
Experiment ID	ID of the database entry as defined in the base table (Scheme S1)
XDL version	Version of the XDL code the experiment has been run with
XDL	The XDL file for the experiment
XDLEXE	The XDLEXE file for the experiment
Graph	The graph file for the experiment
Simulation log	The folder containing the simulation logs of the experiment
Execution log	The folder containing the execution logs of the experiment
Execution script	The Python script used to compile, simulate, and execute the XDL
Literature procedure	The prose procedure text for the experiment (if available)

All entries that are not present in the files generated at the end of iteration execution are filled manually.

2 Analytical Labware

The `AnalyticalLabware` python module contains classes and method to control various analytical instruments and process obtained spectra in a unified way. A generic class for data processing (`AbstractSpectrum`) provides abstract method for loading the spectral data – redefined in the ancestor classes accordingly – and methods for loading and saving data as a binary key-value storage (where keys are relevant spectral data, e.g., x and y axes data,

timestamp, peaks, etc.) for subsequent use within `AnalyticalLabware` framework. Common methods for processing and analysis include baseline correction[11] peak picking, integration and spectrum smoothing, with support methods for trimming and displaying the spectrum.

2.1 Magritek Spinsolve NMR

The `devices` submodule contains classes to control the Spinsolve NMR and process obtained 1D NMR spectra: `SpinsolveNMR` and `SpinsolveNMRSpectrum` respectively. The spectrometer control is achieved via client-based communication with the Spinsolve software. SpinsolveNMR class provides interface to retrieve available protocols and their options, execute those and store the data within `SpinsolveNMRSpectrum` class.

The `SpinsolveNMRSpectrum` inherits from `AbstractSpectrum` and additionally uses methods from `nmrglue` library[12] for NMR spectra processing: Fast Fourier transformation (FFT), baseline correction, zero-filling and apodization. In addition, the class features the methods for region analysis.

2.2 Agilent 1260 Infinity II LC

The `devices` submodule contains classes to control the Agilent 1260 LC and process obtained data: `HPLCController` and `AgilentHPLCChromatogram` respectively.

Communication with the instrument is established via a Chemstation macro `hplctalk.mac` that monitors and executes commands written to a command file and returns replies by writing them to a reply file. After downloading the macro from `AnalyticalLabware`, it is placed in the Chemstation macro directory. This macro must be loaded and activated in the Chemstation software using the following commands:

```
> Macro hplctalk.mac
> hplctalk_run
```

On the python side, the following methods are available: turning on and off the pump and lamp, switching the chromatographic method, preparation and start of a run, checking the instrument status and switching to standby mode. These methods are implemented to write specific commands to the command file and parse the responses received.

The `AgilentChromatogram` class is inherited from `AbstractSpectrum` and has additional methods for loading the raw data from the propriety Chemstation file format.

2.3 Ocean Insight QE Pro Raman spectrometer

The `devices` submodule contains classes to control the Ocean Insight QE Pro Raman spectrometer and process obtained data: `OceanOpticsRaman` and `RamanSpectrum` respectively. The spectrometer class has methods implemented to obtain a (blank) spectrum and control over integration time and number of scans performed.

The `RamanSpectrum` class is inherited from `AbstractSpectrum` and has two additional methods useful for processing of the Raman spectra: subtracting the reference and iterative peak picking algorithm with sliding threshold, which shows better performance in noisy spectra.

3 ChemPU process monitoring

The enhanced ChemPU features a sensor package to monitor the status of the platform during a run as well as to use real-time data to make decisions for dynamic procedure execution on the fly.

3.1 SensorHub

For integration of process sensors into existing ChemPU stack, we have designed an Arduino shield with built-in Ethernet module for control over an IP network. The board features 12 MOSFET outputs, ethernet-to-serial converter and 8 individually controllable I^2C channels on top of the functionality given by the Arduino board[13]. Overall, it allows installation of up to 8 analogue sensors, 8 sensors with I^2C communication (achieved using an I^2C multiplexer, thus allowing installation of devices with same addresses), several sensors with SPI communication (achieved via “software SPI” Arduino feature) and up to 12 PWM devices (Fig. S11).

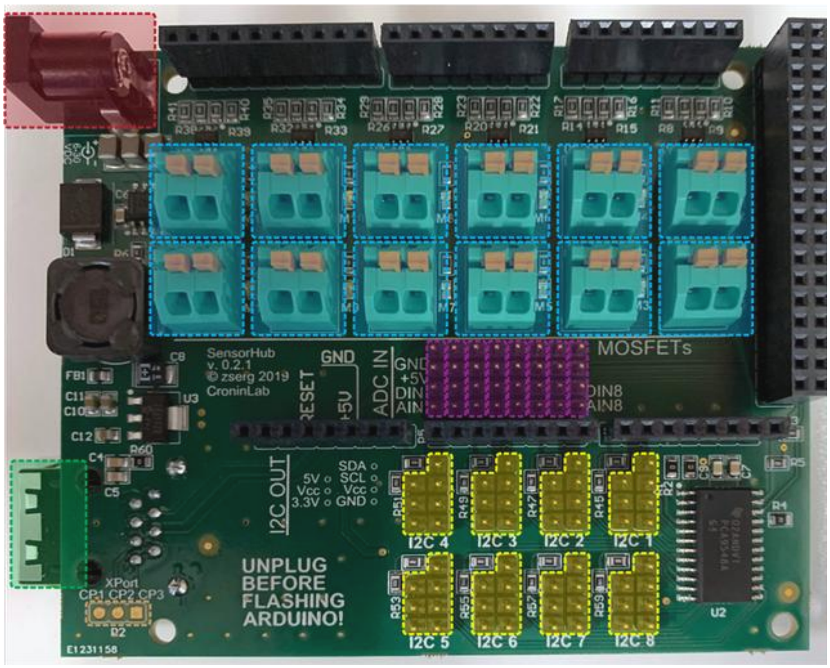

Fig. S11. SensorHub board.

3.2 Process sensors

Individual sensors were selected from commercial manufacturers providing details installation manual and a corresponding software library for seamless integration. Sections below provide detailed descriptions of each individual sensors used in the ChemPU process monitoring framework.

3.2.1 Colour (RGBC) sensor

The TCS34725 Colour Light-to-Digital Converter with IR Filter[14] was selected as the colour sensor for process monitoring. The main component of the converter is 3 × 4 photodiode array, composed of the red-filtered, green-filtered, blue-filtered, and unfiltered (clear) photodiodes, additionally coated with an IR-blocking filter. The four integrating analogue-to-digital converters (ADCs) simultaneously convert the amplified photodiodes currents to a 16-bit digital value, which is sent over the I^2C communication bus. We have chosen a DFRobot board (manufacturers part No. SEN0212) as an evaluation board for the sensor, featuring a unified PH2.0-4P interface for connection to the SensorHub via a crimped cable and a white LED as a light source. The sensor is attached to a round bottom flask (Fig. S12).

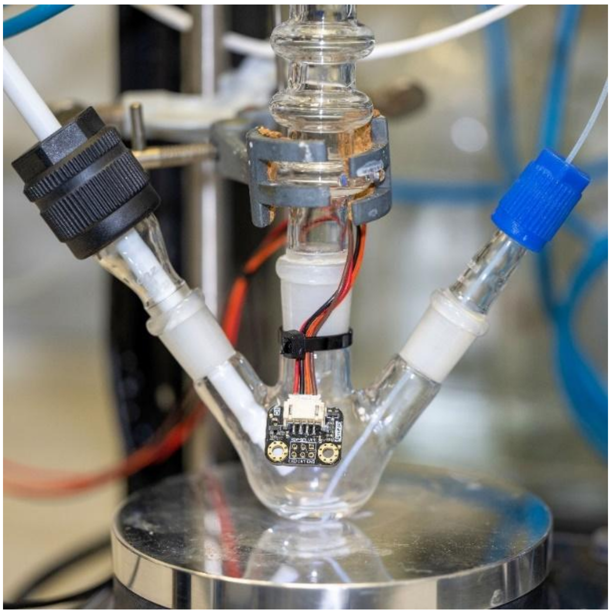

Fig. S12. An example of the Colour sensor installation.

3.2.2 Environmental conditions sensor

To BME280 digital humidity, pressure, and temperature sensor[15] was selected for the environmental conditions monitoring. We have chosen DFRobot board (manufacturer part No. SEN0236) as an evaluation board for the sensor, featuring a unified PH2.0-4P connection interface for I^2C communication. The sensor board is placed inside the fume hood (Fig. S13).

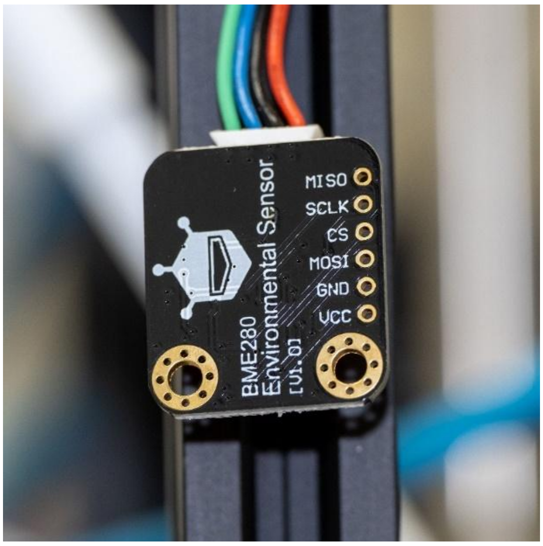

Fig. S13. An example of the environmental sensor setup.

3.2.3 Liquid sensor

The OPB350 Tube Liquid Sensor[16] was selected to monitor the liquid handling system of the ChemPU. The sensor consists of an IR LED, phototransistor and tube housing, and is designed

to recognize the presence of the fluid inside the tube. A custom PCB was designed to simplify the operation and the connection of the sensor to the SensorHub (achieved using analogue input interface). The wired assembly sensor (manufacturer part No. OPB350W125Z for 1/8” tubing and OPB350W062Z for 1/16” tubing) are connected to the board using screwless wire terminal and installed on the tubing connecting syringe pump with the closest switch valve (Fig. S14).

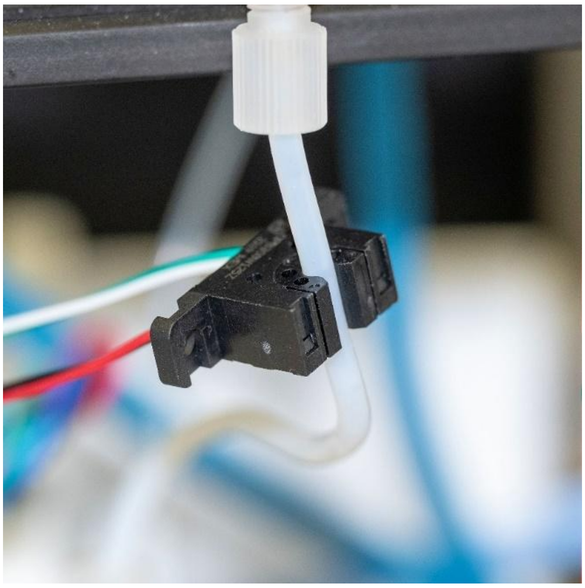

Fig. S14. An example of the liquid sensor installation.

3.2.4 Reaction temperature sensor (RTD)

The MAX31865 RTD-to-Digital Converter[17] was selected to simplify the interfacing the SensorHub with resistance temperature detector (RTD, or temperature probe). The unit has a built-in 15-bit ADC, input protection, a digital controller, and an SPI-compatible interface. We have selected Adafruit PT100 RTD Temperature Sensor Amplifier (manufacturer part No. 3328) as an evaluation board for the MAX31865 converter and 4-wire PTFE-encapsulated PT100 temperature probe (BOLA, part No. P1750-15) installed via swivelling screw fitting (BOLA, part No. D692-24 for GL18 glass thread) on the round bottom flask. An installation example is shown below (Fig. S15).

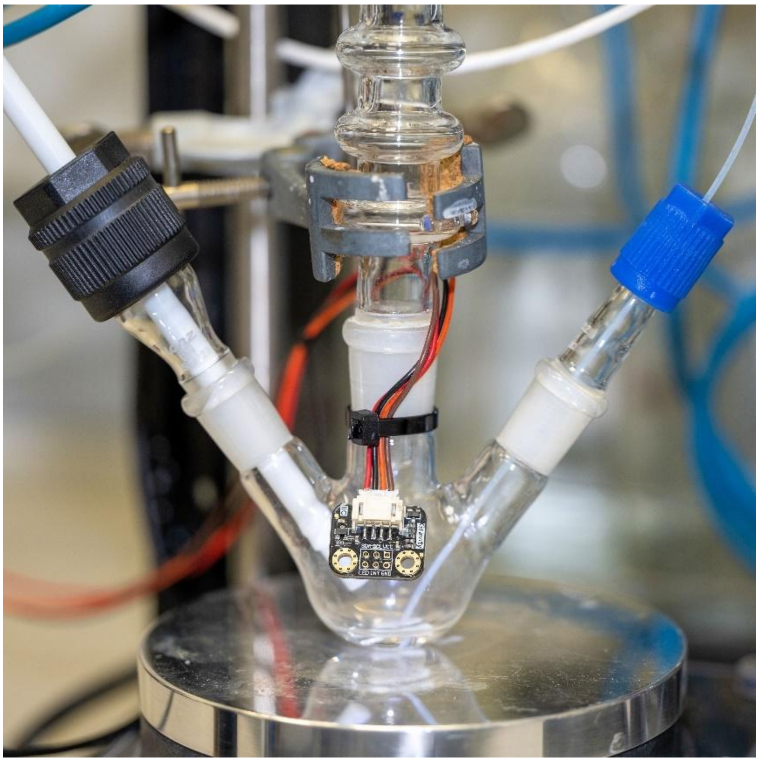

Fig. S15. An example installation of the temperature probe (RTD, left).

3.2.5 IP Camera

For video recording and computer vision applications, we have selected the UniFi Video Camera G3 Dome (UCV-G3-DOME) which features a wide-angle lens and 1080p high resolution video performance. Following the general ChemPU hardware design principle, the device is compatible with the PoE standard and has an assigned IP address. For a miniaturized ChemPU rig a single camera is sufficient while for a standard ChemPU rig one camera is used to monitor the liquid handling backbone while a second camera monitors the various modules. Passive video monitoring as well as CV-based anomaly detection (details given in Section 3.4) are available via the `start_recording` method of the `Chempiler` class.

3.3 Software architecture

The low-level sensors control is implemented via Arduino CommandTools framework, which provides a unified interface for communication with various devices via Arduino boards[18]. The corresponding python library – `Commanduino` – provides python interface for communication and control of any device interfaced with Arduino `CommandTools`[19]. The sensors package is integrated into `Chemputer` stack via `ChemputerAPI` and `Chempiler` libraries, which provides high-level individual sensor access and overall control of the background recording thread and data i/o respectively.

To provide access to sensors control and execution for the end user through XDL framework the following steps were developed:

- `SetSensor` – the starting point to control the background sensor monitoring thread. A callback function could be assigned during compilation, that will be executed with timestamp argument when the sensor will be set.
- `ReadSensor` – the step to take a single reading from the sensor and pass it to the callback function (if assigned during the compilation). The reading is acquired from the background monitoring thread, or, if the latter was not started, by direct communication with sensor.
- `GetSensorReadings` – the step to take several last readings, filter them (if required) and pass to the callback function (if assigned during the compilation). The readings are requested either by the timestamp or by the number of readings. The following filtering functions are available: median filter and FFT filter.

3.4 Computer vision

Vision-based condition monitoring system includes two functional modules supporting the detection of syringe, which is the pivotal element of the pump, and its breakage: based on experience of a ChemPU user, the bottom of the syringe dismantles from the rest of the body leading to leakage of the chemical in use.

For syringe detection, multi-scale template matching is used to determine the best match between template and target images. Since the cameras are at fixed positions and Syringe's appearance remain the same under laboratory conditions, multi-scale template matching is opted for its simplicity and speed to support active condition monitoring of the ChemPU in real-time. For this purpose, template for each syringe is manually created and using sliding window technique to match templates on the target image. Match with the highest score is chosen as the best match. To mitigate the variation in illumination leading to false positives, each image is normalized to zero mean; the range of pixel intensity values change to remove noise from the image. In addition, a check for mean intensity greater than a predefined threshold for each incoming image is performed to avoid false detection in the event of ChemPU lighting failure. This template-based approach could further be extended to other

modules (valves, vessels etc.), enabling safe, autonomous execution of increasingly complex workflows.

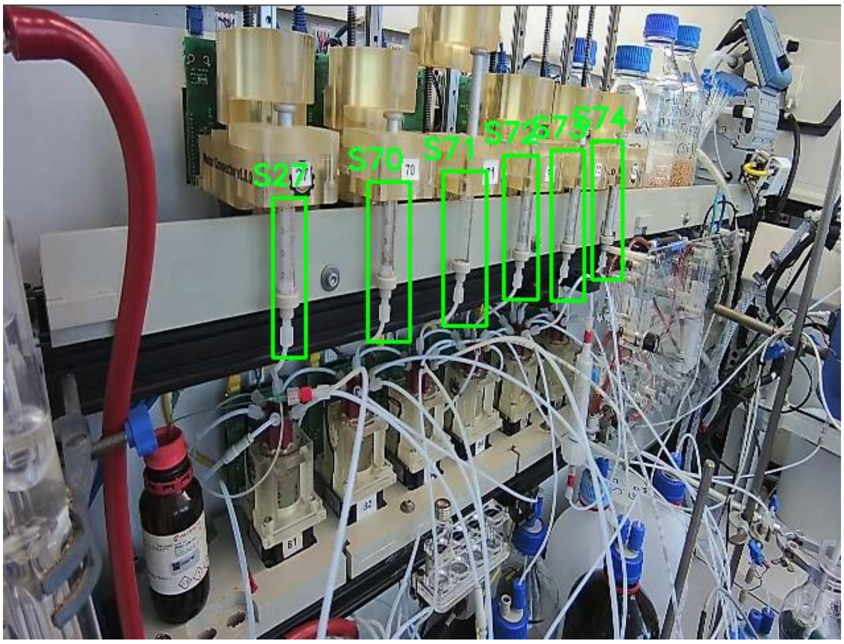

Fig. S16. Illustration of all syringe instances detected using multi-scale template matching. The bounding boxes are extended from the matched template to cover entire syringe for visualization.

Once all instances of syringes are detected, the respective template region for two images a second apart are monitored for anomaly by finding the difference in the images. In contrast to a mean squared error of the pixel values by comparing each pixel individually and independently for two images, we take a holistic approach of structure similarity inspired by human images perception. For detection of a syringe breakage during ChemPU operations, a structure similarity index measure (SSIM)[20] is used as a measure of difference between images a second apart. If a change higher than a predefined threshold i.e., 0.95 - 0 for mutually exclusive images and 1 for similar images - we deduce occurrence of a syringe breakage event.

The SSIM formula is based on three comparison measurements between the images of x and y: luminance (l), contrast (c) and structure (s), where μ_i, σ_i^2 are the average and the variance for the image i; σ_{xy} is the covariance between imagex x and y; c_j is the variables to stabilize the divisions with weak denominator, L is the dynamic range of the pixel value (typically $2^{\#bits\ per\ pixel}$ - 1), $k_1 = 0.01, k_2 = 0.03$ by default.

$$l(x,y) = \frac{2\mu_x\mu_y + c_1}{\mu_x^2 + \mu_y^2 + c_1}, where\ c_1 = (k_1L)^2$$

$$c(x,y) = \frac{2\sigma_x\sigma_y + c_2}{\sigma_x^2 + \sigma_y^2 + c_2}, where\ c_2 = (k_2L)^2$$

$$s(x,y) = \frac{\sigma_{xy} + c_3}{\sigma_x\sigma_y + c_3}, where\ c_3 = c_2/2$$

$$\text{SSIM}\ (x,y) = \left[l(x,y)^\alpha \cdot c(x,y)^\beta \cdot s(x,y)^\gamma\right]$$

Setting the weights α, β, γ to 1:

$$\text{SSIM}\ (x,y) = \frac{(2\mu_x\mu_y + c_1)(2\sigma_{xy} + c_2)}{(\mu_x^2 + \mu_y^2 + c_1)(\sigma_x^2 + \sigma_y^2 + c_2)}$$

3.5 Dashboard

For real-time process monitoring we have developed an interactive web-based dashboard application using Python Dash library[21]. The dashboard class is initialized before the synthesis execution, connects to sensors found on the current graph and ties the control of those with an application callback functions (see sample code below, Fig. S17). The graphical interface features simple control options over available sensors (Start/Stop and reading frequency change) and an interactive plot with real-time data. An example screenshot of the dashboard operation is shown below (Fig. S18).

```
# ChemPU library import
from chempiler import Chempiler
import ChemputerAPI

# Dashboard library import
from chempu_dashboard import Dashboard

# Initializing chempiler object
c = Chempiler(
    experiment_code='Experiment Name',
    graph_file='graph.json',
    output_dir='Output Directory',
    simulation=False,
    device_modules=[ChemputerAPI]
)

# Initializing dashboard
dashboard = Dashboard(c)

# Starting dashboard server
```

```
# Graphical interface is available at localhost:8050
dashboard.run()
```

Fig. S17. An example code snippet to run the dashboard for real-time process sensors control and monitoring.

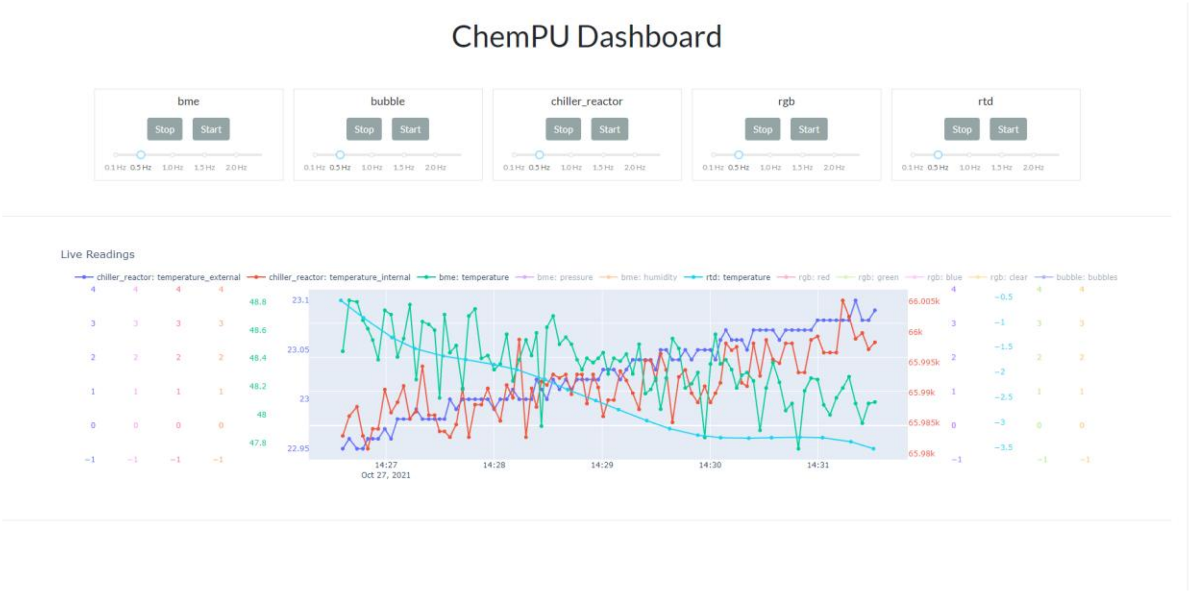

Fig. S18. An example screenshot of running interactive dashboard for process sensors monitoring.

4 XDL Dynamic Step

As introduced previously – `AbstractDynamicClass` is an abstract base class for all steps that will be executed dynamically using the feedback from the hardware modules (e.g., process sensors or analytical instruments).[3] Here we introduce 3 subclasses: `DynamicAdd` – for dynamic reagent addition followed by the feedback from the process sensors; `DynamicTransfer` – for dynamic liquid transfer through pump-valve backbone system with passive feedback from tube liquid sensor; `DoUntill`– for dynamic reaction execution (i.e., stirring or heating) with active feedback from the process sensors. The dynamic step execution is achieved through the use of `on_start`, `on_continue` and `on_finish` method, which each return a list of steps to be executed based on the current state of the step.

4.1 Dynamic Addition

The `DynamicAdd` step is designed to control the reagent addition by the feedback of the process sensor installed on the target vessel. Examples of process sensors include temperature (for exothermic or endothermic reactions), RGB (for reaction involving colour change during the reagent addition) or any other sensor which can be controlled using the XDL framework. The following substeps are executed during `DynamicAdd` execution:

- `on_start` method returns initial set of substeps: priming pump for addition, starting stirring (if required) and starting the background monitoring thread for the target sensor.
- `on_continue` method returns the following steps based on the current state of the execution: 1) filling the syringe (if the volume transferred is less than target volume), 2) adding the aliquot to the target vessel (if the sensor reading is within the safe margin from the threshold) or waiting for settling time (if the sensor reading is outside the safe margin limits), 3) obtaining new reading from the sensor.
- `on_finish` method returns the steps in analogy to the `Add` XDL step: flushing the tubing, stopping the stirring (if required) and stopping the background monitoring thread for the target sensor.

The `DynamicStep` step shares common arguments with XDL `Add` step (e.g., `reagent` and `volume`) with addition of several arguments respective to the sensor used and execution control. An example of the step is shown below (Fig. S19).

```
<AddDynamic
  vessel="reactor"
  reagent="30 % hydrogen peroxide solution"
  volume="19.8 mL"
  sensor="rtd"
  reading="temperature"
  reading_threshold="40.0"
  aliquot_volume="5.0 mL"
  wait_after_addition="10"
  settle_delay="20"
/>
```

Fig. S19. An example of dynamic addition of hydrogen peroxide solution to the reactor vessel with temperature (RTD probe) sensor feedback. The following arguments are introduced for sensor and execution control: `reading_threshold` – sensor reading threshold (degrees Celsius in this example); `aliquot_volume` – an aliquot for a single addition before the next reading will be acquired; `wait_after_addition` – delay between addition and sensor reading; `settle_delay` – delay between several acquisitions if the reading is close to the threshold.

4.2 Dynamic Transfer

The `DynamicTransfer` step is designed to monitor the liquid transferring process using tube liquid sensor. In the current implementation this step is for passive monitoring and no decisions are made during its dynamic execution. However preliminary results have shown that it is possible to incorporate the data from the sensor into the workflow and correct the liquid transferring process, when potential errors are encountered, e.g.: blocked tube due to solid

precipitation, insufficient reagent, blocked filter frit during filtration process, etc. The following substeps are executed during `DynamicTransfer` execution:

- `on_start` method returns substeps in analogy to `Transfer` XDL step: stop stirring.
- `on_continue` method returns the following steps based on the current state of the execution: 1) start background monitoring thread for the liquid sensor and increase the reading frequency to 10 Hz, 2) move calculated volume (based on the target volume, transferred volume and maximum syringe volume) to the aspiration syringe pump, i.e., closest to the starting vessel for this liquid transferring step, 3) calculate the amount of liquid transferred and report the calculation, 4) dispense the calculated volume to the target vessel and set the sensor reading frequency to 0.5 Hz, 5) calculate the new volume to transfer.
- `on_finish` method returns the steps in analogy to the `Transfer` XDL step: flushing the tubing, rinsing (if required) and stopping the background monitoring thread for the liquid sensor.

The `DynamicTransfer` step shares attributes with Transfer XDL step, and all parameters for the sensor control are constant values. An example of the `DynamicTransfer` step is shown below (Fig. S20).

```
<DynamicTransfer
  from_vessel="reactor"
  to_vessel="waste_H"
  volume="13 mL"
  rinsing_solvent="water"
  rinsing_volume="1 mL"
/>
```

Fig. S20. An example of dynamic transfer 13 ml from reactor to waste, rinsing with 1 ml of water upon completion.

4.3 Dynamic Execution

The `DoUntil` step is designed to allow for dynamic reaction execution (i.e., steps where reaction takes place, stirring or heating) with the feedback from the process sensor. An example for such process sensor is an RGB sensor for the reaction with colour extensive colour change. The step is used as wrapper around the corresponding reaction step and the following substeps are executed during `DoUntil` execution:

- `on_start` method returns all substeps from the child step until the `Wait` step is encountered and additionally a step to start the sensor in the background thread.
- `on_continue` method acquires and analyses readings from the sensor, and returns Wait step if reaction is not finished or no steps if reaction is complete.
- `on_finish` method returns the remaining substeps from the child step.

An example of the DoUntil step is shown below (Fig. S21).

```
<DoUntil sensor="rgbc" threshold="-3000" reading="clear">
  <Stir
    vessel="reactor"
    time="30 mins" />
```

Fig. S21. An example of the dynamic reaction execution (represented as `Stir` step) guided by the feedback from the RGB sensor. The following attributes are used to indicate the sensor parameters: threshold – threshold value indicating that the process is over, reading – the name of the reading to monitor (in this example it indicates the clear channel of the colour sensor).

4.3.1 Endpoint detection methods

Current implementation of the `DoUntil` step utilizes two possible methods to detect the process endpoint (i.e., the reaction completion):

1. Comparison of the current sensor reading (optionally averaged within indicated time frame) with pre-set threshold value. The threshold value may also be set during the procedure execution, using the specialized XDL step `TakeBackgroundReading`. In a typical use case, such step should be added before the addition of the last reagent or reactant.
2. Comparison of the current sensor reading with previously obtained to detect no change within the indicated time frame. The algorithm is as follows: 1) apply a median filter to the raw sensor readings (within interval *M*); 2) calculate the average value for the interval = *N*; 3) calculate the gradient for the several last averages (i.e., *N*-1, *N*-2, …); 4) detect the end point, where the *K* last points of the gradient lay within *J*% of the current absolute reading. Parameters *M*, *N*, *K* and *J* can be set as the `DoUntil` step properties. An illustrated example of such endpoint detection is shown in Fig. S22.

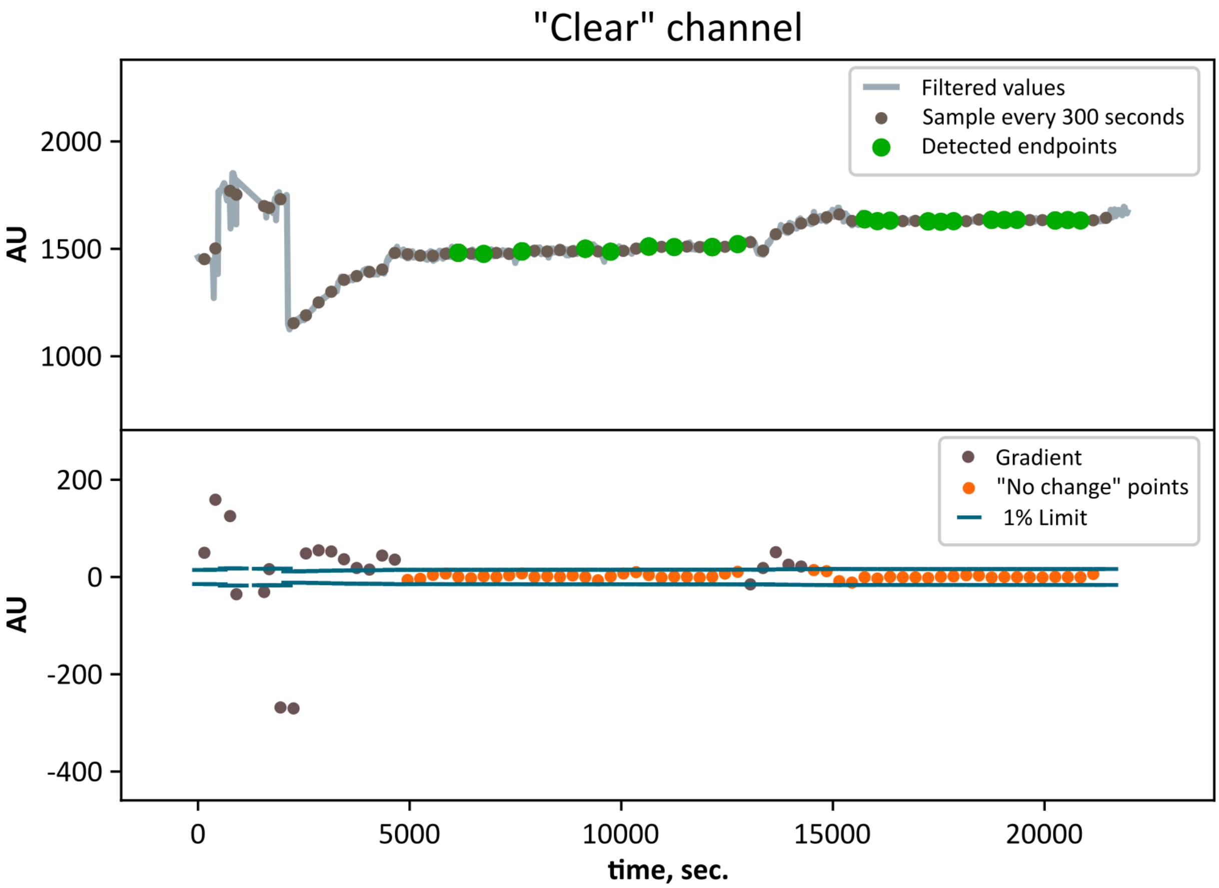

Fig. S22. An example of the endpoint detection method. Top: values obtained from the "clear" channel of the RGB sensor (filtered using median filter), with brown points showing values averaged over 300 seconds interval and green points indicating the detected endpoint time. Bottom: the gradient values calculated from the sampled values, with aquamarine horizontal lines indicating the ±1% value of the absolute sensor reading and "no change" orange points falling within this interval.

5 Experimental section

5.1 General experimental remarks

Solvents and reagents were used as received from commercial suppliers unless otherwise stated. All NMR measurements were recorded on Bruker Avance III HD 600 spectrometer operating at 600 and 151 MHz for 1H and 13C, respectively, or Bruker Avance III 400 spectrometer operating at 400 and 100 MHz for 1H and 13C respectively. Spectra were collected at 298 K, chemical shifts are reported in ppm and were calibrated for the (residual) NMR solvent signal (apparent multiplicities are given as s: singlet, d: doublet, t: triplet, q: quartet, m: multiplet, with coupling constants reported in Hz). The spectra were processed with MestReNova 14.0.0. High resolution mass spectra were obtained on a Bruker maXis LC/MS. For reaction optimization Magritek Spinsolve 80 Carbon (80 MHz operating frequency for ^{1}H) benchtop NMR, Agilent 1260 Infinity II LC system (with IDEX MX II sample loop valve) and Ocean Insight QE Pro Raman spectrometer were used.

5.2 Python script to load and run experiments

All procedures were executed using interactive python environment. The generic script was used to load the procedure and the hardware graph, upload previous results, and run the experiment (Fig. S23).

```python
# ChemputerOptimizer library import
from chemputeroptimizer import ChemputerOptimizer

# ChemPU libraries import
from chempiler import Chempiler
from AnalyticalLabware.devices import chemputer_devices
import ChemputerAPI

# Initiliazing the optimizer object
co = ChemputerOptimizer(
    procedure='procedure.xdl',
    graph_file='graph.json'
)

# Loading optimization configuration
co.prepare_for_optimization('configuration.json')

# Loading results from previous experiments
co.load_previous_results('previous results.csv')

# Initializing chempiler object
c = Chempiler(
    experiment_code='Experiment Name',
    graph_file='graph.json',
    output_dir='Output Directory',
    simulation=False,
    device_modules=[ChemputerAPI, chemputer_devices]
)

# Run the optimization
```

```
co.optimize(c)
```

Fig. S23. Generic python script to run reaction optimization with `ChemputerOptimizer` module using ChemPU. The following file name conventions are used: `procedure.xdl` – XDL file for the procedure optimization; `graph.json` – hardware graph in json format; `configuration.json` – configuration file containing parameters for optimization and corresponding algorithm; `previous results.csv` – previous results in a common table format.

5.3 Sensor experiments

A series of experiments was performed to investigate potential use cases of the sensor package, including syringe breakage detection, liquid handling tracking, turbidity monitoring and robotic process verification.

5.3.1 Syringe breakage detection

An analysis of the failure modes during the automated syntheses reported in Angelone *et al*.[22] revealed that significant proportion of runs failed for unknown reasons or due to human error (Fig. S23). Beyond that, the most common hardware failures were clogging, syringe pump or valve failures which accounted for 43% of failed runs. While issues with the separation, rotary evaporator, valves and clogging could be solved using improved hardware over time, the syringes remained error-prone. Therefore, the computer vision system was developed and tested to alert users of catastrophic syringe failure.

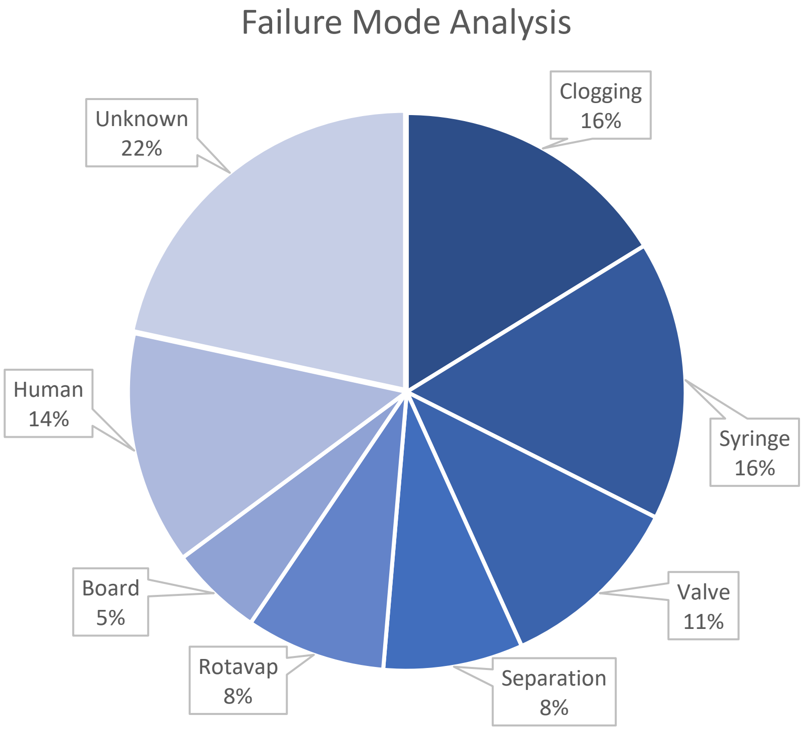

Fig. S24. Failure mode analysis for the Chemputer platform in 2021. For 8 runs the reason for failure was not recorded or remained unclear.

The computer vision system was developed to detect anomalies which are, by definition, rare events. To verify the system, we had to force a syringe breakage. This was achieved by using a 10 mL syringe (ILS, Part No. 2624076-HT) with a loose front fitting and blocking the path to the destination vessel with a plug. As such, liquid could be aspired with the pump, but upon dispensing, the pump motor would stall until the syringe breaks. The system was set up as described in Section 3.4 and water was transferred via the backbone, consisting of two healthy pumps and a pump with the manipulated syringe described above. We could verify that the system worked as expected (Fig. S23). We anticipate to expand on the vision system as we continue to collect data and communicate these developments separately.

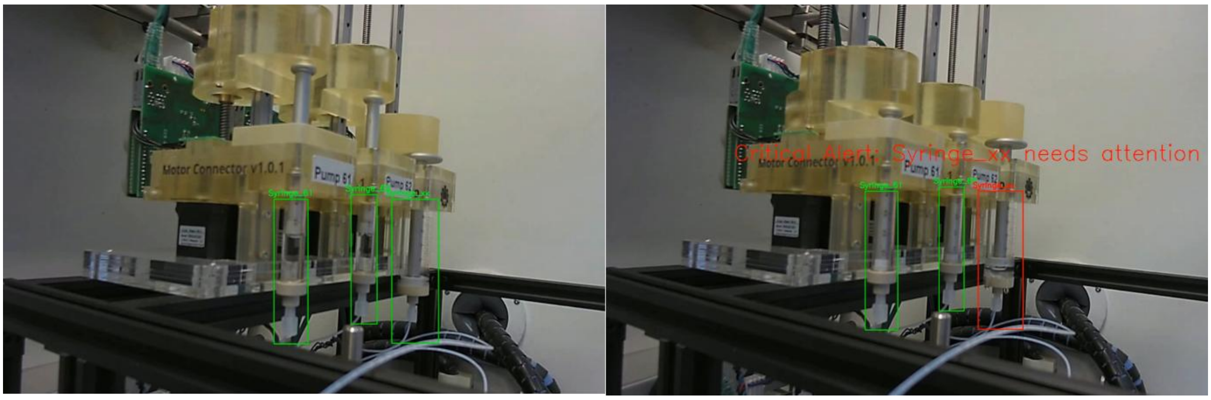

Fig. S25. Anomaly detection via computer vision.

5.3.2 In-line Filtration

This experiment was performed to assess the ability of the liquid sensor to track liquid levels during ChemPU operation. Besides the dedicated filter modules, ChemPU reactor modules can used to filter off solids by equipping them with an in-line filter (Bottom-of-the-Bottle™ Solvent Filter UHMWPE, 10µm, for 1/8" OD, IDEX Part No. A-446). This filtration is often less efficient and requires slow transfer speeds and/or excess volume to be transferred. Appropriate parameters for a given reaction mixture typically must be established empirically. A potential use case for a liquid sensor would be to qualitatively establish whether liquid is being transferred. Quantitatively tracking the volume transferred could then indicate whether the liquid was transferred successful or if the line was clogged to automatically append troubleshooting steps (such as stirring to break a filter cake, solvent washes to dissolve precipitate, or additional transfer steps with adjusted speeds and volumes). The viability of such an approach was tested by executing a simple `Filter` step in two scenarios, typically for drying organic phases after work-up and a challenging suspension of inorganic base in organic solvent, common in cross-coupling reactions:

- 10g of $MgSO_4$ in 100 mL EtOAc
- 10g of K_3PO_4 in 100 mL THF

These suspensions were filtered using the following XDL step:

```
<Filter
  vessel="reactor"
  filtrate_vessel="collection"
  stir="True"
```

```
  stir_speed="0 RPM" />
```

Fig. S26. Filter step used to test the liquid sensor's ability of tracking liquid handling.

A 250 mL 3-neck round bottom flask served as the reactor vessel. The maximum volume of the reactor was set to 100 mL in the hardware graph, by default, this volume will be transferred in the `Filter` step. A 100 mL measuring cylinder was used to manually record the volumes transferred (+/- 1 mL). The process was repeated with three different values for the `stir_speed` parameter: `default`, `0 RPM`, `50 RPM` (Table S4).

Table S4. Results of the in-line filtration test.

System	Stir speed	Volume transferred [mL]
$MgSO_4$ / EtOAc	"default"	38
$MgSO_4$ / EtOAc	0 rpm	83
$MgSO_4$ / EtOAc	50rpm	66
K_3PO_4 / THF	"default"	82.5
K_3PO_4 / THF	0 rpm	88
K_3PO_4 / THF	50rpm	81

The liquid sensor data was captured. A threshold of 450 was used to partition the data into two classes, representing the filled and empty state of the tubing. Simply counting the number of data points where the tubing was filled, gave an excellent correlation with the experimentally measured volumes.

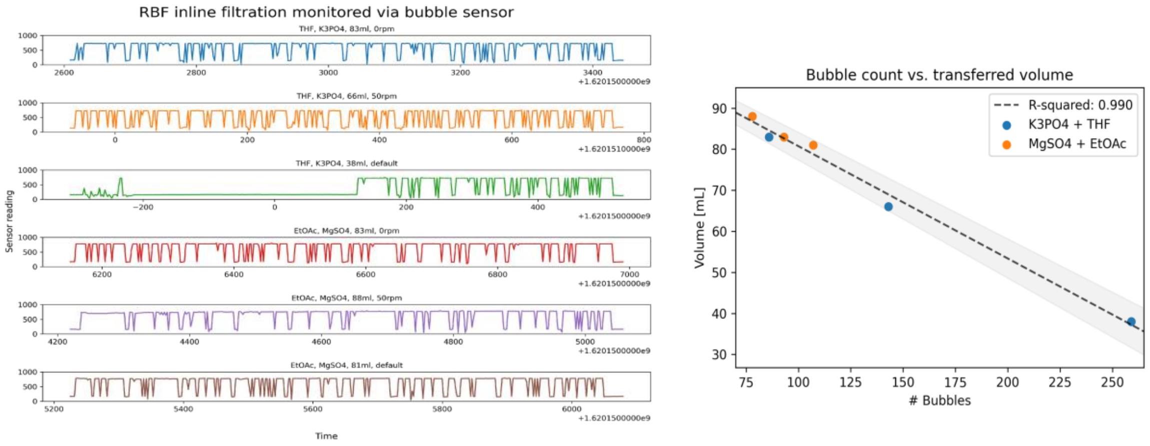

Fig. S27. Left: Raw sensor traces for the in-line filtration steps monitored with the liquid sensor. Right: Correlation of the transferred volume with the number of bubbles counted.

The findings indicate that qualitative and quantitative liquid tracking using the liquid sensor are feasible and could help the execution of challenging filtration steps. To establish robust routines ready for use in production, more data needs to be gathered and extensive testing needs to be performed.

5.3.3 Turbidity monitoring

The ability of the RGBC colour sensor to detect useful properties such as turbidity was investigated using Formazine turbidity standard solutions. For this, the RGB sensor was attached to a round bottom flask and filled with a turbidity standard solution. 30 data points were acquired. This process was repeated three times for each of the 10 NTU, 100 NTU and 1000 NTU standard solutions. It was found that depending on the background, ambient light and deviations in the sensor positioning, the absolute values of the sensor readings varied significantly and overlapped for different turbidity standard solutions. While absolute values appeared unsuitable to assess the turbidity of a solution, the sensor could be used to monitor relative increases in turbidity, which would still provide meaningful process information. To test this potential use case, Formazine, a heterocyclic polymer used for as a turbidity standard, was synthesized in the ChemPU using XDL.

5.3.4 Data Verification

Throughout the project described in this publication, we have collected large quantities of sensor data. Figure S27 demonstrates the data from several sensors for 2 trifluoromethylation reactions (section 5.5.2) executed with identical starting reaction parameters with reproducible results (according to the final ^{19}F NMR). Different areas of interest are highlighted on the graph:

1) Reagent’s addition (highlighted as green) is clearly indicated on the liquid sensor plot with high readings at the time, the reagent was in the corresponding tube. Unfortunately, the default low reading frequency (1 per second) was not sufficient for quantitative analysis.
2) Cooling (highlighted as grey blue) is seen on the internal temperature sensor for both reactions. Different cooling time is caused by the change in the ambient temperature.
3) Catalyst addition (0.1 M TBAF in THF, highlighted as black dotted line) is seen on each plot: temperature raise due to exothermic reaction, fast decay of the colour (i.e., reaction mixture turned dark brown) and several spikes on the liquid sensor referring to the catalyst addition, tube flushing and subsequent backbone cleaning.

4) Heating (highlighted as orange pink) is seen on the internal temperature sensor for both reactions. Here the "room temperature" was fixed at 22 °C in the procedure description.
5) Work-up (i.e., excess TMSCF3 cleavage with 1.0 M TBAF solution in THF, highlighted as black dotted like) is visualized on each plot: temperature raise due to exothermic reaction, small decay on the colour sensor plot and several spikes on the liquid sensor attributed to the reagent addition, tube flushing and subsequent backbone cleaning.
6) Reactor cleaning (highlighted as violet blue) is visualized the most on the colour and liquid sensor: significant increase (i.e., reactor environment turning from dark brown to transparent) and several spikes attributed to various liquid movements respectively.

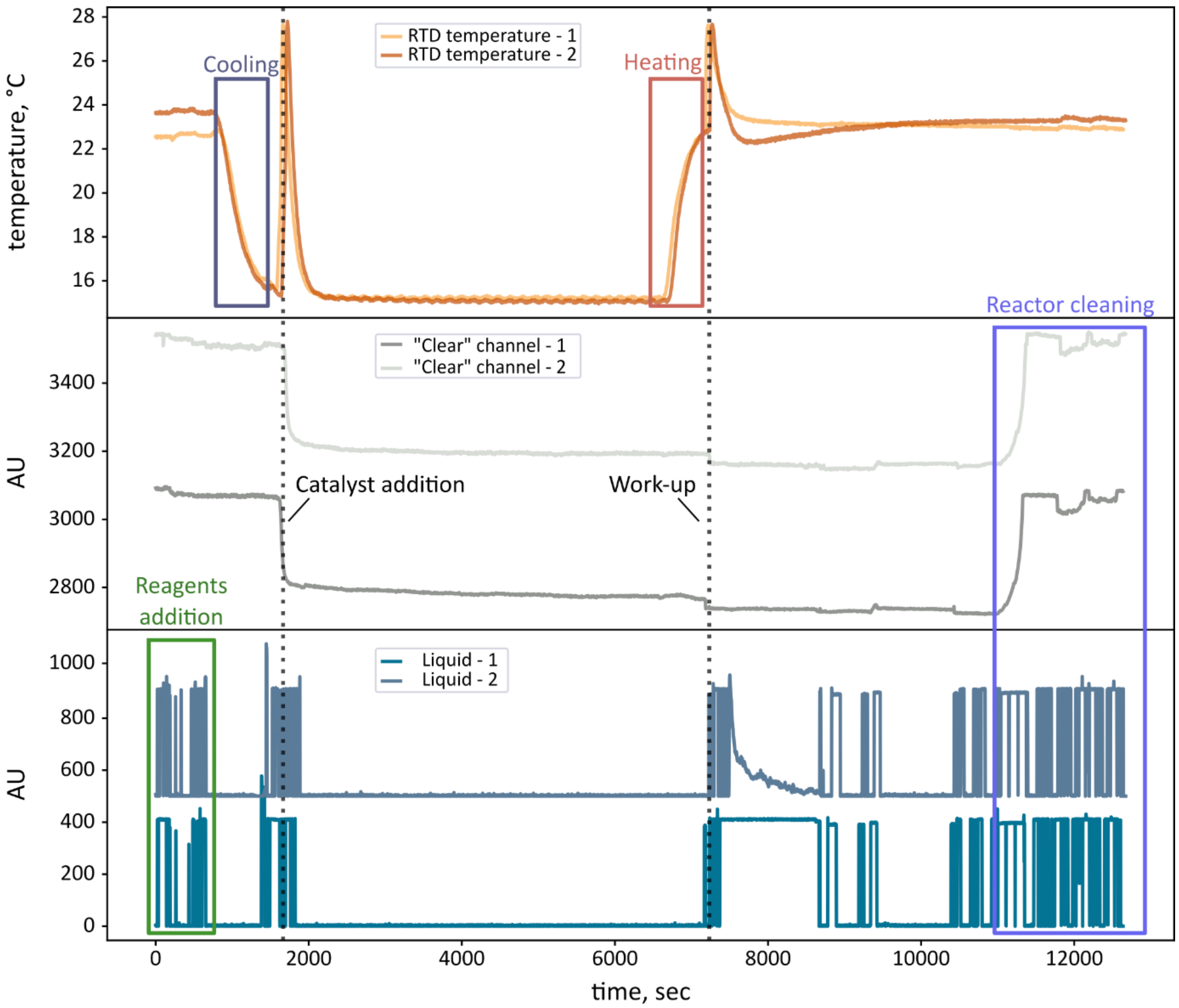

Fig. S28. An example of the data read from 3 sensors: internal temperature probe ("RTD Temperature"), unfiltered data of the RGB colour channel ("Clear channel") and liquid sensor ("Liquid") for 2 trifluoromethylation experiments (see section 5.5.2), run with identical starting parameters. The data for the liquid sensor ("Liquid – 2") was artificially increased by 500 for clear presentation. The difference in the absolute values for the colour sensor readings are caused by the difference of the ambient light setup.

At the same time the visualization of the sensor data allows to identify an error in the procedure. Figure below (**Fig. S29**) shows an example plots of the sensor data obtained for the trifluoromethylation reaction (see section Trifluoromethylation5.5.2) executed with similar procedure parameters (only 1.5 °C reaction temperature difference), however demonstrating completely different results during the reaction mixture analysis using ^{19}F NMR. Similar to figure S27, various steps of the procedure can be seen on the plots, however, the profile for the heating and work-up is completely different for the 2 reactions. This may be explained by a hardware failure during reagent addition of cyclohexenone, however the data from the liquid sensor was not sufficient to prove it.

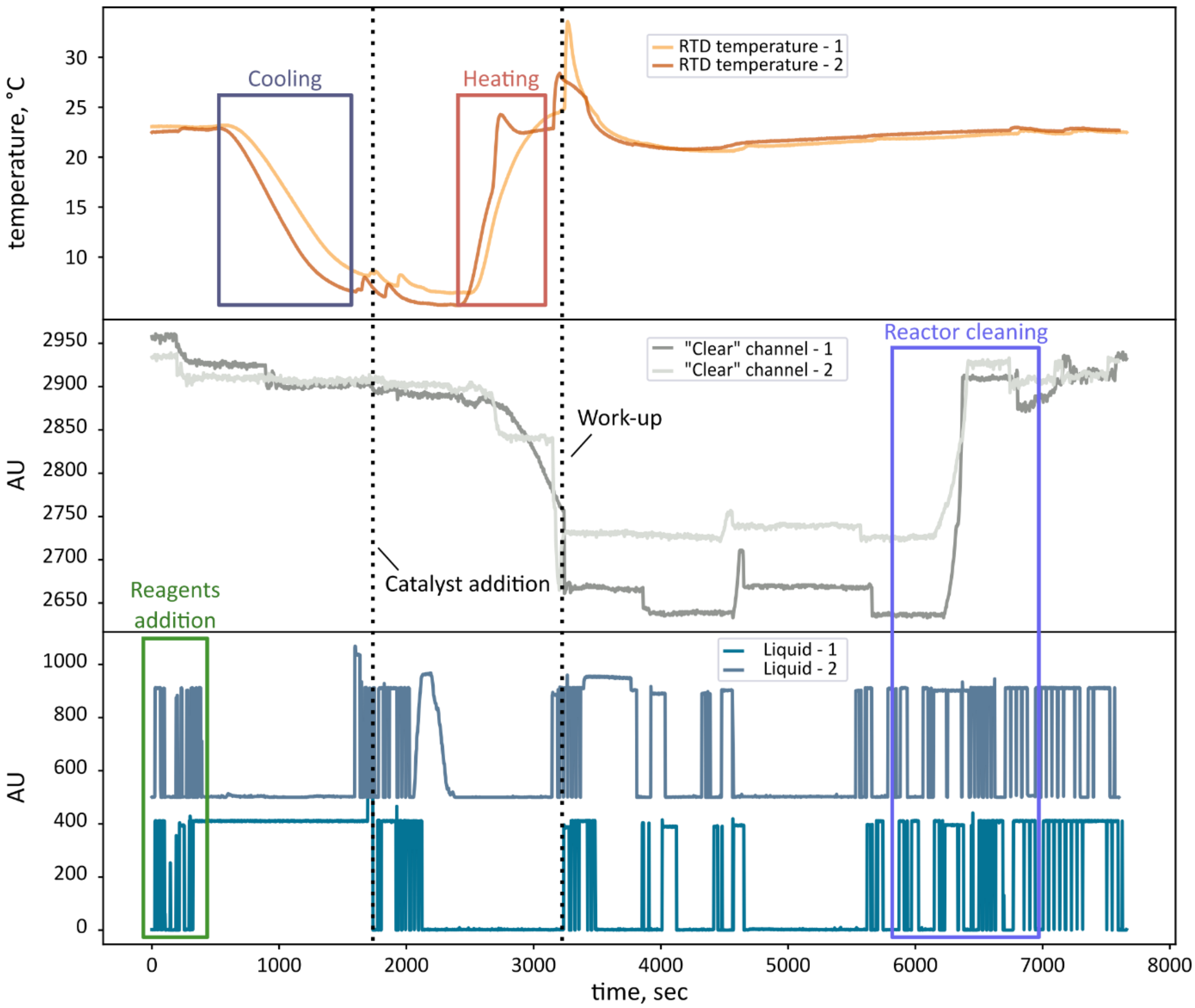

Fig. S29. An example of the data read from 3 sensors: internal temperature probe ("RTD Temperature"), unfiltered data of the RGB colour channel ("Clear channel") and liquid sensor ("Liquid") for 2 trifluoromethylation experiments (see section 5.5.2), run with identical starting parameters. The data for the liquid sensor ("Liquid – 2") was artificially increased by 500 for clear presentation.

The current implementation of the dynamic execution does not provide any routine to validate the executed procedure against already performed experiments. We propose a machine learning algorithm to identify regions of interest in the obtained data that can serve as a unique reaction fingerprint, validate the executed procedure in real-time and detect potential outliers and deviations in the data stream. Such algorithm should also be able to tolerate insignificant changes in the ambient conditions as well as filter the incoming raw data.

5.4 Dynamic procedure execution

Beyond real-time monitoring, we found that sensors could be used for dynamic execution of synthetic procedures. This ability was exemplified in two use cases: i) maintaining the internal temperature below a given threshold during an exothermic thioether oxidation ii) waiting for a

colour change, thus dynamically adjusting the reaction time, in an iodine-consuming nitrile formation reaction.

5.4.1 Thioether oxidation

The oxidation of dibenzyl sulphide to dibenzyl sulfone was selected to highlight the benefits of the incorporating an RTD probe into the ChemPU stack in the context to dynamic addition steps (Scheme S3).

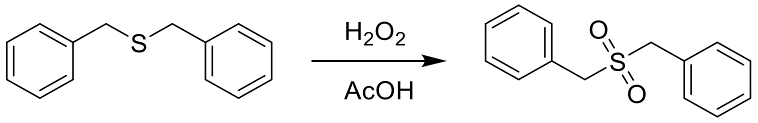

Scheme S3. Temperature-controlled thioether oxidation reaction.

The literature procedure was translated to XDL, however the key step of this process is the temperature-controlled addition of hydrogen peroxide. The literature procedure states that this step should be performed with the internal not exceeding 75 °C. To emulate this behaviour in the ChemPU the `AddDynamic` step with the following parameters was used:

```
<AddDynamic
  vessel="reactor"
  reagent="30 % hydrogen peroxide solution"
  volume="35.0 mL"
  sensor="rtd"
  reading="temperature"
  reading_threshold="75.0"
  safety_margin="10.0"
  aliquot_volume="2.0 mL"
  wait_after_addition="10.0"
  settle_delay="10.0"
  trend="ascending"
/>
```

Preliminary experiments on a 2.5 g scale showed that the process was strongly exothermic but did not generate enough heat to cross the specified threshold in our setup, however a 10-fold scale up would require dynamic temperature control to prevent the thermal runaway.

5.4.1.1 Description of the automated synthesis

The reactor was manually charged with 25 g of dibenzyl sulphide. The subsequent actions were performed in a fully automated fashion: The starting material was dissolved in glacial acetic

acid (100 mL) at 30 °C. After cooling to room temperature, a total of 35 mL of 30% aqueous hydrogen peroxide solution was added in 2 mL portions while maintaining the internal reaction temperature below 75 °C. After the addition, the reaction mixture was stirred at 85°C for 3 hours and subsequently cooled back to room temperature, allowing the product to precipitate.

5.4.1.2 Results

The exothermic addition was controlled using the dynamic XDL step. As soon as the temperature reached the threshold, the addition paused until a safe temperature for continued addition was reached. The passive monitoring further highlighted an uncontrolled exotherm upon heating the reaction mixture to 85 °C as required by the literature procedure. This example shows the crucial importance of sensor feedback for safe and scalable processes. The crystallized product in the reactor flask was manually filtered, washed with cold water and dried under vacuum (27.26 g, 94.9%).

^{1}H NMR (400 MHz, $CDCl_3$): δ 7.81 – 6.83 (m, 10H), 4.05 (s, 4H).

^{13}C NMR (101 MHz, CDCl3): 131.0, 129.1, 129.1, 127.6, 58.1.

5.4.2 Nitrile formation

An iodine-consuming nitrile formation reaction was chosen to illustrate the utility of the RGB sensor in the context of dynamic procedure execution.

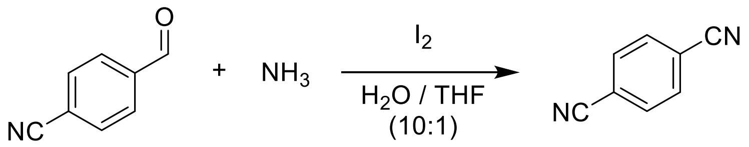

Scheme S4. Nitrile formation reaction monitored via RGB sensor.

The general literature procedure was translated to XDL. Instead of specifying a reaction time, the authors noted that the reaction was stirred until the colour disappeared. This was emulated using the following step sequence featuring the dynamic `DoUntil` step:

```
<TakeBackgroundReading
  sensor="rgb"
  reading="clear" />
<Add
  vessel="reactor"
  reagent="1M iodine thf solution"
  volume="1.1 mL"
  stir="True"
  priming_volume= "1.0 mL"
  aspiration_speed="1"
/>
<DoUntil sensor="rgb" threshold="0" margin="100" reading="clear">
  <Stir
    vessel="reactor"
    stir_speed="300"
    time="60 mins" />
</DoUntil>
```

5.4.2.1 Description of the automated synthesis

The reactor was manually charged with 4-Formylbenzonitrile (131 mg, 1.0 mmol, 1.0 eq.,). The subsequent actions were performed in a fully automated fashion: Aqueous ammonia solution (~25%, 10 mL) was added. A background measurement for the RGB sensor was taken. 1 M Iodine in THF solution (1.1 mL, 1.1 mmol, 1.1 eq.) was added. The reaction was stirred until the RGB sensor reading reached the background level within the margin specified in the procedure, indicating the full consumption of iodine reagent and end point of the reaction. The reaction mixture was quenched using 5% aqueous sodium thiosulfate solution and subsequently extracted using 2 × 15 mL of diethyl ether using the reactor and separator modules. The combined organic phases were collected in an empty holding flask.

Caution: The reaction of ammonia and iodine forms nitrogen triiodide, an extremely sensitive contact explosive. As such, the process described above should be performed with a minimal excess of iodine and quenched with excess thiosulfate.

5.4.2.2 Results

The combined organic phases obtained in this process concentrated manually, yielding pure product (90.2 mg, 70.4%).

^{1}H NMR (400 MHz, d_6-DMSO): δ 8.04 (s, 4H).

^{13}C NMR (101 MHz, d_6-DMSO): δ 133.44, 117.80, 115.92.

5.5 Closed-loop reaction optimization

A series of established, modified and new synthetic procedures were subjected to autonomous reaction optimization using integrated process analytical technology, yielding improved XDL procedures.

5.5.1 Ugi four-component reaction

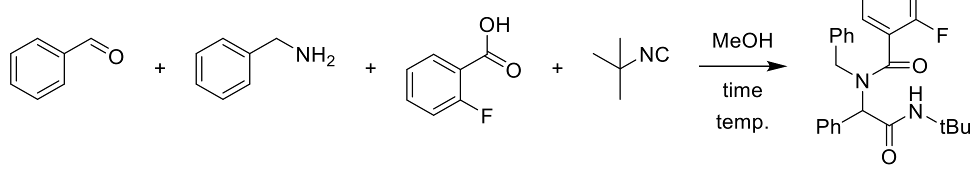

Scheme S5. Four-component Ugi reaction.

The four-component Ugi reaction was selected as a complex synthesis with large number of input parameters. The original procedure[23] was translated into XDL file and amended for running iterative optimization (see Appendix B, Fig.). All reagents were used neat unless otherwise stated. The liquid handling system of the ChemPU was equipped with 1/16” PTFE tubing and corresponding selection valves (IDEX V-240) for precise reagent delivery. 2-Fluorobenzoic acid was selected to allow reaction analysis using ^{19}F NMR.

5.5.1.1 Optimization parameters

For the iterative closed-loop optimization the following reaction parameters were set:

- Benzaldehyde volume – fixed at 0.2 mL (to ensure maximum product amount of 2.0 mmol).
- Methanol volume – variable within 0.0 and 5.0 mL range.
- Benzylamine volume – variable within 0.1 and 1 mL range.
- Imine formation time – variable within 0.0 and 1800.0 seconds range.
- 2-Fluorobenzoic acid volume (2.0 M in methanol) – variable within 0.5 and 3.0 mL range.
- *tert*-Butylisocyanide volume – variable within 0.1 and 1.0 mL range.
- Reaction temperature – variable within 25.0 and 60.0 °C range.
- Reaction time – variable within 7200.0 and 64800.0 seconds.

- 1,4-Difluorobenzene (0.2 M solution in DCM) – fixed at 5.0 mL (as 1:2 ratio with benzaldehyde).

The procedure was executed using interactive python environment, each experiment consisted of 5 consecutive reactions with manual reagent refilling in between (or earlier if required by the experiment). The following strategies for optimization were used (full configuration files are available in the Appendix C): 5 random search, 14 SMBO explorative experiments, 5 SMBO balanced search and 6 SMBO exploitation experiments. The reaction was analyzed using ^{19}F NMR and the optimization was set to maximize the peak of the Ugi product (at -114.3 ppm), with 1,4-difluorobenzene as internal standard (-119.2 ppm), as stated in section 1.6.3.

5.5.1.2 Description of the automated synthesis

Three-neck 25-mL round bottom flask (reactor) equipped with reflux condenser, glass stopper, tubing connector to a liquid handling system, DrySyn© aluminium block and a magnetic stirrer bar. In the beginning of the procedure the liquid handling system was washed with methanol. Benzaldehyde (0.20 mL, 1.96 mmol) was added automatically, following by benzylamine (0.22 mL, 2.09 mmol) and methanol (5.0 mL). The reaction mixture was stirred for 10 minutes and 2-fluorobenzoic acid (2.0 M in methanol solution, 1.00 mL, 2.00 mmol) was added, following by *tert*-butyl isocyanide (0.23 mL, 2.03 mmol). The resulting mixture was stirred for 18 hours at 25 °C. Thereafter the 1,4-difluorobenzene (0.2 M in DCM, 5.00 mL, 2.00 mmol) was added and the sample (2.5 mL) of the resulting mixture was transferred to the NMR for analysis. Upon analysis completion, the sample was transferred back to the flask and all its contents was moved to an empty flask for storage. The reactor was cleaned twice with DCM (15 mL) and used for the next iteration.

5.5.1.3 Results

The optimization was terminated after 30 iterations, achieving 38% yield increase (full table of results is given in the Appendix D).

^{1}H NMR (400 MHz, CDCl3): δ 7.46 – 7.40 (m, 2H), 7.33 – 7.15 (m, 7H), 7.10 – 7.00 (m, 4H), 6.87 – 6.82 (m, 1H), 5.92 (s, 1H), 5.72 (s, 1H), 4.63 (d, J = 16.4 Hz, 1H), 4.41 (d, J = 16.4 Hz, 1H), 1.35 (s, 9H).

^{13}C NMR (101 MHz, CDCl3): δ 168.5, 168.2, 158.3 (d, J_{CF} = 247.3 Hz), 136.9, 135.1, 131.2 (d, J_{CF} = 7.9 Hz), 129.6, 128.8, 128.7, 128.6, 128.5, 128.3, 127.8, 127.1, 124.8 (d, J_{CF} = 17.4 Hz), 124.5 (d, J_{CF} = 3.0 Hz), 115.9 (d, J_{CF} = 21.1 Hz), 64.4, 52.2, 51.7, 28.7.

^{19}F NMR (376 MHz, CDCl3) δ -115.1.

The spectroscopic data is in agreement with previously published[24].

5.5.2 Van Leusen oxazole synthesis

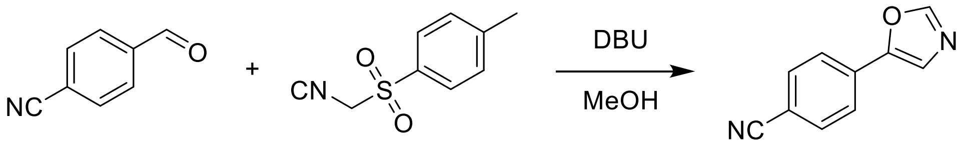

Scheme S6. Van Leusen oxazole synthesis.

The Van Leusen oxazole reaction was selected as an example for the synthesis of heterocyclic building blocks, common in the medicinal chemistry literature. The procedure was loosely based on a patent[25], translated into XDL and adapted for running iteratively (see Appendix B). Notably, it features a small volume addition (as low as 150 μL) that commonly requires manual calibration or optimization. To make the problem more challenging, the purity of the product mixture was optimized as a secondary objective to implicitly minimize excess reagent used. Furthermore, this example was selected as a test case for the new parallel scheduling capabilities described in Section 1.6.4. The liquid handling system of the ChemPU was equipped with 1/16” PTFE tubing and corresponding selection valves (IDEX V-240) for precise reagent delivery. Naphthalene was selected as an internal standard to allow reaction analysis using HPLC-DAD at 254 nm.

5.5.2.1 Optimization parameters

For the iterative closed-loop optimization the following reaction parameters were set:

- TosMIC volume – variable within 4.10 and 6.15 mL range (1.0 - 1.5 eq.)
- DBU volume – variable within 0.15 and 0.31 mL range (1.0 - 2.0 eq.)
- Reaction temperature – variable within 25.0 and 75.0 °C range.
- Reaction time – variable within 1800.0 and 10800.0 seconds.

As an optimization target was set the weighted sum of the peak purity and product peak area (14.5 min retention time) relative to the peak area of the internal standard (naphthalene, 20 min

retention time), see **Eq. S1** where *AUC* refers to the area under the curve for a given peak, w is a weighing factor set to 0.7 and c is constant to scale the two objective values to the same order of magnitude set to 0.1. The optimization algorithm was SNOBFIT as implemented in the Summit framework.

$$Target = w * c * \frac{AUC_{product}}{AUC_{reference}} + (1 - w)\frac{AUC_{product}}{\sum_{i=1}^{N} AUC_{Peak\ i}}$$

Eq. S1: Formula to calculate the objective value for the Van Leusen Optimization.

5.5.2.2 Description of the automated synthesis

The process was executed automatically using 2 parallel reactors. 0.25 M TosMIC in MeOH solution (variable), 0.25 M 4-formylbenzonitrile in MeOH (4.1 mL, containing 0.05 M naphthalene as an internal standard), neat DBU (variable) and methanol (5 mL) were added to the reactor. The reaction mixture was stirred for a variable amount of time at a variable temperature. After cooling to room temperature, a sample (0.2 mL) is withdrawn for the reactor, transferred to an empty flask, diluted to 20.0 mL, and subsequently loaded onto a 5 μL sample loop and injected into the HPLC. The remaining volume of the reaction mixture was discarded, and the platform reset by cleaning all modules with methanol and/or acetonitrile.

5.5.2.3 Results

The optimization campaign was concluded after 26 iterations, achieving a 10% increase in the weighted objective when comparing the best to the first iteration or 55% when comparing the best to the worst iteration. (full table of results is given in the Appendix D).

^{1}H NMR (400 MHz, $CDCl_3$): δ 7.92 (s, 1H), 7.79 – 7.56 (m, 4H), 7.44 (s, 1H).

^{13}C NMR (101 MHz, $CDCl_3$): δ 151.66, 149.87, 132.96, 131.80, 124.81, 124.33, 118.55, 112.10.

5.5.1 Burgress manganese-catalysed epoxidation

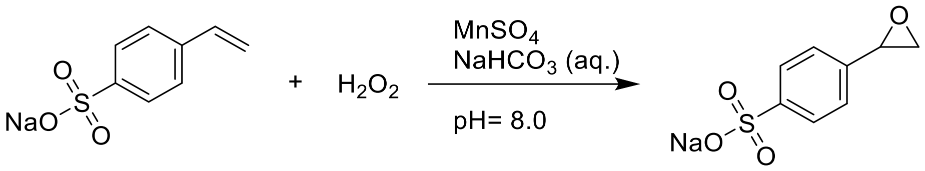

Scheme S6. Manganese-catalysed styrene sulfonate epoxidation.

The Mn-catalysed epoxidation of olefins is a reaction of industrial interest[26] and has been extensively described by Burgess et al.[27] for a variety of substrates. This example was selected because the active catalytic species is formed in situ and under the wrong conditions, manganese gets oxidized and decomposes hydrogen peroxide, leading to poor conversions.[28] In our optimization we try to find optimal conversion conditions by changing 4 major parameters of the reaction. The liquid handling system of the ChemPU was equipped with 1/16" PTFE tubing and corresponding selection valves (IDEX V-240) for precise reagent delivery. Acetonitrile was used as an internal standard and the reaction was analyzed Raman spectroscopy.

5.5.1.1 Optimization parameters

For the iterative closed-loop optimization the following reaction parameters were set:

- Catalyst volume – variable within 0.5 and 5.0 mL range (0.2 - 1.0 mol%).
- $NaHCO_3$ volume – variable within 0.5 and 5.0 mL range.
- H_2O_2 volume – variable within 1.0 and 5.0 mL range (4 - 20 eq.).
- H_2O_2 addition speed – variable within 0.04 and 10.00 mL/min range.
- Reaction time – variable within 3600.0 and 36000.0 seconds.

The optimization target was to minimize the area of the peak corresponding to the double bond of the starting material at 1633 cm^{-1} relative to the area of the peak at 2261 cm^{-1}, corresponding to the triple bond of acetonitrile which was used as an internal standard. Phoenics[29] optimization algorithm was chosen; the optimization was run for 40 iterations.

5.5.1.2 Description of the automated synthesis

5 mM MnSO4 in water solution (variable), 0.5 M styrene sulfonate in water solution (5 mL), and 0.5 M $NaHCO_3$ in water solution (variable) were automatically transferred to a reactor

vessel, placed in front of a Raman probe. 30% Hydrogen peroxide in water (variable) was added at variable speed. The reaction mixture was stirred for a variable time. The process was continuously monitored via Raman and single end-point analysis was taken for calculating the optimization target value. The reaction mixture was discarded, and the platform was reset by cleaning the reactor vessel with 1 × 25 mL cleaning solution and 2 × 25 mL water. The cleaning solution was made by mixing 100 mL 30% H_2O_2, 100 mL 60% nitric acid, 800 mL H_2O.

5.5.1.3 Reproducibility

The two best results (iteration 10 and 25) were repeated to check their reproducibility leading to the discovery of an outlier (iteration 10) and the confirmation that the reaction goes to completion in the case of the iteration 25.

Reproducibility experiments are listed in Table S5:

Table S5. Control experiments for the styrene epoxidation reaction

$NaHCO_3$ (mL)	$MnSO_4$ (mL)	H_2O_2 (mL)	H_2O_2 (addition rate) (mL/min)	Duration (s)	Results (a.u.)	Std dev [Std err]
3.4905	4.9984	4.9955	0.2401	1827.9069	-0.3608	0.03 [0.012]
3.4905	4.9984	4.9955	0.2401	1827.9069	-0.3984	
3.4905	4.9984	4.9955	0.2401	1827.9069	-0.4199	
3.4905	4.9984	4.9955	0.2401	1827.9069	-0.3428	
3.4905	4.9984	4.9955	0.2401	1827.9069	-0.3731	
3.4905	4.9984	4.9955	0.2401	1827.9069	-0.347	
3.6246	4.8424	4.8127	0.0539	1829.4752	-0.1258	0.015 [0.006]
3.6246	4.8424	4.8127	0.0539	1829.4752	-0.1123	
3.6246	4.8424	4.8127	0.0539	1829.4752	-0.1162	
3.6246	4.8424	4.8127	0.0539	1829.4752	-0.1396	
3.6246	4.8424	4.8127	0.0539	1829.4752	-0.1463	
3.6246	4.8424	4.8127	0.0539	1829.4752	-0.1439	

5.5.1.4 Results

^{1}H NMR (400 MHz, D_2O): δ 7.82 (dd, *J* = 8.3, 1.4 Hz, 2H), 7.46 (d, *J* = 8.4 Hz, 2H), 4.10 (dd, *J* = 4.4, 2.9 Hz, 1H), 3.30 (t, *J* = 4.5 Hz, 1H), 3.05 (dd, *J* = 4.7, 2.9 Hz, 1H).

^{13}C NMR (101 MHz, D_2O): δ 142.36, 140.35, 126.36, 125.73, 52.56, 51.49.

HRMS-ESI: 199.0076 (Expected: 199.0071)

5.5.2 Trifluoromethylation

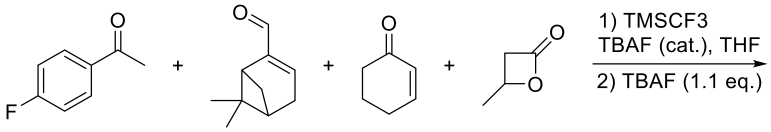

Scheme S7. Carbonyl compounds and conditions used for explorative optimization of the trifluoromethylation reaction.

The trifluoromethylation reaction using the Ruppert-Prakash reagent was setup to demonstrate the capability of the Optimizer framework to discover new product in the product space and optimize each individual product in a closed-loop fashion using feedback from benchtop NMR. The original procedure[30] was amended to include an alternative work-up process[31] to reduce overall experiment time. Reagents were selected to provide several sides of attack as well as a scope for novel materials. Experiments were run as follows:

1. Initial exploration of the product space using random search within given parameter limits.

2. Exploration of the product space using SMBO algorithm (with parameters setup to favour exploration over exploitation) and a *novelty* parameter as the target.

3. Exploitation of the product space using SMBO algorithm and a *novelty* parameter as the target.

4. Identification of the obtained materials as identified regions of interest on the resulting ^{19}F NMR spectra. For each experiment result was calculated as the integration area of the target region divided by the sum of all other regions identified.

5. Individual optimization using SMBO algorithm (with parameter setup to favour exploitation over exploration) and product peak integration area as a target. The data from all previous experiments (i.e., set of parameters and corresponding results) were used in each subsequent optimization.

5.5.2.1 Optimization parameters

The parameters for the iterative closed-loop optimization were selected to allow for product formation from individual starting materials as well as theoretical mixtures. The following parameters were set:

- Cyclohexenone volume – variable within 0.0 and 0.3 mL range.
- 4-Fluoroacetophenone volume – variable within 0.0 and 0.37 mL range.
- Beta-butyrolactone volume – variable within 0.0 and 0.23 mL range.
- Myrtenal volume – variable within 0.0 and 0.45 mL range.
- THF volume – variable within 0.0 and 10.0 mL range.
- Pre-heat reaction temperature – variable within 0.0 and 50.0 °C range.
- Trifluoromethyltrimethylsilane volume – variable within 0.36 and 1.42 mL range.
- TBAF (0.1 M in THF solution) volume – variable within 0.2 and 0.8 mL range.
- Reaction time – variable within 300.0 and 18000.0 seconds.
- TBAF (1 M in THF solution) volume for work-up – variable within 2.0 and 10.0 mL range.
- Work-up time – variable within 300.0 and 3600.0 seconds.
- Fluorobenzene (1 M solution in DCM) – fixed at 2.0 mL.

5.5.2.2 Description of the automated synthesis

Three-neck 25-mL round bottom flask (reactor) equipped with reflux condenser, temperature probe, tubing connector to a liquid handling system, DrySyn© aluminium block (connected to a chiller) and a magnetic stirrer bar. In the beginning of the procedure the liquid handling system was washed with THF. Cyclohexenone (0.20 mL, 1.0 mmol) was added automatically, following by 4-fluoroacetophenone (0.23 mL, 1.0 mmol), butyrolactone (0.16 mL, 1.0 mmol), myrtenal (0.30 mL, 1.0 mmol) and THF (5 mL). The reaction mixture was adjusted to 22 °C and triflouoromethyltrimethylsilane (0.9 mL, 6.1 mmol) was added, following by 0.1 M solution of TBAF in THF (0.4 mL, 0.04 mmol). The resulting mixture was stirred for 5 minutes at maintained temperature. Thereafter the reaction mixture was adjusted to 22 °C, 1.0 M TBAF solution in THF (3.0 mL, 3.0 mmol) was added to cleave TMS group and the reaction mixture was stirred for another 5 minutes. The solution of fluorobenzene (1.0 M in DCM, 2.0 mL, 2.0 mmol) was added and the sample (2.5 mL) of the resulting mixture was transferred to the NMR for analysis. Upon analysis completion, the sample was transferred back to the flask and all its

contents was moved to an empty flask for storage. The reactor was cleaned twice with THF (20 mL) and used for the next iteration.

5.5.2.3 Results

All products were isolated from the collection vessel at the end of the corresponding product optimization campaign. The material was concentrated *in vacuo* and purified by preparative HPLC to give the following products. Yields indicated are calculated from the crude reaction mixture analysis with respect to the internal standard (fluorobenzene).

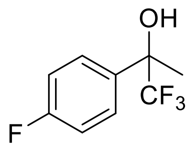

(97%)

^{1}H NMR (400 MHz, $CDCl_3$): *δ* 7.61 – 7.51 (m, 2H), 7.13 – 7.02 (m, 2H), 1.78 (q, *J* = 1.1 Hz, 3H).

^{13}C NMR (101 MHz, $CDCl_3$): *δ* 163.0 (d, J_{CF} = 247.7 Hz), 134.3 (d, J_{CF} = 3.1 Hz), 128.5 – 128.1 (m), 125.6 (q, J_{CF} = 285.2 Hz), 115.3 (d, J_{CF} = 21.5 Hz), 74.7 (q, J_{CF} = 29.5 Hz), 24.1.

^{19}F NMR (376 MHz, $CDCl_3$): *δ* -81.3, -113.7 – -113.8 (m).

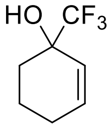

(67 %)

^{1}H NMR (400 MHz, CDCl3): *δ* 6.2 (ddd, J = 10.1, 4.9, 2.8 Hz, 1H), 5.7 (ddt, J = 10.1, 2.8, 1.6 Hz, 1H), 2.2 – 2.0 (m, 2H), 1.9 – 1.7 (m, 4H).

^{13}C NMR (101 MHz, CDCl3): *δ* 136.5, 126.2 (q, J_{CF} = 284.2 Hz), 123.0 (q, J_{CF} = 1.7 Hz), 70.6 (q, J_{CF} = 28.8 Hz), 29.1 – 29.0 (m), 24.9, 17.3.

^{19}F NMR (376 MHz, $CDCl_3$): *δ* -82.8.

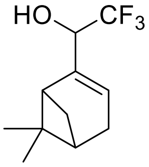

(74%)

^{1}**H NMR** (400 MHz, CDCl3), mixture of isomers: δ 5.81 – 5.73 (m, 1H), 4.41 – 4.30 (m, 1H), 2.50 – 2.30 (m, 4H), 2.16 – 2.09 (m, 1H), 2.05 – 1.98 (m, 1H), 1.30 (s, 3H), 1.18 (dd, J = 8.8, 6.0 Hz, 1H), 0.87 – 0.81 (m, 3H).

^{13}**C NMR** (101 MHz, $CDCl_3$), mixture of isomers: δ 141.6, 141.4, 125.9, 125.1, 124.5 (q, J_{CF} = 283.1 Hz), 124.4 (q, J_{CF} = 283.1 Hz), 73.7 (q, J_{CF} = 31.4 Hz), 73.1 (q, J_{CF} = 31.4 Hz), 42.4, 41.8, 40.8, 40.7, 38.2, 37.9, 31.9, 31.8, 31.6, 31.6, 26.3, 26.1, 21.1, 20.9.

^{19}**F NMR** (376 MHz, $CDCl_3$), mixture of isomers: δ -77.0, -77.0.

5.5.2.4 Examples of the NMR spectra

In addition to the isolated products, the optimization was carried out to maximize the peaks of the side materials. Figures below demonstrate the examples of NMR spectra with highlighted peaks selected for the optimization.

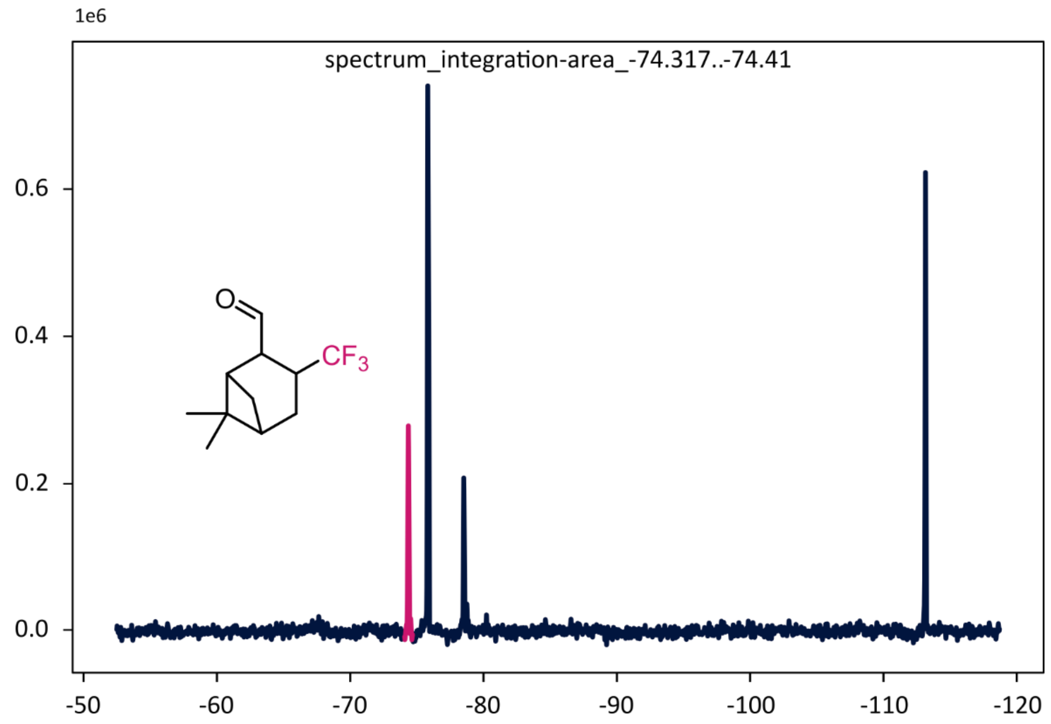

Fig. S30. An ^{19}F NMR spectrum obtained after performing optimization for the identified spectrum region. The product shown has not been isolated as pure material and its structure has not been fully confirmed.

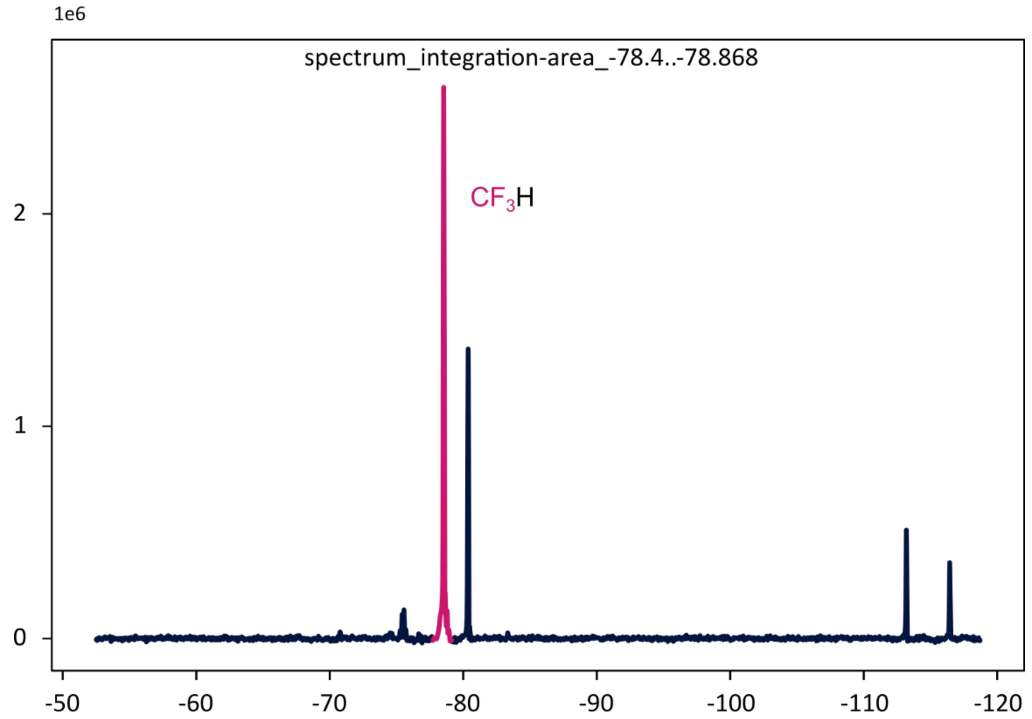

Fig. S31. An ^{19}F NMR spectrum obtained after performing optimization for the identified spectrum region. The product shown has not been isolated as pure material and its structure has not been fully confirmed.

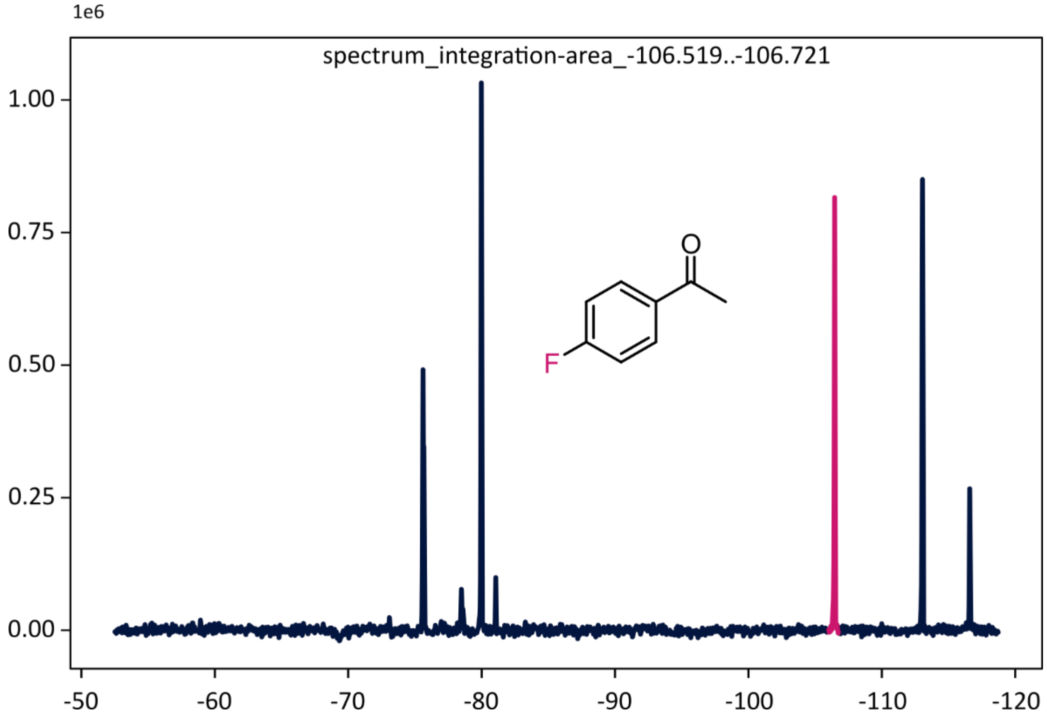

Fig. S32. An ^{19}F NMR spectrum obtained after performing optimization for the identified spectrum region.

5.5.3 Combinatorial reaction discovery

Optimization of de novo reactions was carried out in two chemical spaces, pictured below, both of which were populated with small organic molecules expected to have a wide range of possible reactivity modes and outcomes. Chemical space 1 (Fig. S33) consists of 20 compounds, corresponding to 190 2-component and 1140 3-component combinations (for a total of 1330 unique combinations). Similarly, chemical space 2 (Fig. S34) consists of 24 compounds, corresponding to 276 2-component and 2024 3-component combinations (for a total of 2300 unique combinations). Reactions in both chemical spaces were randomly selected to be performed in our robotic platform, following which the product mixture was analyzed by HPLC/MS and non-trivial cases of reactivity selected for further optimization.

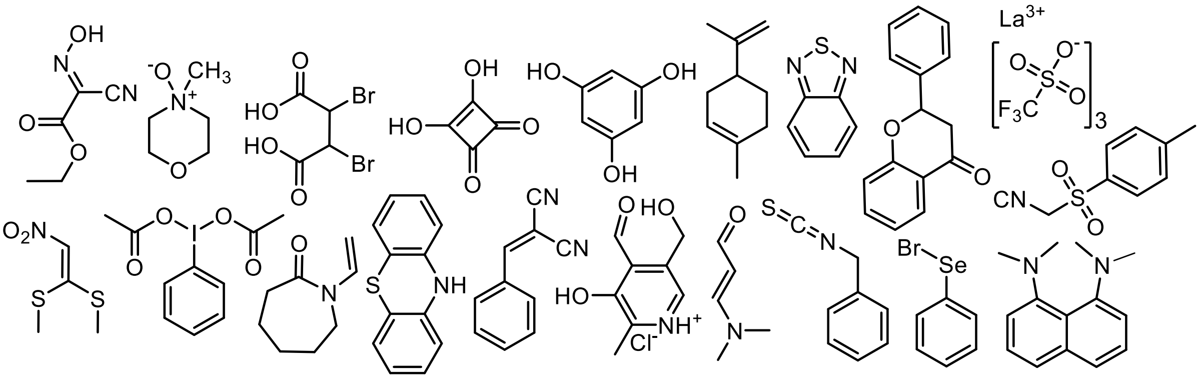

Fig. S33. Chemical space 1.

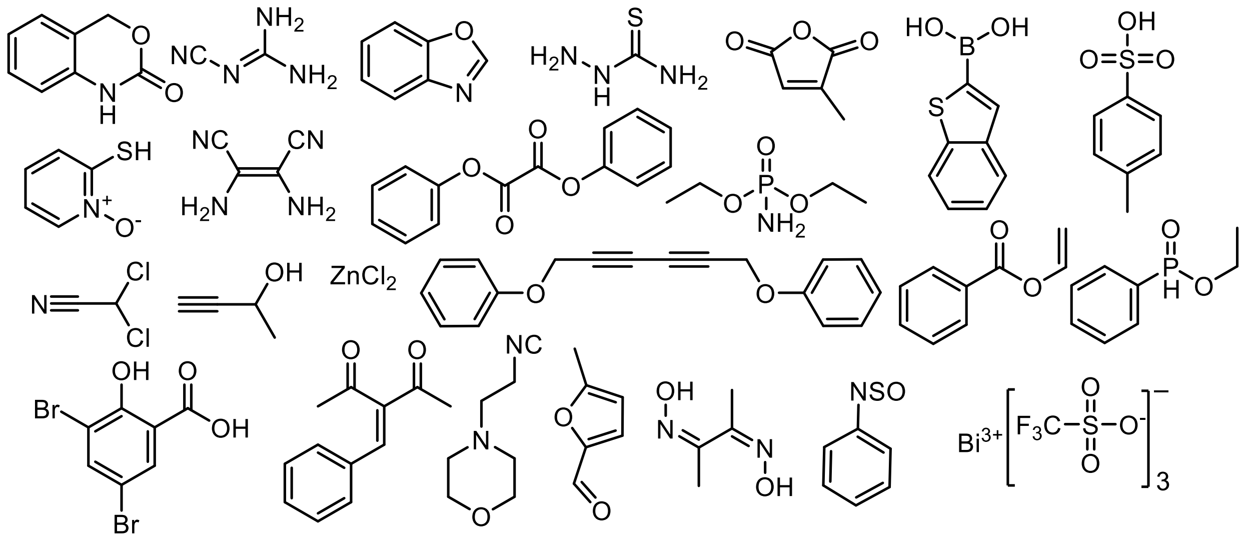

Fig. S34. Chemical space 2.

5.5.4 TosMIC and benzylidenemalononitrile reaction

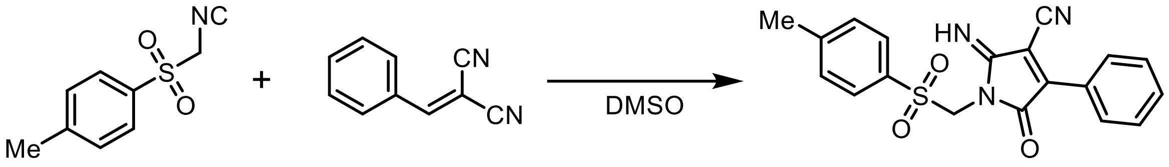

Scheme S8. Reaction of tosMIC with benzylidenemalononitrile.

The reaction between tosMIC and benzylidenemalononitrile was selected following the methodology described in section 5.5.3. The reaction was subjected to optimization, using HPLC to provide necessary feedback to the decision-making algorithms. All reagents were as 1.0 M solution in DMSO, prepared freshly before a set of 5 consecutive iterations. Naphthalene was used as internal standard for quantitative HPLC analysis.

Experiments were run as follows:

1. Initial exploration of the product space using random search within given parameter limits.

2. Exploration of the product space using SMBO algorithm (with parameters setup to favour exploration over exploitation).

3. Identification of the obtained materials as identified regions of interest on the resulting HPLC chromatograms. For each experiment result was calculated as the integration area of the target region divided by the sum of all other regions identified.

5. Individual optimization using SMBO algorithm (with parameter setup to favour exploitation over exploration) and product peak integration area as a target. The data from all previous experiments (i.e., set of parameters and corresponding results) were used in each subsequent optimization.

5.5.4.1 Optimization parameters

- Toluenesulphonylmethyl isocyanide – variable within 0.2 and 2.0 ml range.
- DMSO volume – variable within 0.0 and 5.0 mL range.
- Pre-heat reaction temperature – variable within 30.0 and 160.0 °C range.
- Reaction time – variable within 3600.0 and 28800.0 seconds.

5.5.4.2 Description of the automated synthesis

1.0 M TosMIC in DMSO solution (variable), 1.0 M benzylidenemalononitrile in DMSO (1 mL) and DMSO (variable) were added to the reactor. The reaction mixture was stirred for a variable amount of time at a variable temperature. After cooling to room temperature, reference was added to the mixture (1 mL 1.0 M naphthalene) and afterwards a sample (0.5 mL) is withdrawn for the reactor, transferred to an empty flask, diluted to 10.0 mL, and subsequently loaded onto a 5 µL sample loop and injected into the HPLC. The remaining volume of the reaction mixture was discarded, and the platform reset by cleaning all modules with DMSO.

5.5.4.3 Results

The optimization campaign was concluded after 30 iterations, achieving a 22% increase yield (full table of results is given in Appendix D). Yield was calculated from the crude reaction mixture analysis with respect to the internal standard (naphthalene).

^{1}H NMR (600 MHz, $CDCl_3$): δ 8.12 (d, J = 7.2 Hz, 2H), 7.80 (d, J = 8.3 Hz, 2H), 7.62 (t, J = 7.5 Hz, 1H), 7.55 (t, J = 7.7 Hz, 2H), 7.37 (d, J = 7.9 Hz, 2H), 5.15 (s, 2H), 2.46 (s, 3H).

^{13}C NMR (151 MHz, $CDCl_3$): δ 171.31, 165.75, 146.22, 146.02, 130.27, 129.99, 129.53, 128.99, 126.81, 111.59, 60.54, 59.46, 21.91, 21.20, 14.34.

LC-MS [M+H]+ expected: 366.0907, found: 366.0983

5.5.4.4 Example of an HPLC chromatogram

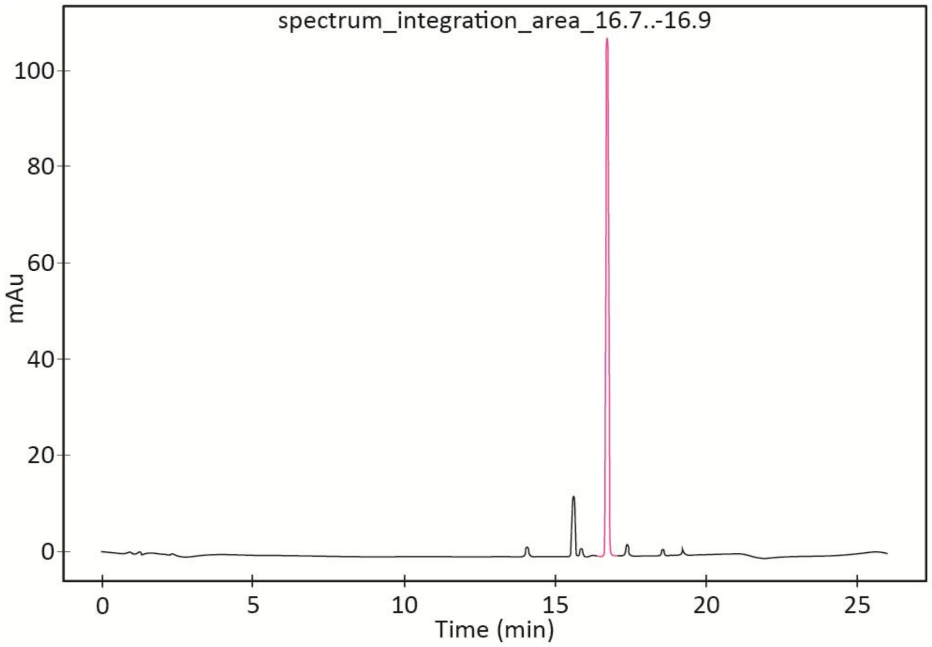

Fig. S35. An HPLC chromatogram obtained after performing optimization for the identified region.

5.5.5 Phloroglucinol, benzylidenemalononitrile and 1,8-bis(dimethylamino)naphthalene reaction

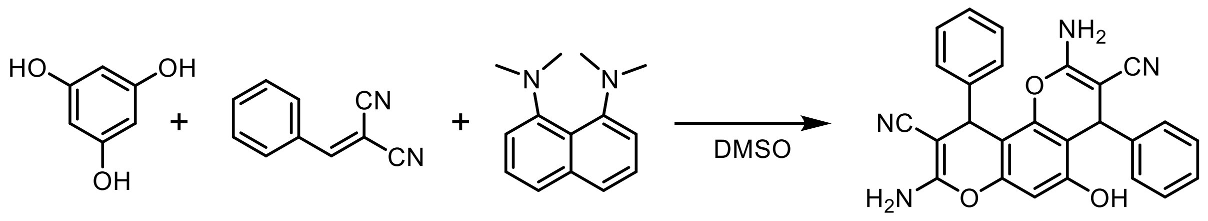

Scheme S9. Reaction between phloroglucinol, benzylidenemalononitrile and 1,8-bis(dimethylamino)naphthalene.

The reaction between phloroglucinol, benzylidenemalononitrile and 1,8-bis(dimethylamino)naphthalene was selected following the methodology described in section 5.5.3. The reaction was subjected to optimization, using HPLC to provide necessary feedback to the decision-making algorithms. All reagents were as 1.0 M solution in DMSO, prepared freshly before a set of 5 consecutive iterations. Naphthalene was used as internal standard for quantitative HPLC analysis.The optimization protocol consisted of two distinct campaigns (see parameters below). The second campaign was initiated after careful analysis of the results of the first one – with increased limits for 1,8-bis(dimethylamino)naphthalene concentration and temperature (see full table of experimental conditions in Appendix D).

5.5.5.1 Optimization parameters

First campaign, 37 reactions:

- benzylidenemalononitrile – variable within 0.2 and 2.0 ml range.
- 1,8-bis(dimethylamino)naphthalene – variable within 0.4 and 4.0 ml range.
- DMSO volume – variable within 0.0 and 6.0 mL range.
- Pre-heat reaction temperature – variable within 25.0 and 160.0 °C range.
- Reaction time – variable within 3600.0 and 28800 seconds.

Second campaign, 13 reactions:

- benzylidenemalononitrile – variable within 0.2 and 4.0 ml range.
- 1,8-bis(dimethylamino)naphthalene – variable within 0.4 and 8.0 ml range.
- DMSO volume – variable within 0.0 and 6.0 mL range.

- Pre-heat reaction temperature – variable within 0.0 and 160.0 °C range.
- Reaction time – variable within 3600.0 and 86400.0 seconds.

The reason for performing two optimizations originated from careful analysis of the data obtained for the first campaign: 6 out of the 37 reactions performed at 25 °C with maximally allowed 1,8-bis(dimethylamino)naphthalene concentration resulted in yields greater than the yield at initial reaction conditions (27%), suggesting that a more "adventurous" approach may lead to interesting results. The second campaign had been initiated with results from previous campaign serving as input and the temperature and constraints allowing for temperatures as low as 0 °C while allowing the 1,8-bis(dimethylamino)naphthalene higher concentrations. During the first campaign the optimization protocol yielded parameters which resulted in a 49% yield, an increase of 22% from the 27% obtained for the initial reaction conditions. However, with the more opened constraints, the explored space has been successfully expanded into unintuitive areas that would not have been a first choice, or a choice at all, for the experimental chemist: in this example the algorithm moved into temperatures not suited for the solvent used (DMSO freezing point is below 19 °C)

5.5.5.2 Description of the automated synthesis

1.0 M phloroglucinol in DMSO solution (variable), 1.0 M benzylidenemalononitrile in DMSO (1 mL), 0.5 M 1,8-bis(dimethylamino)naphthalene in DMSO solution (variable) and DMSO (variable) were added to the reactor. The reaction mixture was stirred for a variable amount of time at a variable temperature. After cooling to room temperature, reference was added to the mixture (1 mL 1M naphthalene) and afterwards a sample (0.5 mL) is withdrawn for the reactor, transferred to an empty flask, diluted to 10.0 mL, and subsequently loaded onto a 5 µL sample loop and injected into the HPLC. The remaining volume of the reaction mixture was discarded, and the platform reset by cleaning all modules with DMSO.

5.5.5.3 Results

^{1}H NMR (600 MHz, DMSO-d_6): δ 10.06 (s, J = 7.2 Hz, 1H), 7.26 (m, 7H), 7.16 (m, 1H), 7.08 (d, J = 7.54 Hz, 2H), 6.98 (s, 2H), 6.71 (s, 2H), 6.28 (s, 1H), 4.64 (s, 1H), 4.45 (s, 1H).

^{13}C NMR (151 MHz, DMSO-d_6): δ 161.07, 159.91, 154.50, 148.78, 146.45, 146.06, 145.14, 129.02, 128.91, 128.74, 127.68, 127.54, 127.16, 127.09, 126.84, 120.95, 120.72, 109.42, 108.23, 103.71, 98.76, 57.37, 44.61, 40.55, 36.99, 36.67.

LC-MS [M+H]+ expected: 435.137, found: 434.900

6 Appendix A

XDL steps and their corresponding parameters that may be subjected for the optimization.

```
{
    "Add": {
        "volume",
        "time",
    },
    "AddSolid": {
        "mass",
    },
    "HeatChill": {
        "time",
        "temp",
    },
    "HeatChillToTemp": {
        "temp",
    },
    "Stir": {
        "time",
    },
    "Wait": {
        "time",
    }
}
```

Fig. S36. XDL steps with optimization parameters.

7 Appendix B

XDL procedures for running dynamic experiments.

```
<?xdl version="0.4.6" ?>

<Synthesis>
  <Hardware>
    <Component
      id="product_flask"
      type="ChemputerFlask" />
    <Component
      id="reactor"
      type="reactor" />
  </Hardware>

  <Reagents>
    <Reagent
      name="benzaldehyde" />
    <Reagent
      name="methanol" />
    <Reagent
      name="benzylamine" />
    <Reagent
      name="fluorobenzoic acid" />
    <Reagent
      name="isocyanide" />
    <Reagent
      name="DCM" />
    <Reagent
      name="reference" />
  </Reagents>

  <Procedure>
    <ResetHandling
      solvent="methanol"
      volume="3 mL"
      repeats="3"
    />
    <Add
      vessel="reactor"
      reagent="benzaldehyde"
      volume="0.2 mL"
      port="0"
      priming_volume="0.2" />
    <ResetHandling
```

```
      solvent="methanol"
      volume="3 mL"
      repeats="3"
    />
    <OptimizeStep
      id="1"
      optimize_properties="{'volume': {'max_value': 5.0, 'min_value': 0.0}
}">
      <Add
        vessel="reactor"
        reagent="methanol"
        volume="1 mL"
        port="0"
        priming_volume="0.2" />
    </OptimizeStep>
    <OptimizeStep
      id="2"
      optimize_properties="{'volume': {'max_value': 1, 'min_value': 0.1}}"
>
      <Add
        vessel="reactor"
        reagent="benzylamine"
        volume="0.22 mL"
        stir="True"
        port="0"
        priming_volume="0.2" />
    </OptimizeStep>
    <ResetHandling
      solvent="methanol"
      volume="3 mL"
      repeats="3"
    />
    <OptimizeStep
      id="3"
      optimize_properties="{'time': {'max_value': 1800.0, 'min_value': 0.0
}}">
      <Stir
        vessel="reactor"
        time="10 mins" />
    </OptimizeStep>
    <OptimizeStep
      id="4"
      optimize_properties="{'volume': {'max_value': 3.0, 'min_value': 0.5}
}">
      <Add
        vessel="reactor"
```

```
        reagent="fluorobenzoic acid"
        volume="1 mL"
        stir="True"
        port="0"
        priming_volume="0.2" />
    </OptimizeStep>
    <ResetHandling
      solvent="methanol"
      volume="3 mL"
      repeats="3"
    />
    <Wait
      time="5 mins" />
    <OptimizeStep
      id="6"
      optimize_properties="{'volume': {'max_value': 1, 'min_value': 0.1}}"
>
      <Add
        vessel="reactor"
        reagent="isocyanide"
        volume="0.23 mL"
        stir="True"
        port="0"
        priming_volume="0.2" />
    </OptimizeStep>
    <ResetHandling
      solvent="methanol"
      volume="3 mL"
      repeats="3"
    />
    <OptimizeStep
      id="7"
      optimize_properties="{'temp': {'max_value': 60.0, 'min_value': 25.0}
}">
      <HeatChillToTemp
        vessel="reactor"
        temp="25.0"
        stir="True"
        purpose="reaction" />
    </OptimizeStep>
    <OptimizeStep
      id="8"
      optimize_properties="{'time': {'max_value': 64800.0, 'min_value': 72
00.0}}">
      <Stir
        vessel="reactor"
```

```
        time="18 hrs" />
    </OptimizeStep>
    <HeatChillToTemp
      vessel="reactor"
      temp="25.0"
      continue_heatchill="False"
      stir="True" />
    <Add
      vessel="reactor"
      reagent="reference"
      volume="5 mL"
      port="0"
      priming_volume="0.2"
      stir="True"/>
    <ResetHandling
      solvent="DCM"
      volume="3 mL"
      repeats="3"
    />
    <FinalAnalysis
      vessel="reactor"
      method="NMR"
      sample_volume="2.5"
      cleaning_solvent="DCM"
      method_props="{'protocol': '1D FLUORINE HDEC', 'protocol_options': {
'centerFrequency': -
110, 'PulselengthScale': 1, 'decouplePower': 0, 'AcquisitionTime': 1.64, '
RepetitionTime': 15, 'PulseAngle': 90, 'Number': 32}}"
      force_shimming="True" />
    <Transfer
      from_vessel="reactor"
      to_vessel="product_flask"
      volume="all"
      from_port="0"
      to_port="0" />
    <ResetHandling
      solvent="DCM"
      volume="3 mL"
      repeats="3"
    />
    <CleanVessel
      vessel="reactor"
      solvent="DCM"
      temp="22.0"
      volume="15 mL" />
    <Shutdown />
```

```
  </Procedure>

</Synthesis>
```

Fig. S37. XDL procedure to run dynamic optimization of the four-component Ugi reaction (section 5.5.1).

```
<?xdl version="0.5.0" ?>

<Synthesis>

  <Hardware>
    <Component
      id="product_flask"
      type="ChemputerFlask" />
    <Component
      id="reactor"
      type="reactor" />
  </Hardware>

  <Reagents>
    <Reagent
      name="TBAF (0.1 M in THF)"
      role="reagent" />
    <Reagent
      name="TBAF (1 M in THF)"
      role="reagent" />
    <Reagent
      name="cyclohexenone"
      role="reagent" />
    <Reagent
      name="trifluoromethyltrimethylsilane"
      role="reagent" />
    <Reagent
      name="acetophenone"
      role="reagent" />
    <Reagent
      name="butyrolactone"
      role="reagent" />
    <Reagent
      name="myrtenal"
      role="reagent" />
    <Reagent
      name="reference"
      role="reagent" />
    <Reagent
      name="THF"
      role="reagent"
      use_for_cleaning="True" />
    <Reagent
      name="DCM"
      role="reagent" />
    <Reagent
```

```
      name="THF_for_cleaning"
      role="reagent"
      use_for_cleaning="True" />
  </Reagents>

  <Procedure>
    <ResetHandling
      solvent="THF" />
    <OptimizeStep
      id="1"
       optimize_properties="{'volume': {'max_value': 0.3, 'min_value':
0.0}}">
      <Add
        vessel="reactor"
        reagent="cyclohexenone"
        volume="0.2 mL"
        aspiration_speed="2.0"
        port="0"
        stir="True"
        priming_volume="0.4 mL" />
    </OptimizeStep>
    <OptimizeStep
      id="3"
       optimize_properties="{'volume': {'max_value': 0.37, 'min_value':
0.0}}">
      <Add
        vessel="reactor"
        reagent="acetophenone"
        volume="0.23 mL"
        aspiration_speed="2.0"
        port="0"
        stir="True"
        priming_volume="0.3 mL" />
    </OptimizeStep>
    <OptimizeStep
      id="5"
       optimize_properties="{'volume': {'max_value': 0.23, 'min_value':
```

```xml
0.0}}">
      <Add
        vessel="reactor"
        reagent="butyrolactone"
        volume="0.16 mL"
        aspiration_speed="2.0"
        port="0"
        stir="True"
        priming_volume="0.3 mL" />
    </OptimizeStep>
    <OptimizeStep
      id="7"
      optimize_properties="{'volume': {'max_value': 0.45, 'min_value':
0.0}}">
      <Add
        vessel="reactor"
        reagent="myrtenal"
        volume="0.3 mL"
        aspiration_speed="2.0"
        port="0"
        stir="True"
        priming_volume="0.3 mL" />
    </OptimizeStep>
    <OptimizeStep
      id="8"
        optimize_properties="{'volume': {'max_value': 10, 'min_value':
0.0}}">
      <Add
        vessel="reactor"
        reagent="THF"
        volume="5 mL"
        port="0"
        stir="True"
        priming_volume="0.2 mL" />
    </OptimizeStep>
    <ResetHandling
      solvent="THF" />
    <OptimizeStep
      id="10"
        optimize_properties="{'temp': {'max_value': 50.0, 'min_value':
```

```
0.0}}">
      <HeatChillToTemp
        vessel="reactor"
        temp="22 °C"
        stir="True"
        stir_speed="450 RPM"
        purpose="reaction" />
    </OptimizeStep>
    <OptimizeStep
      id="11"
      optimize_properties="{'volume': {'max_value': 1.42, 'min_value':
0.36}}">
      <Add
        vessel="reactor"
        reagent="trifluoromethyltrimethylsilane"
        volume="0.9 mL"
        stir="True"
        aspiration_speed="3.0"
        port="0"
        priming_volume="0.2 mL" />
    </OptimizeStep>
    <ResetHandling
      solvent="THF" />
    <OptimizeStep
      id="13"
      optimize_properties="{'volume': {'max_value': 0.8, 'min_value':
0.2}}">
      <Add
        vessel="reactor"
        reagent="TBAF (0.1 M in THF)"
        volume="0.4 mL"
        stir="True"
        aspiration_speed="3.0"
        port="0"
        priming_volume="0.2 mL" />
    </OptimizeStep>
    <ResetHandling
      solvent="THF_for_cleaning" />
    <OptimizeStep
      id="15"
      optimize_properties="{'time': {'max_value': 18000.0, 'min_value':
```

```
300.0}}">
      <Stir
        vessel="reactor"
        time="5 min"
        stir_speed="450 RPM" />
    </OptimizeStep>
    <HeatChillToTemp
      vessel="reactor"
      temp="22 °C"
      stir="True"
      stir_speed="450 RPM"
      purpose="reaction"
      continue_heatchill="False" />
    <OptimizeStep
      id="17"
       optimize_properties="{'volume':  {'max_value':  6.0,  'min_value':
2.0}}">
      <Add
        vessel="reactor"
        reagent="TBAF (1 M in THF)"
        aspiration_speed="3.0"
        volume="6 mL"
        port="0"
        priming_volume="0.2 mL" />
    </OptimizeStep>
    <ResetHandling
      solvent="THF_for_cleaning" />
    <OptimizeStep
      id="19"
      optimize_properties="{'time': {'max_value': 3600.0, 'min_value':
```

```
300.0}}">
      <Stir
        vessel="reactor"
        time="5 min" />
    </OptimizeStep>
    <Add
      vessel="reactor"
      reagent="reference"
      volume="2 mL"
      stir="True"
      port="0"
      aspiration_speed="3.0"
      priming_volume="0.2 mL" />
    <FinalAnalysis
      vessel="reactor"
      method="NMR"
      sample_volume="2.5"
      method_props="{'protocol': '1D FLUORINE HDEC', 'protocol_options':
{'centerFrequency': -85, 'PulselengthScale': 1, 'decouplePower': 0,
'AcquisitionTime': 1.64, 'RepetitionTime': 15, 'PulseAngle': 90,
'Number': 64}}"
      force_shimming="True"
      cleaning_solvent="THF" />
    <Transfer
      from_vessel="reactor"
      to_vessel="product_flask"
      aspiration_speed="3.0"
      volume="all"
      from_port="0"
      to_port="0" />
    <ResetHandling
      solvent="THF_for_cleaning" />
    <CleanVessel
      vessel="reactor"
      solvent="THF_for_cleaning"
      temp="22 °C"
      volume="20 mL" />
  </Procedure>

</Synthesis>
```
Fig. S38. XDL procedure to run dynamic explorative optimization of the trifluoromethylation reaction.

```
<?xdl version="0.5.0" ?>

<Synthesis>

  <Hardware>
    <Component
      id="reactor"
      type="reactor" />
    <Component
      id="waste_3"
      type="ChemputerWaste" />
  </Hardware>

  <Reagents>
    <Reagent
      name="water"
      role="solvent"
      use_for_cleaning="True" />
    <Reagent
      name="NaSO4"
      role="reagent"
      comment="internal standard"/>
    <Reagent
      name="NaHCO3"
      role="activating-agent" />
    <Reagent
      name="MnSO4"
      role="catalyst"
      comment="5 mM" />
    <Reagent
      name="styrene_sulfonate"
      role="reagent"
      comment="0.5 M" />
    <Reagent
      name="30 % hydrogen peroxide solution"
      role="reagent" />
    <Reagent
      name="acid_clean"
      role="solvent"
      use_for_cleaning="True" />
  </Reagents>

  <Procedure>
    <CleanBackbone solvent="water" />
   <Add
        vessel="reactor"
```

```
        reagent="NaSO4"
        volume="2 mL"
        stir="True"
        aspiration_speed="10" />
    <Add
        vessel="reactor"
        reagent="styrene_sulfonate"
        volume="5 mL"
        stir="True"
        aspiration_speed="10" />
    <OptimizeStep
      id="1"
       optimize_properties="{'volume':  {'max_value':  5.0,  'min_value':
0.5}}">
      <Add
        vessel="reactor"
        reagent="NaHCO3"
        volume="3.5 mL"
        stir="True"
        aspiration_speed="10" />
    </OptimizeStep>
    <OptimizeStep
      id="2"
       optimize_properties="{'volume':  {'max_value':  5.0,  'min_value':
```

```
0.5}}">
      <Add
        vessel="reactor"
        reagent="MnSO4"
        volume="5 mL"
        stir="True"
        aspiration_speed="10" />
    </OptimizeStep>
    <ConstrainedStep
      ids="[1, 2, 3]"
      target="18.0"
      parameter="volume">
        <Add
        vessel="reactor"
        reagent="water"
        volume="999 mL"
        stir="True"
        aspiration_speed="10" />
    </ConstrainedStep>
    <StartMonitoring
      pid="monitor"
      method="Raman"
      analysis_delay="60.0" />
    <OptimizeStep
      id="3"
          optimize_properties="{'dispense_speed':  {'max_value':  10.0,
'min_value': 0.0416},
                              'volume': {'max_value': 5.0, 'min_value':
0.5}}">
      <Add
        vessel="reactor"
        reagent="30 % hydrogen peroxide solution"
        volume="5.0 mL"
        stir="True"
        aspiration_speed="10"
        dispense_speed="0.2" />
    </OptimizeStep>
    <OptimizeStep
      id="4"
        optimize_properties="{'time':  {'max_value':  7200,  'min_value':
```

```xml
1800}}">
      <Stir
        vessel="reactor"
        time="1800"
        stir_speed="600" />
    </OptimizeStep>
    <StopMonitoring
      pid="monitor" />
    <FinalAnalysis
      vessel="reactor"
      method="Raman"
      />
    <Transfer
      from_vessel="reactor"
      to_vessel="waste_3"
      volume="60 mL"
      aspiration_speed="10" />
    <Add
      vessel="reactor"
      reagent="acid_clean"
      volume="25 mL"
      stir="True" />
    <Stir
      vessel="reactor"
      time="600"
      stir_speed="600" />
    <Transfer
      from_vessel="reactor"
      to_vessel="waste_3"
      volume="40 mL"
      aspiration_speed="10" />
    <CleanVessel
      vessel="reactor"
      solvent="water"
      volume="25.0 mL"
      temp= "22.0"
      repeats= "1" />
    <Add
      vessel="reactor"
      reagent="water"
      volume="25 mL"
      stir="True" />
    <Stir
      vessel="reactor"
      time="600"
      stir_speed="600" />
```

```
    <Transfer
      from_vessel="reactor"
      to_vessel="waste_3"
      volume="60 mL"
      aspiration_speed="10" />
  </Procedure>

</Synthesis>
```

Fig. S39. XDL procedure to run dynamic optimization of the sodium 4-vinylbenzenesulfonate catalytic oxidation reaction.

```
<?xdl version="0.2.1" ?>

<Synthesis>

  <Hardware>
    <Component
      id="waste_reactors"
      type="ChemputerWaste" />
    <Component
      id="reactor"
      type="reactor" />
  </Hardware>

  <Reagents>
    <Reagent
      name="methanol"
      role="reagent" />
    <Reagent
      name="4-formylbenzonitrile"
      role="reagent" />
    <Reagent
      name="tosmic"
      role="reagent" />
    <Reagent
      name="dbu"
      role="reagent" />
  </Reagents>

  <Procedure>
    <OptimizeStep
      id="1"
       optimize_properties="{'volume': {'max_value': 6.15, 'min_value':
```

```xml
4.1}}">
      <Add
        stir="True"
        volume="5.125 mL"
        vessel="reactor"
        aspiration_speed="5.0"
        priming_volume="1 mL"
        reagent="tosmic" />
    </OptimizeStep>
    <Add
      stir="True"
      volume="4.1 mL"
      vessel="reactor"
      aspiration_speed="5.0"
      priming_volume="1 mL"
      reagent="4-formylbenzonitrile" />
    <OptimizeStep
      id="2"
      optimize_properties="{'volume': {'max_value': 0.308, 'min_value':
0.154}}">
      <Add
        stir="True"
        volume="0.231 mL"
        vessel="reactor"
        aspiration_speed="1.0"
        priming_volume="1 mL"
        reagent="dbu" />
    </OptimizeStep>
    <Add
      stir="True"
      volume="5.0 mL"
      vessel="reactor"
      aspiration_speed="5.0"
      priming_volume="1 mL"
      reagent="methanol" />
    <OptimizeStep
      id="3"
        optimize_properties="{'temp': {'max_value': 75.0, 'min_value':
```

```
25.0}}">
      <HeatChillToTemp
        vessel="reactor"
        temp="60.0"
        stir="True"
        purpose="reaction" />
    </OptimizeStep>
    <OptimizeStep
      id="4"
       optimize_properties="{'time':  {'max_value':  10800,  'min_value':
1800}}">
      <Stir
        time="1800"
        vessel="reactor" />
    </OptimizeStep>
    <HeatChillToTemp
      vessel="reactor"
      temp="25.0"
      continue_heatchill="False"
      stir="True" />
    <FinalAnalysis
      vessel="reactor"
      method="HPLC"
                               method_props="{'run_method':'AH_default',
```

```
'cleaning_method':'AH_cleaning', 'channel':'B'}"
      sample_volume="0.5"
      dilution_vessel="dilution"
      dilution_volume="19.5"
      dilution_solvent="acetonitrile"
      cleaning_solvent="methanol" />
    <Transfer
      from_vessel="reactor"
      to_vessel="waste_reactors"
      volume="30 mL" />
    <CleanVessel
      vessel="reactor"
      solvent="methanol"
      volume="15 mL"
      temp= "22.0"
      repeats= "3" />

  </Procedure>

</Synthesis>
```

Fig. S40. XDL procedure to run dynamic optimization of the Van Leusen oxazole synthesis.

```
<?xdl version="1.0.0" ?>
<XDL>

<Synthesis>

  <Hardware>
    <Component
      id="product_flask"
      type="ChemputerFlask" />
    <Component
      id="reactor"
      type="ChemputerReactor" />
    <Component
      id="sample_flask"
      type="ChemputerFlask" />

  </Hardware>

  <Reagents>
    <Reagent
      name="nitrile_in_DMSO"
      role="reagent" />
    <Reagent
      name="isocyanide_in_DMSO"
      role="reagent" />
```

```
    <Reagent
      name="DMSO"
      role="reagent"
      use_for_cleaning="True" />
    <Reagent
      name="acetonitrile"
      role="reagent"
      use_for_cleaning="True" />
    <Reagent
      name="reference"
      role="reagent" />
  </Reagents>

  <Procedure>
    <ResetHandling
      solvent="DMSO" />

    <Add
      vessel="reactor"
      reagent="nitrile_in_DMSO"
      volume="1 mL"
      aspiration_speed="2.0"
      port="0"
      stir="True"
      priming_volume="0.2 mL" />

    <OptimizeStep
      id="1"
      optimize_properties="{'volume': {'max_value': 2, 'min_value': 0.2}}">
    <Add
      vessel="reactor"
      reagent="isocyanide_in_DMSO"
      volume="1 mL"
      aspiration_speed="2.0"
      port="0"
      stir="True"
      priming_volume="0.2 mL"/>
    </OptimizeStep>

    <OptimizeStep
      id="2"
      optimize_properties="{'volume': {'max_value': 5, 'min_value': 0.0}}">
    <Add
      vessel="reactor"
      reagent="DMSO"
      volume="1 mL"
      aspiration_speed="2.0"
      port="0"
```

```
      stir="True"
      priming_volume="0.2 mL"/>
    </OptimizeStep>

    <OptimizeStep
      id="3"
      optimize_properties="{'temp': {'max_value': 160, 'min_value': 30}}">
      <HeatChillToTemp
        vessel="reactor"
        temp="100 °C"
        stir="True"
        stir_speed="450 RPM"
        purpose="reaction" />
     </OptimizeStep>

    <OptimizeStep
      id="4"
      optimize_properties="{'time': {'max_value': 28800, 'min_value': 3600
}}">
      <Wait
      time="4 h" />
    </OptimizeStep>

    <HeatChillToTemp
        vessel="reactor"
        temp="30 °C"
        stir="True"
        stir_speed="450 RPM"
        purpose="reaction"
        active="True"
        continue_heatchill="False" />

    <Add
      vessel="reactor"
      reagent="reference"
      volume="1 mL"
      aspiration_speed="2.0"
      port="0"
      stir="True"
      priming_volume="0.2 mL"/>

    <FinalAnalysis
      vessel="reactor"
      method="HPLC"
      method_props="{'run_method': '26min_Optim', 'cleaning_method':
'AIL_cleaning', 'channel': 'A'}"
      sample_volume="0.5"
      dilution_volume="9.5"
```

```
      dilution_solvent="acetonitrile"
      cleaning_solvent="acetonitrile" />
    <Transfer
      from_vessel="reactor"
      to_vessel="product_flask"
      aspiration_speed="3.0"
      volume="all"
      from_port="0"
      to_port="0" />
    <ResetHandling
      solvent="DMSO" />
    <CleanVessel
      vessel="reactor"
      solvent="DMSO"
      volume="20 mL" />
  </Procedure>

</Synthesis>
</XDL>
```

Fig. S40. XDL procedure to run dynamic optimization of the tosMIC reaction.

```
<?xdl version="1.0.0" ?>
<XDL>

<Synthesis>

  <Hardware>
    <Component
      id="product_flask"
      type="ChemputerFlask" />
    <Component
      id="reactor"
      type="ChemputerReactor" />
    <Component
      id="sample_flask"
      type="ChemputerFlask" />

  </Hardware>

  <Reagents>
    <Reagent
      name="phloroglucinol_in_DMSO"
      role="reagent" />
    <Reagent
      name="nitrile_in_DMSO"
      role="reagent" />
    <Reagent
      name="sponge_in_DMSO"
```

```xml
      role="reagent" />
    <Reagent
      name="DMSO"
      role="reagent"
      use_for_cleaning="True" />
    <Reagent
      name="acetonitrile"
      role="reagent"
      use_for_cleaning="True" />
    <Reagent
      name="reference"
      role="reagent" />
  </Reagents>

  <Procedure>
    <ResetHandling
      solvent="DMSO" />

    <Add
      vessel="reactor"
      reagent="phloroglucinol_in_DMSO"
      volume="1 mL"
      aspiration_speed="2.0"
      port="0"
      stir="True"
      priming_volume="0.2 mL" />

    <OptimizeStep
      id="1"
      optimize_properties="{'volume': {'max_value': 4, 'min_value': 0.2}}">
    <Add
      vessel="reactor"
      reagent="nitrile_in_DMSO"
      volume="4 mL"
      aspiration_speed="2.0"
      port="0"
      stir="True"
      priming_volume="0.2 mL"/>
    </OptimizeStep>

    <OptimizeStep
      id="2"
      optimize_properties="{'volume': {'max_value': 8, 'min_value': 0.4}}">
    <Add
      vessel="reactor"
      reagent="sponge_in_DMSO"
      volume="8 mL"
      aspiration_speed="2.0"
```

```
      port="0"
      stir="True"
      priming_volume="0.2 mL"/>
    </OptimizeStep>

    <OptimizeStep
      id="3"
      optimize_properties="{'volume': {'max_value': 6, 'min_value': 0.0}}">
    <Add
      vessel="reactor"
      reagent="DMSO"
      volume="0 mL"
      aspiration_speed="2.0"
      port="0"
      stir="True"
      priming_volume="0.2 mL"/>
    </OptimizeStep>

    <OptimizeStep
      id="4"
      optimize_properties="{'temp': {'max_value': 160, 'min_value': 0}}">
      <HeatChillToTemp
        vessel="reactor"
        temp="0 °C"
        stir="True"
        stir_speed="450 RPM"
        purpose="reaction" />
     </OptimizeStep>

    <OptimizeStep
      id="5"
      optimize_properties="{'time': {'max_value': 86400, 'min_value': 3600
}}">
      <Wait
      time="4 h" />
    </OptimizeStep>

    <HeatChillToTemp
        vessel="reactor"
        temp="25 °C"
        stir="True"
        stir_speed="450 RPM"
        purpose="reaction"
        active="True"
        continue_heatchill="False" />

    <Add
      vessel="reactor"
```

```
      reagent="reference"
      volume="1 mL"
      aspiration_speed="2.0"
      port="0"
      stir="True"
      priming_volume="0.2 mL"/>

    <FinalAnalysis
      vessel="reactor"
      method="HPLC"
      method_props="{'run_method': '26min_Optim', 'cleaning_method':
'AIL_cleaning', 'channel': 'A'}"
      sample_volume="0.5"
      dilution_volume="9.5"
      dilution_solvent="MeCN"
      cleaning_solvent="MeCN" />
    <Transfer
      from_vessel="reactor"
      to_vessel="product_flask"
      aspiration_speed="3.0"
      volume="all"
      from_port="0"
      to_port="0" />
    <ResetHandling
      solvent="DMSO" />
    <CleanVessel
      vessel="reactor"
      solvent="DMSO"
      volume="20 mL" />
  </Procedure>

</Synthesis>

</XDL>
```

Fig. S41. XDL procedure to run dynamic optimization of the phloroglucinol reaction.

8 Appendix C

Configuration files for running reaction optimization.

```
{
    "max_iterations": 5.0,
    "target": {
        "spectrum_peak-area_-114.3": "inf"
    },
    "algorithm": {
        "name": "random",
        "random_state": 42
    },
    "reference": -119.2
}
```

Fig. S42. Configuration file to run four-component Ugi reaction optimization during initial random parameter screening.

```
{
    "max_iterations": 5.0,
    "target": {
        "spectrum_peak-area_-114.3": "inf"
    },
    "algorithm": {
        "name": "smbo",
        "base_estimator": "GP",
        "n_initial_points": 2,
        "acq_func": "PI",
        "acq_func_kwargs": {},
        "random_state": 42
    },
    "reference": -119.2
}
```

Fig. S43. Configuration file to run four-component Ugi reaction optimization using SMBO algorithm with acquisition function favouring exploration.

```
{
    "max_iterations": 5.0,
    "target": {
        "spectrum_peak-area_-114.3": "inf"
    },
    "algorithm": {
        "name": "smbo",
        "n_initial_points": 2,
        "acq_func": "gp_hedge",
        "random_state": 42
    },
```

```
    "reference": -119.2
}
```

Fig. S44. Configuration file to run four-component Ugi reaction optimization using SMBO algorithm with acquisition function probabilistically chosen between "LCB", "EI" or "PI" (referred to as "balanced" strategy, see details in the scikit-optimize API reference[7]).

```
{
    "max_iterations": 3.0,
    "target": {
        "spectrum_peak-area_-114.3": "inf"
    },
    "algorithm": {
        "name": "smbo",
        "n_initial_points": 2,
        "acq_func": "LCB",
        "acq_func_kwargs": {"kappa": 0.1},
        "random_state": 42
    },
    "reference": -119.2
}
```

Fig. S45. Configuration file to run four-component Ugi reaction optimization using SMBO algorithm with acquisition function favouring exploitation.

```
{
    "max_iterations": 25.0,
    "target": {
        "multie_purity-area_14": "inf"
    },
    "algorithm": {
        "name": "SNOBFIT",
    },
    "reference": 20.1,
    "batch_size": 2
}
```

Fig. S42. Configuration file to run Van Leusen oxazole synthesis using SNOBFIT algorithm via the Summit server framework with 2 parallel reactors.

```
{
    "max_iterations": 10,
    "target": {
        "novelty": Infinity
    },
    "algorithm": {
        "name": "random",
        "random_state": 43
    },
    "reference": -113.15,
    "batch_size": 1
}
```

Fig. S43. Configuration file to run explorative optimization of trifluoromethylation reaction during initial random screening experiments.

```
{
    "max_iterations": 11,
    "target": {
        "novelty": Infinity
    },
    "algorithm": {
        "name": "smbo",
        "base_estimator": "GP",
        "n_initial_points": 15,
        "initial_point_generator": "grid",
        "acq_func": "LCB",
        "acq_func_kwargs": {"kappa": 10000}
    },
    "reference": -113.15,
    "batch_size": 1
}
```

Fig. S44. Configuration file to run explorative optimization of the trifluoromethylation reaction during second phase – exploration of the parameter space.

```
{
    "max_iterations": 6,
    "target": {
        "novelty": Infinity
    },
    "algorithm": {
        "name": "smbo",
        "base_estimator": "GP",
        "n_initial_points": 1,
        "initial_point_generator": "grid",
        "acq_func": "LCB",
        "acq_func_kwargs": {"kappa": 0.001}
```

```
    },
    "reference": -113.15,
    "batch_size": 1
}
```

Fig. S45. Configuration file to run explorative optimization of the trifluoromethylation reaction during second phase – exploitation of the parameter space to maximize the `Novelty` parameter.

```
{
    "max_iterations": 5,
    "target": {
        "spectrum_integration-area_-75.623..-76.08": Infinity
    },
    "algorithm": {
        "name": "smbo",
        "base_estimator": "GP",
        "acq_func": "LCB",
        "acq_func_kwargs": {
            "kappa": 0.001
        },
        "random_state": 42
    },
    "reference": -113.15,
    "constraints": [
        "-73.319..-73.533",
        "-74.317..-74.41",
        "-75.304..-75.55",
        "-76.629..-76.734",
        "-78.4..-78.868",
        "-79.992..-80.412",
        "-81.12..-81.698",
        "-106.519..-106.721",
        "-113.071..-113.225",
        "-116.401..-116.911"
    ],
    "batch_size": 1
}
```

Fig. S50. An example of the configuration file to run the optimization of the individual product of the trifluoromethylation reaction. Parameter `constraints` lists all other identified products that should be considered, when calculating the result.

```
{
    "max_iterations": 12,
    "target": {
        "spectrum_integration-area_16.7..16.9": Infinity
    },
    "algorithm": {
        "name": "smbo",
        "acq_func": "gp_hedge",
        "n_initial_points": 0
    },
    "reference": 18.2,
    "constraints": [
        "14.0..14.4",
        "15.5..15.7"
    ],
    "batch_size": 1,
    "control": {
        "n_runs": 0
    }
}
```

Fig. S51. An example of the configuration file to run the optimization of the tosMIC reaction.

```
{
    "max_iterations": 12,
    "target": {
        "spectrum_integration_area_15.0..15.2": Infinity
    },
    "algorithm": {
        "name": "smbo",
        "acq_func": "gp_hedge",
        "n_initial_points": 0
    },
    "reference": 18.2,
    "batch_size": 1,
    "control": {
        "n_runs": 0
    }
}
```

Fig. S52. An example of the configuration file to run the optimization of phloroglucinol reaction.

```json
{
    "max_iterations": 40,
    "target": {
        "spectrum_peak-area_1633": "inf"
    },
    "algorithm": {
        "name": "Phoenics"
    },
    "reference": 2261,
    "batch_size": 1
}
```
Fig. S53.Configuration file for the optimization of the Sodium 4-vinylbenzenesulfonate catalytic oxidation using Phoenics optimization algorithm.

9 Appendix D

Full results table for reaction optimization.

Iteration	Add_3-volume	Add_4-volume	Stir_6-time	Add_7-volume	Add_10-volume	HeatChillToTemp_12-temp	Stir_13-time	spectrum_peak-area_-114.3	Strategy
1	1	0.22	600	1	0.23	20	64800	0.3997	initial
2	4.89	0.41	732.23	1.01	0.77	20	20999.27	0.3513	random
3	0.63	0.49	1226.77	0.93	0.69	20	11612.19	0.3596	random
4	0.08	0.23	1501.71	2.29	0.48	50.34	26902.12	0.2097	random
5	2.17	0.44	1394.91	0.77	0.96	27.24	62775.59	0.2513	random
6	1.82	0.37	1583.79	2.94	0.74	28.65	44660.21	0.3332	random
7	2.17	0.43	1182.48	0.56	0.39	28.83	32768.74	0.1688	exploration
8	1.68	0.98	642.7	2.15	0.81	31.01	42750.15	0.2557	exploration
9	1.34	0.53	179.28	2.28	0.34	37.38	55827.88	0.4	exploration
10	5	1	1800	0.5	1	25	7200	0	exploration
11	4.33	0.67	1453.24	0.51	0.43	25.89	54755.64	0	exploration
12	0.53	0.37	139.83	1.56	0.11	42.38	13629.76	0.2327	exploration
13	0.87	0.25	1342.07	2.22	0.3	56.59	16607.29	0.2652	exploration
14	5	0.1	0	3	0.1	60	64800	0	exploration
15	1.21	0.34	226.62	1.66	0.46	52.66	13751.27	0.3467	exploration
16	3.18	0.39	1171.8	2.18	0.26	52.13	52989.08	0.3723	exploration
17	0.45	0.32	534.94	0.96	0.57	27.51	60105.29	0.2906	exploration
18	1	0.41	1478.19	0.93	0.47	49.16	27644.73	0.2656	exploration
19	5	0.26	438.42	0.53	1	60	49493.84	0.2134	exploration
20	3.51	0.42	28.88	1.87	1	60	16979.32	0.5124	exploration
21	0	0.97	184.89	2.65	0.79	25	54012.88	0.2309	exploitation[a]
22	0	1	0	3	0.1	25	64800	0.2011	exploitation[a]
23	0	1	0	3	0.1	25	64800	0.2048	exploitation[a]

24	5	0.29	1044.02	1.96	0.46	52.69	52023.27	0.4144	balance
25	0	0.15	1137.27	1.1	0.23	25	40311.72	0	balance
26	5	0.44	1484.39	2.12	1	50.48	11831.81	0.5019	balance
27	5	0.46	967.27	3	0.1	26.78	8526.74	0.2113	balance
28	5	0.49	1138.17	1.76	1	57.38	31964.02	0.5035	balance
29	5	0.45	920.79	1.84	1	44.33	22422.12	0.5059	exploitation
30	5	0.43	657.43	1.94	1	55.74	16045.76	0.5506	exploitation
31	4.33	0.42	1245.2	1.89	1	41.11	15034.47	0.5408	exploitation

Table S6. Results of the four-component Ugi reaction optimization. Objective ("spectrum_peak-area_-114.3") refers to the integration area of the corresponding peak in the spectrum, where 0 indicates no peak found. [a] the objective was falsely assigned to the side product peak, leading to incorrect results during "exploitation" approach.

Iteration	Add_1-volume	Add_2-volume	Add_3-volume	Add_4-volume	Add_5-volume	HeatChillToTemp_7-temp	Add_8-volume	Add_10-volume	Stir_12-time	Add_14-volume	Stir_16-time
0	0.2	0.02	0	0.38	5.87	11.24	1.16	0.36	7733.61	3.8	3452.54
1	0.18	0.35	0.23	0.02	1.19	38.06	0.49	0.41	5610.34	4.2	467.05
2	0.14	0.21	0.03	0.15	5.05	15.2	0.8	0.45	1951.66	4.35	544.38
3	0.17	0.03	0.12	0.38	2.89	4.14	0.7	0.45	11135.33	2.61	313.07
4	0.075	0.0925	0.115	0.225	2.5	12.5	0.89	0.35	9150	3	1950
5	0.075	0.2775	0.1725	0.225	2.5	12.5	1.155	0.65	13575	3	2775
6	0.075	0.185	0.1725	0.1125	2.5	37.5	1.155	0.65	4725	3	2775
7	0.075	0.185	0.0575	0.3375	5	37.5	0.89	0.65	4725	3	1125
8	0.15	0.2775	0.115	0.1125	5	25	0.89	0.35	13575	5	1125
9	0.075	0.0925	0.115	0.225	2.5	37.5	0.89	0.35	13575	5	1950
10	0.15	0.0925	0.115	0.1125	2.5	37.5	0.625	0.65	13575	3	1125
11	0.225	0.185	0.0575	0.225	2.5	37.5	1.155	0.5	13575	5	1125
12	0.15	0.0925	0.115	0.3375	5	12.5	0.89	0.65	4725	5	1125
13	0.225	0.0925	0.1725	0.1125	2.5	37.5	1.155	0.5	13575	4	1950
14	0.075	0.185	0.0575	0.3375	5	25	0.625	0.5	4725	3	1950
15	0	0	0.2033	0.45	10	0	1.42	0.8	18000	6	3600
16	0.3	0.37	0	0.45	0	50	0.36	0.2	18000	6	3600
17	0	0.37	0.23	0	10	0	1.42	0.2	300	6	300
18	0.3	0.37	0	0	10	50	0.36	0.8	300	2	3600
19	0.3	0	0	0	10	0	1.42	0.8	300	6	300
20	0	0.37	0	0.45	0	50	0.36	0.8	18000	2	3600
21	0.3	0.2361	0	0.45	10	0	0.36	0.8	300	6	3600
22	0.278	0.1257	0.23	0.4485	10	46.4547	1.42	0.2	17033.14	2.3497	3600
23	0.3	0	0	0	0	0	1.42	0.8	18000	6	3600
24	0	0.0073	0.23	0.4318	7.3884	50	0.6393	0.7332	300	2	3600
25	0.3	0.37	0.23	0.45	0	50	0.36	0.2	18000	6	300
26	0.2372	0.277	0.1004	0.3637	9.6538	8.611	0.8656	0.5804	6231.506	5.307	2368.209

27	0.0038	0.3542	0.23	0.0075	7.5622	0	1.4027	0.7963	18000	2.0344	300
28	0.2901	0.0339	0.0266	0	3.8379	50	1.1133	0.7158	17219.21	4.8051	3600
29	0.0565	0.3554	0.0487	0.0218	8.0995	27.68	0.8693	0.4565	4982.456	5.0804	2571.033
30	0.2637	0.1174	0.1701	0.3444	8.8769	1.9052	1.3426	0.3086	4411.009	5.6418	336.2277
31	0.3	0	0.0126	0	0	0	1.42	0.2	18000	6	3247.71
32	0.0051	0.3364	0.2221	0.0035	7.5653	0	1.3032	0.7983	18000	2.6423	300
33	0	0.2214	0.23	0	10	2.689	1.42	0.8	9738.93	2	300
34	0.1829	0.1246	0.0737	0.0075	7.5814	13.6651	1.4027	0.7963	18000	2.4345	300.0008
35	0.035	0.2975	0.1758	0.1265	7.6666	35.9542	1.0589	0.7999	18000	2.0339	669.7364
36	0.0924	0.3542	0.2146	0.3076	7.5021	0	0.7836	0.793	13514.36	4.2811	414.8101
37	0.2113	0.114	0.2146	0.3826	7.5027	24.3047	0.7836	0.7932	7280.613	4.2779	1503.354
38	0.1327	0.3473	0.2152	0.3546	7.5452	44.3654	0.8886	0.7851	5177.951	4.3175	420.7506
39	0.3	0.0002	0	0	0.0006	0	1.42	0.8	18000	6	3600
40	0.1583	0.3466	0.1638	0.0399	7.5592	34.7032	1.4027	0.6237	11739.65	3.3397	300
41	0.2311	0.1916	0.0185	0.107	0	0	1.0839	0.8	17916.68	4.6267	2714.998
42	0.2027	0.2305	0.0414	0.0971	0.6613	0	1.0743	0.8	17829.98	4.1059	2227.308
43	0.3	0	0	0	4.490189	39.77515	1.02661	0.58786	7327.404	3.866757	2270.768
44	0.3	0	0	0	9.948695	49.10743	1.016392	0.330502	300	2	537.9938
45	0.3	0	0	0	3.644377	27.88199	0.955637	0.567561	300	2.603835	300
46	0.3	0	0	0	3.010489	9.103317	0.922228	0.588549	300	2	300
47	0.3	0	0	0	2.854585	32.1703	0.917143	0.629172	300	2.96925	300
48	0	0.37	0.23	0	0	50	0.54765	0.2	18000	4.252653	3600
49	0	0.37	0.23	0	0	50	0.36	0.2	18000	3.443402	3600
50	0	0.37	0.23	0	0	50	0.541411	0.2	18000	4.102209	3600
51	0	0.37	0.23	0	0	50	0.568638	0.2	18000	3.191623	3600
52	0	0.37	0.23	0	1.112906	50	0.589825	0.2	18000	2	3600
53	0.059886	0	0.05998	0.393794	0	8.496476	0.423968	0.2	8675.748	2	300
54	0.2367	0.01234	0.080616	0.388762	0	1.35394	0.660831	0.281183	18000	2	300
55	0	0	0.197377	0.413064	8.380848	29.25047	0.36	0.776153	11971.66	2	945.5722

56	0	0	0.199944	0.418024	7.74733	15.00305	0.36	0.702736	11943.13	2.585061	2603.807
57	0.024245	0	0.23	0.432863	1.839167	0	0.532426	0.730457	11791.81	3.29267	907.0798
58	0.3	0.086836	0	0	2.372057	27.3889	1.327589	0.574897	4396.9	3.804945	1045.072
59	0.3	0.108147	0	0	3.361527	30.58552	0.457217	0.676836	10767.93	4.252617	2070.023
60	0.3	0.232246	0	0	4.812268	39.0591	1.014403	0.628306	5125.857	3.820014	1518.177
61	0.3	0	0	0	1.627027	18.95629	1.086774	0.521459	6580.949	4.063436	1890.513
62	0.3	0	0	0	5.666691	44.75601	1.114701	0.51893	14128.56	4.150016	1269.458
63	0.3	0	0	0	0.558368	0	0.702877	0.2	300	2	300
64	0.3	0	0	0	1.529958	0	0.749839	0.2	300	2	300
65	0.3	0	0	0	0	29.16563	0.611402	0.8	300	2	300
66	0.3	0	0	0	0.375438	20.24044	0.717494	0.8	300	2	300
67	0.3	0	0	0	0	14.77393	0.668792	0.8	300	2	300
68	0.3	0	0	0	6.978	36.2612	1.1331	0.5781	13024.99	4.2031	1288.411
69	0.1788	0.1533	0	0.0906	6.9438	41.2809	1.0869	0.2351	13491.75	2.3058	1147.581
70	0.099411	0.308388	0	0.237783	6.896688	36.37782	1.043806	0.2	12127.08	5.037321	1449.423
71	0.178127	0.154098	0	0.142892	6.91627	44.09105	0.761888	0.233305	13629.27	2	1487.288
72	0.240633	0.055215	0.110086	0.014404	8.732324	46.07651	1.144411	0.225953	13467.95	4.443958	1490.177
73	0.261994	0.3357	0.181466	0.009672	8.788054	46.67213	1.184446	0.295729	13443.88	4.214546	2061.496
74	0.092298	0.205309	0.118327	0.031974	4.814851	45.09937	1.226145	0.225269	13454.57	5.564882	376.7512
75	0.3	0.37	0.23	0.45	0	50	0.36	0.2	300	6	300
76	0.3	0.37	0.23	0.45	10	18.45753	0.36	0.2	18000	6	300
77	0.3	0.37	0.23	0.45	10	0	0.36	0.8	18000	6	300
78	0.3	0.37	0.23	0.45	10	0	0.36	0.8	18000	6	300
79	0.3	0.37	0.23	0.45	10	0	0.36	0.8	18000	6	300
80	0.101993	0.104922	0.23	0.084776	7.831853	30.42868	0.36	0.221733	1882.467	2	2503.105
81	0	0	0.198333	0.45	8.327648	0	0.36	0.607982	300	3.460318	3600
82	0.026552	0	0.202983	0.45	10	12.79337	0.36	0.50523	9821.36	2.017791	3600
83	0	0	0.23	0.45	10	1.62732	0.36	0.2	9805.984	2.955296	3600

Table S7. Reaction parameters used in the explorative optimization of the product space for trifluoromethylation reaction (see Section 5.5.2).

Iteration	-73.319<br>-73.533[a]	-74.317<br>-74.41	-75.304<br>-75.55	-75.623<br>-76.08	-76.629<br>-76.734	-78.4<br>-78.868	-79.992<br>-80.412	-81.12<br>-81.698	-106.519<br>-106.721	-113.071<br>-113.225	-116.401<br>-116.911
0	0.026931	0.000807	0.002971	0.445805	0.007102	0.302055	0.04809	0.363223	0.001185	0.102573	0.016347
1	0.00094	0.009541	0.004244	0.046386	0.000931	0.106624	0.827693	0.038715	0.076143	0.184034	0.146073
2	0.027752	0.002456	0.012855	0.148416	0.00558	0.192891	0.355283	0.249336	0.005203	0.134312	0.082326
3	0.02013	0.005185	0.016818	0.939715	0.000999	0.14083	0.073294	0.152789	0.000727	0.154533	0.014469
4	0.01252	0.000731	0.026094	0.336337	0.002723	0.495453	0.12161	0.115449	0.001995	0.163276	0.021963
5	0.004393	0.000453	0.038487	0.2472	0.002989	0.287555	0.492673	0.033372	0.003742	0.111706	0.071846
6	0.0074	0.00021	0.034078	0.104854	8.50E-06	0.590698	0.329655	0.077302	0.001375	0.127612	0.06271
7	0.012679	0.000368	0.005568	0.475924	0.000104	0.246552	0.268785	0.083348	0.005704	0.126322	0.057775
8	0.013562	0.001987	0.01331	0.092497	0.007747	0.288049	0.520699	0.116637	0.005939	0.124066	0.101779
9	0.00567	0.005399	0.026825	0.321798	0.006014	0.54744	0.128593	0.072311	0.002991	0.169844	0.031057
10	0.025828	0.002395	0.022695	0.165243	0.004296	0.242517	0.191296	0.289377	0.002649	0.232915	0.033493
11	0.031351	0.000201	0.007414	0.212891	0.023131	0.262222	0.247272	0.260417	0.003547	0.105051	0.055635
12	0.015538	0.001017	0.016914	0.499698	0.003459	0.328254	0.098611	0.187059	0.000466	0.132692	0.018659
13	0.03343	0.002213	0.034397	0.096894	0.017261	0.573336	0.122443	0.259653	0.002467	0.137017	0.024901
14	0.007189	0.005232	0.001982	0.602139	0.000686	0.122655	0.31748	0.057719	0.007518	0.153555	0.068131
15	0.002656	0.005772	0.041705	0.518892	0.001651	0.97603	0.010474	0.00232	0.002556	0.103041	0.005756
16	0.003343	0.001196	0.004481	0.322733	0.002605	0.021159	0.392804	0.004489	0.163685	0.265423	0.095051
17	0.000374	0.000725	0.009511	0.003626	0.000316	1.065229	0.449046	0.004027	0.003589	0.075005	0.089932
18	0.004735	0.001957	0.000957	0.0074	0.001835	0.225859	0.715106	0.081718	0.095298	0.253707	0.01739
19	0.036852	0.000714	0.00096	0.000477	0.031314	1.05318	0.000225	0.502466	0.00103	0.086546	0.003675
20	0.000762	0.000501	0.001097	0.34051	0.000765	0.013483	0.489516	0.006217	0.145487	0.215159	0.099707
21	0.002724	0.003487	0.006295	0.536713	0.001728	0.197231	0.173985	0.013999	0.109195	0.26262	0.003392
22	0.008404	0.004715	0.009333	0.506539	0.001758	0.521677	0.125638	0.079668	0.004264	0.090206	0.02514
23	0.016156	0.000203	0.002818	0.010638	0.019702	0.686884	0.001575	0.677108	0.000885	0.153337	0.004929
24	0.001889	0.036985	0.004354	0.904579	0.00129	0.285038	0.102332	0.003641	0.006038	0.1728	0.010368
25	0.004209	0.0003	0.003218	0.244419	0.001542	0.039368	0.350685	0.01863	0.238494	0.264902	0.082902
26	0.007477	0.014467	0.004675	0.437983	0.00155	0.104838	0.399779	0.110493	0.014178	0.10433	0.085885

27	0.003568	0.001095	0.044824	0.038362	0.000808	0.646166	0.707454	0.004511	0.006673	0.104355	0.002227
28	0.058844	0.001702	0.008197	0.000445	0.043134	0.456031	0.076565	0.59197	0.000574	0.136182	0.016468
29	0.007545	0.000762	0.005213	0.028837	0.002882	0.266123	0.791957	0.066572	0.010283	0.115372	0.146119
30	0.016108	0.00952	0.014236	0.303131	0.007598	0.667077	0.107555	0.136849	0.001235	0.084551	0.024272
31	0.024434	0.000646	0.002751	0.001393	0.025522	0.561601	0.003004	0.74639	0.000843	0.180919	0.002426
32	0.006302	0.001021	0.034352	0.042783	0.002301	0.506954	0.557055	0.048849	0.003437	0.090872	0.097346
33	0.000227	0.0023	0.04659	0.00023	0.00057	1.312141	0.35125	0.002824	0.001182	0.100279	0.030483
34	0.018894	0.00079	0.010694	0.04511	0.003708	0.739151	0.163782	0.294392	0.001053	0.109913	0.0309
35	0.004242	0.000864	0.014121	0.090767	0.001243	0.391997	0.598554	0.035119	0.007937	0.11858	0.102145
36	0.002109	0.023906	0.006266	0.321493	0.000953	0.104398	0.602624	0.020991	0.024035	0.119229	0.114798
37	0.008268	0.045214	0.015427	0.600199	0.00146	0.212933	0.154772	0.094202	0.006551	0.142381	0.030925
38	0.002644	0.022617	0.004289	0.358655	0.00165	0.139952	0.501183	0.029184	0.031227	0.110372	0.099726
39	0.018839	0.000447	0.004291	0.011991	0.02022	0.60678	0.0215	0.722576	0.000984	0.139282	0.00374
40	0.015408	0.001338	0.020358	0.035429	0.000152	0.471112	0.502055	0.112417	0.006878	0.083007	0.099082
41	0.035675	0.000191	0.00731	0.034192	0.034565	0.218196	0.217735	0.589328	0.001138	0.131298	0.047909
42	0.030842	0.000538	0.01364	0.079706	0.012073	0.14267	0.369772	0.41443	0.002504	0.119162	0.079393
43	0.0713	0.001826	0.001032	0.002241	0.057747	0.355026	0.009599	0.868988	0.000103	0.154082	0.00368
44	0.0459	0.002067	0.000817	0.003452	0.001438	0.962873	0.000784	0.516171	0.002633	0.126997	0.001295
45	0.0793	0.001333	0.001946	0.003414	0.008486	0.355658	0.003712	1.071054	0.000499	0.1432	0.002514
46	0.058	0.00041	0.003115	0.001431	0.000883	0.288345	0.005747	1.367022	0.000602	0.149348	0.001719
47	0.088	5.36E-05	0.001612	0.001226	0.032118	0.29471	0.011188	1.088354	0.000135	0.139541	0.002657
48	0.001362	0.002587	0.006281	0.000543	0.003614	0.070259	1.195571	0.00788	0.044072	0.202649	0.186179
49	0.002936	0.001261	0.003716	0.000845	0.000709	0.015793	1.068248	0.005618	0.119551	0.25501	0.166787
50	0.000322	0.001923	0.005837	0.000899	0.004253	0.073874	1.244337	0.002973	0.046098	0.193939	0.182214
51	0.000454	0.000409	0.002372	0.003258	3.87E-05	0.083064	1.263569	0.006625	0.043069	0.188766	0.179117
52	0.00084	0.001212	0.003889	0.000442	0.000315	0.103459	1.0955	0.014152	0.070606	0.189544	0.159456
53	0.016634	0.001719	0.016732	0.793561	0.004027	0.138917	0.001262	0.185789	0.002368	0.30772	0.001421
54	0.040607	0.001293	0.014665	0.481211	0.000562	0.068786	0.065395	0.507409	4.94E-05	0.165274	0.015808
55	0.003657	0.104541	0.003604	1.040364	0.000624	0.128106	0.022725	0.001901	0.003192	0.325109	0.001402

56	0.001445	0.14072	0.011406	0.921136	0.002895	0.125702	0.010063	0.001058	0.00097	0.342307	0.002897
57	0.00177	0.088912	0.009961	1.298531	0.001599	0.120717	0.02298	0.013117	0.001715	0.237427	0.003485
58	0.06489	0.000217	0.003485	0.001432	0.03883	0.392216	0.062053	0.732507	0.000484	0.128923	0.019298
59	0.019921	0.000813	0.000647	0.000868	0.004201	0.073595	0.243954	0.88191	0.003142	0.265512	0.028525
60	0.064077	1.74E-05	0.00992	0.00159	0.016466	0.138561	0.326443	0.594806	0.002162	0.107624	0.077661
61	0.053984	0.000671	0.000455	0.002696	0.043093	0.3605	0.00729	1.031689	0.000479	0.139007	0.001122
62	0.065598	0.000611	0.001086	0.003003	0.05737	0.540818	0.008498	0.680715	0.001121	0.127341	0.000983
63	0.077766	0.001036	0.000163	0.009004	0.019434	0.193272	0.003081	1.379115	0.000897	0.178449	0.001625
64	0.001675	0.01308	0.077039	0.107675	0.005981	0.527583	0.033185	0.059698	0.001356	0.594837	0.002586
65	0.069298	0.001278	0.002348	0.001837	0.005182	0.145421	0.140705	0.78133	0.00134	0.249344	0.035989
66	0.10295	0.000504	0.001593	0.003553	0.002691	0.144169	0.005991	1.524694	2.09E-05	0.192146	0.001274
67	0.087147	0.001186	0.000234	0.004505	0.008762	0.17284	0.003338	1.29019	0.000792	0.232795	0.001574
68	0.062246	0.000925	0.002244	0.000804	0.043295	0.506	0.003896	0.723594	0.000984	0.151329	0.003858
69	0.029191	0.001016	0.007682	0.055327	0.004453	0.5886	0.197234	0.273115	0.0023	0.123589	0.046113
70	0.008162	0.000451	0.000677	0.228447	0.00758	0.303062	0.457948	0.073087	0.002344	0.104147	0.093896
71	0.038211	0.00067	0.004996	0.171539	0.00473	0.247632	0.24029	0.288309	0.001676	0.155737	0.057077
72	0.028547	0.003006	0.013142	0.055954	0.020742	1.094311	0.084893	0.192772	0.002893	0.125187	0.006481
73	0.016082	0.0046	0.006557	0.041822	0.006145	0.4129	0.536109	0.096164	0.012574	0.099289	0.105119
74	0.010323	0.000786	0.015074	0.043346	0.007609	0.813846	0.329731	0.078278	0.001299	0.107618	0.060831
75	0.003643	0.047311	0.00208	0.125964	0.001074	0.15261	0.02432	0.35608	0.143774	0.273899	0.083117
76	0.002404	0.070188	0.001496	0.186155	0.00201	0.09199	0.03099	0.239419	0.282605	0.25405	0.042957
77	0.000762	0.06593	0.00309	0.142652	0.003277	0.161471	0.01113	0.21099	0.310873	0.254268	0.043913
78	0.000183	0.064404	0.002041	0.144332	0.004349	0.137474	0.02428	0.245369	0.271876	0.255579	0.050324
79	0.001868	0.063778	0.005163	0.167917	0.002887	0.12175	0.013102	0.237106	0.277701	0.262225	0.050606
80	0.003982	0.058216	0.006345	0.05995	0.004268	0.235186	0.174697	0.019966	0.054443	0.752838	0.035284
81	0.003837	0.12127	0.003064	0.848478	0.004678	0.207269	0.002794	0.00016	0.000796	0.324183	0.001057
82	0.002979	0.174337	0.00115	0.795634	0.00201	0.141378	0.012129	0.000196	0.001974	0.355473	0.002064
83	0.002055	0.18681	0.005423	1.003405	0.000786	0.085797	0.004686	0.001123	0.006111	0.317595	0.0016

Table S8. The results of the trifluoromethylation explorative optimization (see Section 5.5.2).

Add_3-volume	Add_4-volume	Add_7-dispense_speed	Add_7-volume	Stir_8-time	spectrum_peak-area_1633
3.5	5	0.2	5	1800	-0.4779
4.9674	4.71	7.7265	3.3728	2388.626	-0.5818
3.4371	4.9868	0.3145	4.9603	1869.3718	-0.6005
2.2097	1.958	3.0766	4.7902	5470.981	-0.6115
3.4669	4.993	0.2603	4.9791	1836.5115	-0.5505
4.2991	4.9903	6.9041	0.8012	3924.3557	-0.4984
3.5232	5	0.1512	4.9846	1800	-0.4956
2.6742	2.5729	6.9966	3.5736	7151.0801	-0.5769
3.4835	4.9965	0.2301	4.9896	1818.2557	-0.4694
1.1514	3.5737	4.1572	2.1582	3611.7249	-0.6829
3.4905	4.9984	0.2401	4.9955	1827.9069	-0.1065
3.4795	4.9887	0.2172	4.9936	1813.7491	-0.487
3.4489	0.5034	2.1559	0.723	1832.848	-0.7141
3.4959	5	0.2475	4.9875	1830.6141	-0.4692
3.5172	1.4056	9.2389	3.4393	6216.7085	-0.3913
3.6716	4.9948	0.4108	4.9123	1820.5065	-0.5042
1.8718	2.342	3.604	2.7266	6713.3198	-0.5905
3.4974	4.9916	0.25	4.9978	1827.2998	-0.4758
3.1952	0.7773	1.0382	1.5286	4568.1348	-0.5463
3.4868	4.9971	0.2442	5	1833.4457	-0.4422
2.2324	3.1281	5.4343	4.0445	6846.0322	-0.5695
3.4943	4.9994	0.2325	4.9989	1824.4208	-0.4662
0.6164	4.0719	0.3935	1.2007	6975.1602	-0.7717
3.4915	4.9935	0.2369	4.9967	1823.3331	-0.4991
4.8554	4.3793	4.5024	4.4297	2804.3989	-0.4978

3.6246	4.8424	0.0539	4.8127	1829.4752	-0.2498
2.9257	1.6921	6.439	1.9837	5734.5981	-0.6364
3.5829	4.8928	0.0884	4.8994	1825.9874	-0.3692
2.3663	2.667	8.4578	3.866	4308.7773	-0.7029
3.4929	4.9978	0.2425	4.9918	1828.5566	-0.5252
1.5091	1.1805	3.9048	3.408	5943.9067	-0.8197
3.4889	4.9967	0.2389	4.9944	1822.7146	-0.6165
3.2902	0.6341	9.7743	1.8064	3343.2942	-0.7428
3.4877	4.9986	0.2409	4.9954	1831.5872	-0.6499
3.8507	3.0556	2.102	2.8947	5062.3755	-0.5077
3.4937	4.9995	0.2456	4.993	1827.9785	-0.6288
1.66	3.3253	1.7386	2.2343	2583.7048	-0.7448
3.3586	4.8507	0.0881	4.9948	1829.0911	-0.4967
2.0985	2.4198	4.9677	1.3539	3030.051	-0.7682
3.4878	4.9952	0.2376	4.9974	1832.2788	-0.6978

Table S9. Results for the optimization of the Sodium 4-vinylbenzenesulfonate catalytic oxidation.

Add_0-volume	Add_2-volume	HeatChillToTemp_4-temp	Stir_5-time	multie_purity-area_14	Rel. Peak Area	Purity
5.930363	0.281536	53.90767	5374.944	0.936862	11.12758	0.69275
5.125	0.231	60	1800	1.001846	12.09507	0.742094
5.577005	0.170647	56.5265	10761.03	0.960348	10.98935	0.717526
4.374598	0.23139	26.359	8101.53	1.014233	11.51263	0.764424
5.235639	0.155013	56.21	3598.83	1.018665	12.07292	0.750825
4.205514	0.251755	47.3135	9444.6	0.937303	10.6126	0.726961
5.552159	0.188565	39.0535	2673.63	1.03046	11.84036	0.754814
6.0278	0.252029	62.8975	6748.92	0.931288	10.71362	0.689388
4.36937	0.211784	70.9005	4508.82	0.961356	10.89795	0.737969

5.751849	0.210262	29.935	6050.52	1.022144	11.72645	0.747777
4.878836	0.154	25	1800	0.951958	10.57003	0.755554
6.14998	0.281537	25	10761.03	1.001754	11.5991	0.731457
6.14998	0.281537	25	1800	1.011689	11.66346	0.754415
4.672586	0.259892	53.8685	5742.81	0.946677	10.72659	0.724072
4.36937	0.281537	38.442	1800	1.023849	11.6238	0.773037
4.672586	0.259892	53.8685	5742.81	0.943857	10.72815	0.725548
6.143563	0.154	46.189	1800	1.024607	11.73885	0.753196
4.590647	0.202877	61.157	8210.7	0.957861	10.94852	0.729597
4.36937	0.189985	47.9565	1800	1.028457	11.62418	0.776575
4.675189	0.192694	31.5975	5844.51	1.025144	11.56126	0.769117
6.14998	0.202228	50.8665	1800	0.665637	6.807097	0.626507
5.987927	0.174625	25	4362.12	0.83316	9.003038	0.698722
5.6375	0.1925	67.5	2693.7	0.994121	11.24234	0.739176
4.36937	0.281537	25	1800	1.03148	11.59027	0.782197
5.253412	0.308	25	3598.83	1.028255	11.57735	0.76337
4.6125	0.1925	67.5	2693.7	0.99393	11.11633	0.752316

Table S10. The results of the Van Leusen Oxazole synthesis.

Add_2-volume	Add_3-volume	HeatChillToTemp_4-temp	Wait_5-time	spectrum_integration_area_16.7..16.9	Yield
1.35	2.19	102	24100	0.0128	0.296444
1.63	2.27	97	5780	0.0253	0.074886
1.35	0.49	115	21300	0.00345	0.116744
1.4	2.09	108	22600	0.00432	0.217785
1.59	0.7	108	20800	0.00684	0.246672
1.65	1.97	109	22300	0.00816	0.284063
1.34	2.04	36	21000	0.0056	0.29567

0.62	2.44	105	21300	0.00367	0.209791
1.5	2.52	107	20500	0.00679	0.260611
1.56	1.77	53	21400	0.0135	0.286446
1.83	2.14	115	21000	0.00956	0.295236
1.21	2.06	107	20900	0.0114	0.308481
1.61	2.07	111	20400	0.0164	0.333734
1.67	2.32	107	21300	0.0171	0.342697
1	1	140	14400	0.0093	0.26773
1.59	2.19	154	21200	0.00148	0.024947
1.2	5	144	26400	0.0112	0.025476
0.51	3.47	150	3820	0.0124	0.043266
1.88	4.49	42	12200	0.0135	0.036389
0.77	4.28	90	9730	0.0259	0.047131
1.48	4.14	102	28800	0.0517	0.045649
0.35	4.11	85	10500	0.00909	0.03232
1.6	2.13	108	28700	0.0422	0.065295
1.4	4.22	69	11300	0.0629	0.069571
1.61	2.14	106	13100	0.0482	0.07256
1.33	4.08	65	17900	0.0619	0.072275
1.35	4.06	71	16300	0.0656	0.074072
1.36	4.09	73	19300	0.0646	0.085893
1.36	4.11	70	9330	0.031	0.369044
1.35	4.06	71	16300	0.0329	0.436853
1.35	4.06	71	16300	0.0321	0.432427
1.35	4.06	71	16300	0.0287	0.431138

Table S11. The results of the tosMIC benzylidenemalononitrile reaction.

Add_2-volume	Add_3-volume	Add_4-volume	HeatChillToTemp_5-temp	Wait_6-time	spectrum_integration_area_15.0..15.2	Yield
1.00	2.00	2.00	25.00	86400.00	3.23	0.27
1.00	2.00	2.00	25.00	14400.00	2.28	0.19018
1.50	1.11	4.51	98.00	14800.00	2.42	0.20262
1.85	3.24	5.68	100.00	8250.00	2.28	0.19031
1.54	2.45	0.22	42.00	20100.00	3.72	0.31088
1.54	1.37	4.49	132.00	3870.00	1.63	0.13662
1.42	0.43	2.71	59.00	17800.00	1.72	0.14338
1.13	0.79	4.69	149.00	13500.00	0.651	0.05444
1.41	2.16	1.98	63.00	18500.00	2.11	0.17669
0.46	3.54	5.99	39.00	28700.00	0.636	0.05313
1.85	4.00	0.00	25.00	20500.00	4.85	0.40523
2.00	4.00	0.00	108.00	28800.00	5.26	0.43968
2.00	4.00	0.00	160.00	3600.00	1.97	0.16475
1.00	2.00	2.00	25.00	14400.00	2.28	0.19018
1.50	1.11	4.51	98.00	14800.00	2.42	0.20262
1.85	3.24	5.68	100.00	8250.00	2.28	0.19031
1.54	2.45	0.22	42.00	20100.00	3.72	0.31088
1.54	1.37	4.49	132.00	3870.00	1.63	0.13662
1.42	0.43	2.71	59.00	17800.00	1.72	0.14338
1.13	0.79	4.69	149.00	13500.00	0.651	0.05444
1.41	2.16	1.98	63.00	18500.00	2.11	0.17669
0.46	3.54	5.99	39.00	28700.00	0.636	0.05313
1.85	4.00	0.00	25.00	20500.00	4.85	0.40523
2.00	4.00	0.00	108.00	28800.00	5.26	0.43968
2.00	4.00	0.00	160.00	3600.00	1.97	0.16475
2.00	2.55	1.79	44.00	28800.00	3.77	0.3149
1.98	4.00	0.00	89.00	27700.00	3.13	0.26183
0.20	2.80	0.00	36.00	28800.00	0.352	0.02946

2.00	4.00	6.00	25.00	28800.00	3.57	0.29854
2.00	4.00	0.00	25.00	28800.00	5.86	0.48977
2.00	0.40	0.00	25.00	28800.00	3.8	0.31763
2.00	4.00	0.00	25.00	28800.00	5.57	0.46544
2.00	4.00	0.00	25.00	28800.00	0.0507	0.00423
2.00	0.40	6.00	160.00	28800.00	1.41	0.11797
2.00	0.40	0.00	25.00	3600.00	0.304	0.02538
2.00	4.00	6.00	160.00	28800.00	1.01	0.08448
2.00	0.40	0.00	160.00	28800.00	2.33	0.19453
2.00	3.96	0.00	25.00	23900.00	5.72	0.47788
2.00	4.00	0.00	25.00	22100.00	5.68	0.47441
2.00	4.00	0.00	25.00	20800.00	5.62	0.46982
2.00	4.00	0.00	25.00	20400.00	4.77	0.39833
2.00	4.00	0.00	25.00	20200.00	4.85	0.40522
2.00	4.00	0.00	25.00	20200.00	0.0247	0.00207
2.00	4.00	0.00	25.00	28800.00	5.97	0.49939
3.00	6.00	0.00	20.00	36000.00	7.09	0.59301
3.00	6.00	0.00	10.00	21600.00	8.03	0.67103
3.00	6.00	0.00	5.00	28800.00	9.25	0.77287
3.00	6.00	0.00	15.00	50400.00	7.15	0.59755
4.00	8.00	0.00	0.00	86400.00	4.72	0.39484
3.88	8.00	0.00	0.00	3600.00	4.88	0.40791

Table S12. The results of the reaction between phloroglucinol, benzylidenemalononitrile and 1,8-bis(dimethylamino)naphthalene.

10 Appendix E

Hardware graphs for running dynamic experiments.

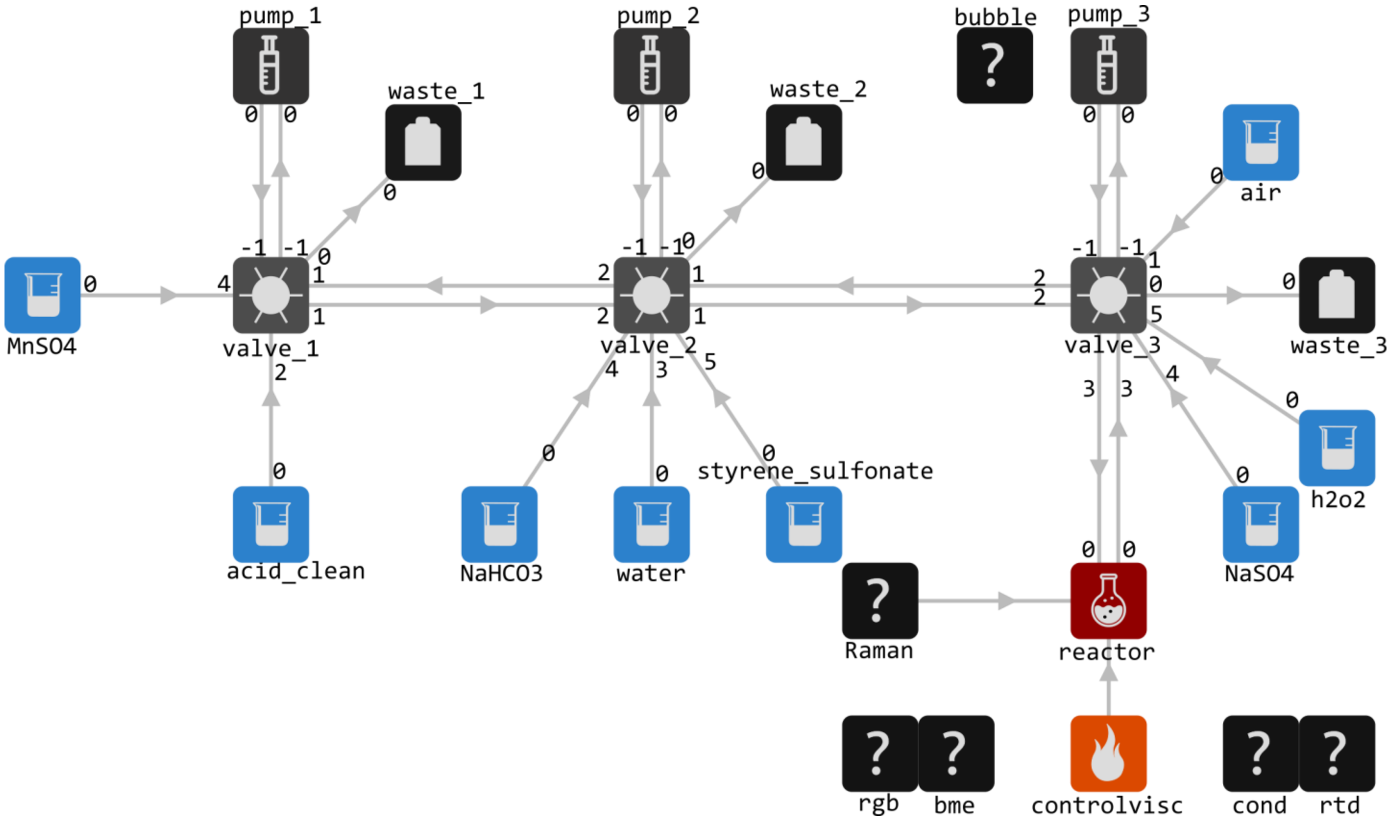

Fig. S54. Hardware graph used for the Raman-monitored catalytic oxidation reaction of the Sodium 4-vinylbenzenesulfonate.

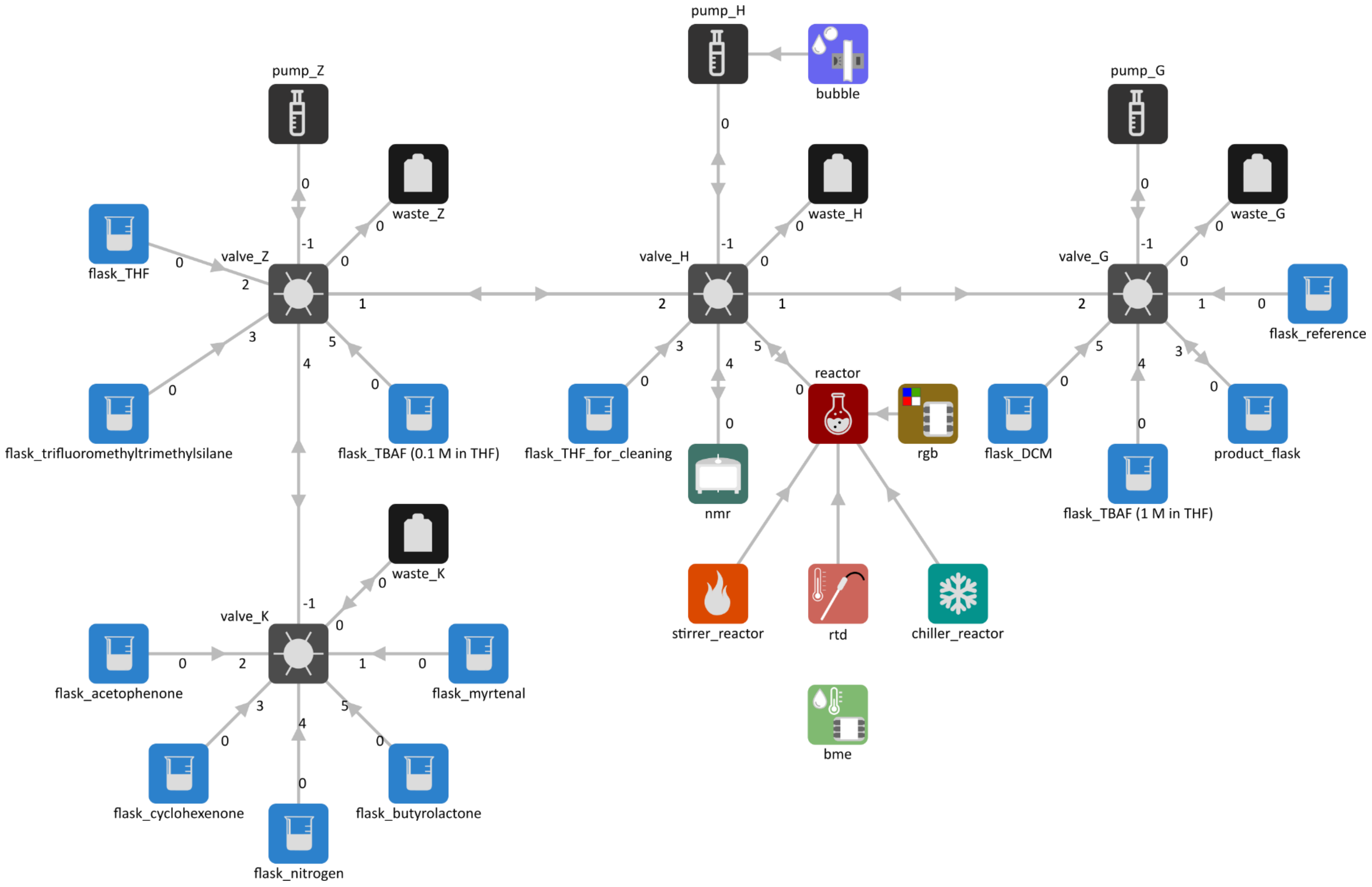

Fig. S55. Hardware graph for the explorative optimization of the trifluoromethylation reaction.

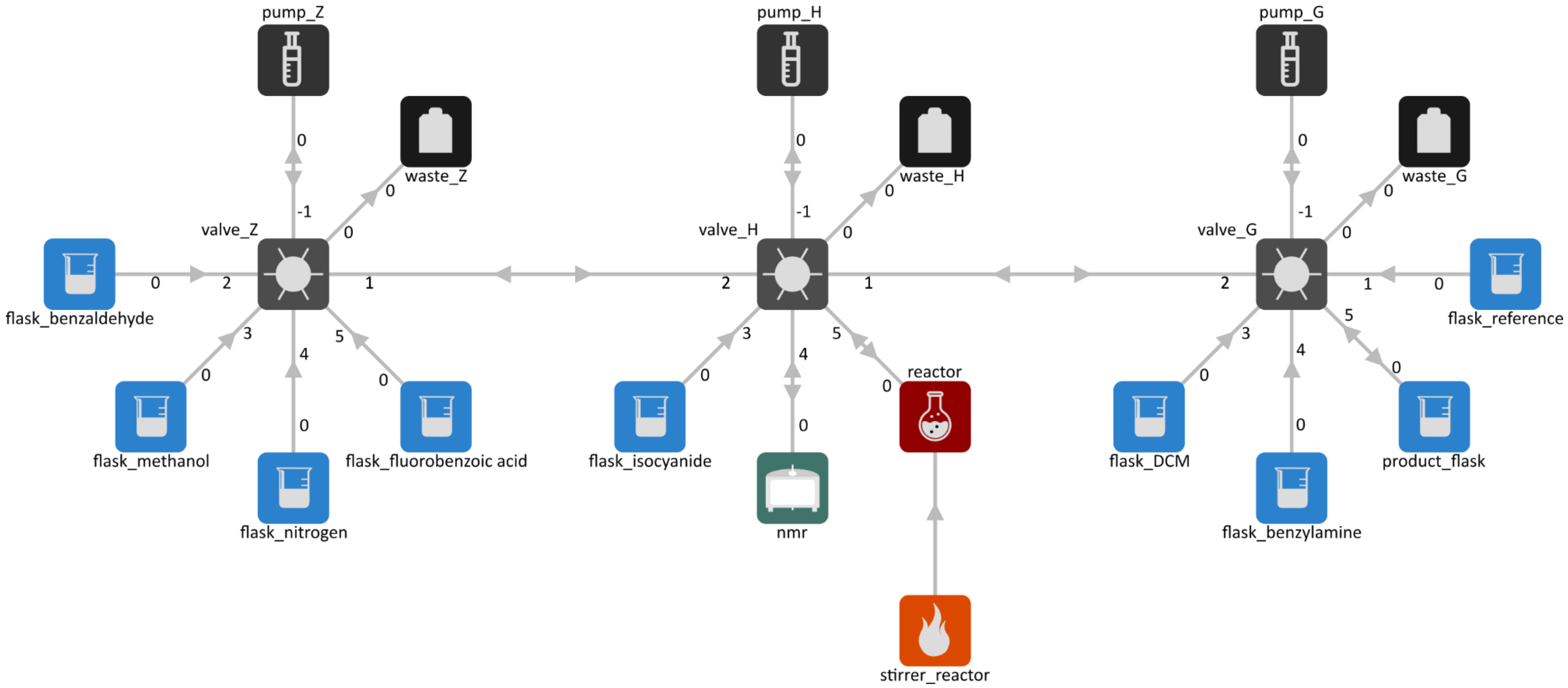

Fig. S56. Hardware graph for the optimization of the Ugi reaction.

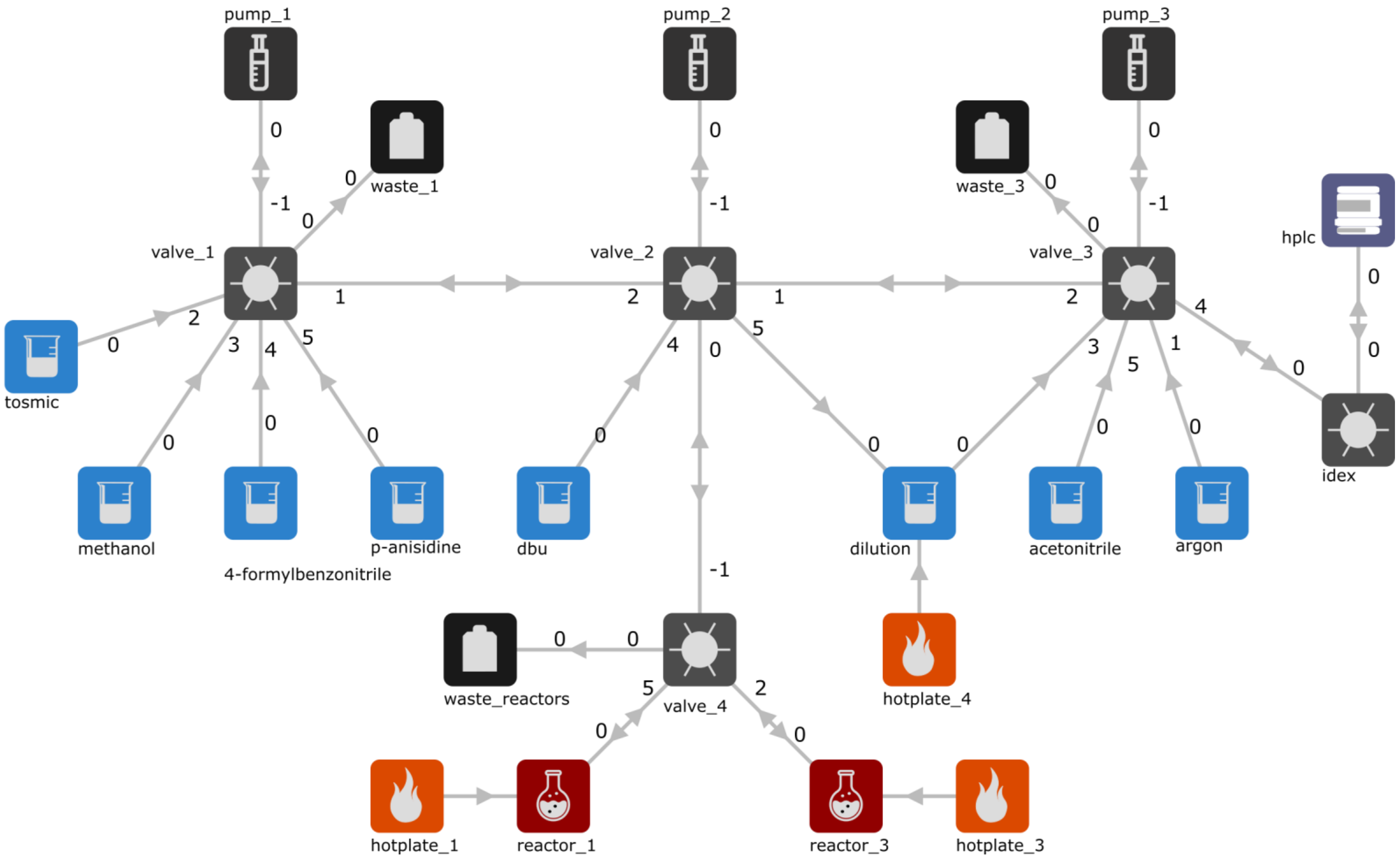

Fig. S57. Hardware graph for the optimization of the Van Leusen reaction.

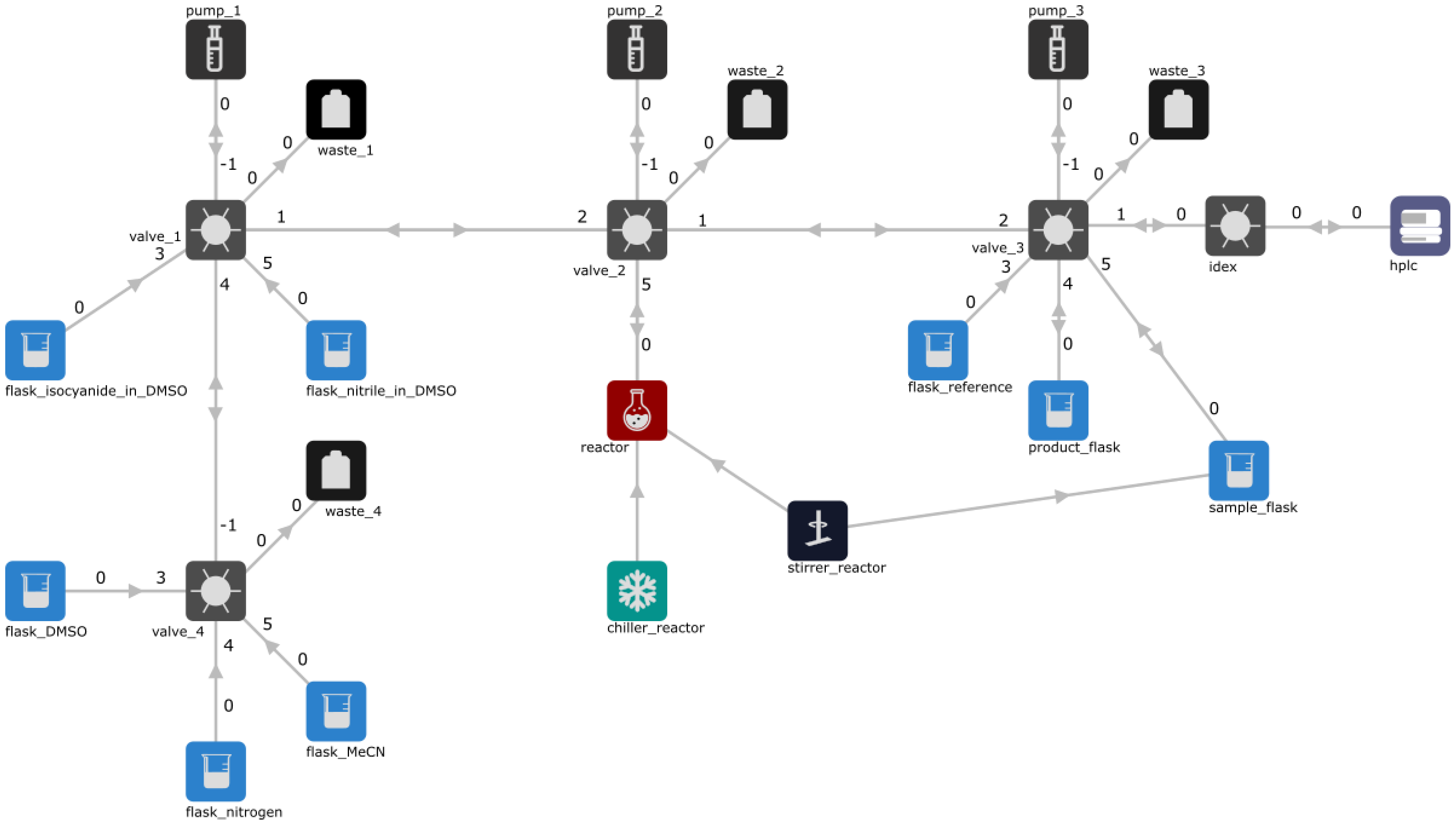

Fig. S58. Hardware graph for the optimization of the tosMIC and phloroglucinol reaction.

Supplementary References

1. ECMA-404. The JSON data interchange syntax (2017), https://www.ecma-international.org/publications-and-standards/standards/ecma-404/
2. Despite ECMA-404 standard for JSON format not allowing "Infinity", this value is correctly parsed with python and therefore is accepted as the target value.
3. Mehr, S. H. M., Craven, M., Leonov, A. I., Keenan, G. & Cronin, L. A universal system for digitization and automatic execution of the chemical synthesis literature. *Science* **370**, 101 (2020).
4. Zorin, V., Bernstein, M. A. & Cobas, C. A robust, general automatic phase correction algorithm for high-resolution NMR data. *Magnetic Resonance in Chemistry* **55**, 738-746 (2017).
5. Harris, C. R. *et al.* Array programming with NumPy. *Nature* **585**, 357-362 (2020).
6. Head, T., Kumar, M., Nahrstaedt, H., Louppe, G. & Shcherbatyi, I. *scikit-optimize/scikit-optimize* v. v0.8.1 (Zenodo, 2020).
7. *API reference to run bayesian optimization loop.*, https://scikit-optimize.github.io/stable/modules/generated/skopt.Optimizer.html (accessed August 2021).
8. *pyDOE2: An experimental design package for python*, https://github.com/clicumu/pyDOE (accessed December 2021).
9. *The Python Standard Library*, https://docs.python.org/3/library/ (accessed August 2021).
10. Häse, F. *et al.* Olympus: a benchmarking framework for noisy optimization and experiment planning. *Machine Learning: Science and Technology* **2**, 035021 (2021).
11. Eilers, P. & Boelens, H. *Baseline Correction with Asymmetric Least Squares Smoothing.* (2005).
12. Helmus, J. J. & Jaroniec, C. P. Nmrglue: an open source Python package for the analysis of multidimensional NMR data. *Journal of Biomolecular NMR* **55**, 355-367 (2013).
13. *Arduino Mega 2560 Rev3 Datasheet*, https://docs.arduino.cc/static/2a5af72674da26b40f0312c1b3e499a2/A000067-datasheet.pdf (accessed January 2022).
14. *TCS34725 Colour Sensor*, https://ams.com/en/tcs34725 (accessed September 2021).
15. *Humidity sensor BME280*, https://www.bosch-sensortec.com/products/environmental-sensors/humidity-sensors-bme280/ (accessed December 2021).
16. *OPB350 - Tube liquid sensor*, https://www.ttelectronics.com/products/categories/optoelectronics/optoelectronics/opb350/ (accessed December 2021).
17. *MAX31865 RTD-to-Digital Converter*, https://www.maximintegrated.com/en/products/interface/sensor-interface/MAX31865.html (accessed December 2021).
18. *Commanduino Library*, https://github.com/croningp/commanduino (accessed December 2021).
19. *Arduino CommandTools*, https://github.com/croningp/Arduino-CommandTools (accessed December 2021).
20. Wang, Z., Simoncelli, E. P. & Bovik, A. C. in *The Thrity-Seventh Asilomar Conference on Signals, Systems & Computers, 2003.* 1398-1402 Vol.1392.
21. *Dash Python User Guide*, https://dash.plotly.com/ (accessed January 2021).
22. Angelone, D. *et al*. Convergence of multiple synthetic paradigms in a universally programmable chemical synthesis machine. *Nature Chemistry* 13, 63–69 (2021).

23 Xiang, Z. *et al.* Concise Synthesis of Isoquinoline via the Ugi and Heck Reactions. *Organic Letters* **6**, 3155-3158 (2004).

24 Singh, K., Kaur, A., Mithu, V. S. & Sharma, S. Metal-Free Organocatalytic Oxidative Ugi Reaction Promoted by Hypervalent Iodine. *The Journal of Organic Chemistry* **82**, 5285-5293 (2017).

25 Campos, S. A., Harling, J. D. M., Afjal Hussain & Smith, I. E. D. Benzothiophene derivatives as estrogen receptor inhibitors WO 2015/000867 Al (2013).

26 Hage, R. & Lienke, A. Applications of Transition-Metal Catalysts to Textile and Wood-Pulp Bleaching. *Angewandte Chemie International Edition* **45**, 206-222 (2006).

27 Lane, B. S., Vogt, M., DeRose, V. J. & Burgess, K. Manganese-catalyzed epoxidations of alkenes in bicarbonate solutions. *J. Am. Chem. Soc.* **124**, 11946-11954 (2002).

28 Abdolahzadeh, S. *Catalysis in complex media: Analytical approaches and mechanisms in manganese catalysed oxidations*, University of Groningen, (2015).

29 Häse, F., Roch, L. M., Kreisbeck, C. & Aspuru-Guzik, A. Phoenics: A Bayesian Optimizer for Chemistry. *ACS Cent. Sci.* **4**, 1134-1145 (2018).

30 Prakash, G. K. S., Krishnamurti, R. & Olah, G. A. Synthetic methods and reactions. 141. Fluoride-induced trifluoromethylation of carbonyl compounds with trifluoromethyltrimethylsilane (TMS-CF3). A trifluoromethide equivalent. *Journal of the American Chemical Society* **111**, 393-395 (1989).

31 Johnston, C. P. *et al.* Anion-Initiated Trifluoromethylation by TMSCF3: Deconvolution of the Siliconate-Carbanion Dichotomy by Stopped-Flow NMR/IR. *J. Am. Chem. Soc.* **140**, 11112-11124 (2018).
